# Treatment with HC-070, a potent inhibitor of TRPC4 and TRPC5, leads to anxiolytic and antidepressant effects in mice

**DOI:** 10.1371/journal.pone.0191225

**Published:** 2018-01-31

**Authors:** Stefan Just, Bertrand L. Chenard, Angelo Ceci, Timothy Strassmaier, Jayhong A. Chong, Nathaniel T. Blair, Randall J. Gallaschun, Donato del Camino, Susan Cantin, Marc D’Amours, Christian Eickmeier, Christopher M. Fanger, Carsten Hecker, David P. Hessler, Bastian Hengerer, Katja S. Kroker, Sam Malekiani, Robert Mihalek, Joseph McLaughlin, Georg Rast, JoAnn Witek, Achim Sauer, Christopher R. Pryce, Magdalene M. Moran

**Affiliations:** 1 Boehringer Ingelheim Pharma GmbH & Co. KG, Biberach an der Riss, Germany; 2 Hydra Biosciences, Cambridge, Massachusetts, United States of America; 3 Preclinical Laboratory for Translational Research into Affective Disorders, Department of Psychiatry, Psychotherapy & Psychosomatics, Psychiatric Hospital, University of Zurich, Zurich, Switzerland; Indiana University School of Medicine, UNITED STATES

## Abstract

**Background:**

Forty million adults in the US suffer from anxiety disorders, making these the most common forms of mental illness. Transient receptor potential channel canonical subfamily (TRPC) members 4 and 5 are non-selective cation channels highly expressed in regions of the cortex and amygdala, areas thought to be important in regulating anxiety. Previous work with null mice suggests that inhibition of TRPC4 and TRPC5 may have anxiolytic effects.

**HC-070 *in vitro*:**

To assess the potential of TRPC4/5 inhibitors as an avenue for treatment, we invented a highly potent, small molecule antagonist of TRPC4 and TRPC5 which we call HC-070. HC-070 inhibits recombinant TRPC4 and TRPC5 homomultimers in heterologous expression systems with nanomolar potency. It also inhibits TRPC1/5 and TRPC1/4 heteromultimers with similar potency and reduces responses evoked by cholecystokinin tetrapeptide (CCK-4) in the amygdala. The compound is >400-fold selective over a wide range of molecular targets including ion channels, receptors, and kinases.

**HC-070 *in vivo*:**

Upon oral dosing in mice, HC-070 achieves exposure levels in the brain and plasma deemed sufficient to test behavioral activity. Treatment with HC-070 attenuates the anxiogenic effect of CCK-4 in the elevated plus maze (EPM). The compound recapitulates the phenotype observed in both null TRPC4 and TRPC5 mice in a standard EPM. Anxiolytic and anti-depressant effects of HC-070 are also observed in pharmacological in vivo tests including marble burying, tail suspension and forced swim. Furthermore, HC-070 ameliorates the increased fear memory induced by chronic social stress. A careful evaluation of the pharmacokinetic-pharmacodynamic relationship reveals that substantial efficacy is observed at unbound brain levels similar to, or even lower than, the 50% inhibitory concentration (IC_50_) recorded in vitro, increasing confidence that the observed effects are indeed mediated by TRPC4 and/or TRPC5 inhibition. Together, this experimental data set introduces a novel, high quality, small molecule antagonist of TRPC4 and TRPC5 containing channels and supports the targeting of TRPC4 and TRPC5 channels as a new mechanism of action for the treatment of psychiatric symptoms.

## Introduction

According to the World Health Organization, approximately 800,000 people commit suicide each year and many more attempt it. Among people aged 15–29, suicide is the second leading cause of death [1]. Many of these individuals suffer from anxiety and/or depression. While improved access to counseling, prevention programs, and existing medications would likely reduce these deaths, novel treatment options are also of importance. In the STAR*D trial of more than 4,000 patients with nonpsychotic depression approximately 30% of the patients failed to reach remission even after treatment with four different medications [2] and continued to experience residual symptoms [3] that significantly impacted their quality of life.

TRPC4 and TRPC5 are non-selective cation channels that are widely expressed throughout the brain, with particularly high transcript levels in the cortex and amygdala [4]. They can exist as homomultimers or as heteromultimers with TRPC1 [5]. Stimulation of G-alpha q type G-protein coupled receptors (GPCRs) induces TRPC4 and TRPC5 currents in heterologous expression systems [6] and is thought to be a physiologically relevant activator of the channels [7, 8]. Activation of G-alpha q coupled-GPCRs has also been implicated in fear-related behaviors, and acute engagement of the signaling pathway via a synthetic receptor is sufficient to induce behaviors indicative of anxiety [9]. G-alpha i/o coupled GPCRs also have the ability to activate TRPC4, perhaps even more robustly than G-alpha q receptors, positioning these channels as transducers of an even broader array of signals [10, 11].

Genetic deletion of either TRPC4 [8] or TRPC5 [7] reduces anxiety behaviors in mice. Null animals show increased exploratory behaviors in both open field tests and in the elevated plus maze (EPM) [7, 8]. These findings were recapitulated with early pharmacological antagonists [12]. Some of the observed effects may be due to a reduction in cholecystokinin receptor B (CCKB) and metabotropic glutamate receptor (mGluR) signaling in the amygdala, as brain slices from null mice show attenuated responses to activation of those receptors [7, 8]. These data suggest that TRPC4 and TRPC5 are downstream effectors of these two GPCR pathways.

While some small molecule inhibitors of TRPC4 and TRPC5 have been described, these probes have suffered from a lack of potency, inhibiting channels only at micromolar (μM) levels [12, 13]. This relative lack of potency makes assessing the specificity of these compounds difficult. In addition, little has been reported about the pharmacokinetic (PK) properties of these molecules.

Based on the potential biological importance of TRPC4 and TRPC5 and the lack of high potency inhibitors, we sought to develop a high-quality compound suitable to elucidate the in vivo function of these channels in the CNS. To this end, we performed a fluorescence-based high throughput screen, confirmed compounds of interest with electrophysiology, and optimized the potency and PK properties of the compounds through iterative cycles of compound synthesis, testing, and design. One of the resulting compounds was HC-070. HC-070 potently and selectively inhibits TRPC4 and TRPC5. The in vitro properties of this compound are very similar to a compound we previously invented and disclosed, HC-608 [14, 15], which has also been referred to as Pico145 [16]. In addition to blocking heterologously expressed channels, we show that HC-070 attenuates neuronal activity recorded from the amygdala.

Oral dosing of HC-070 results in brain and plasma exposures sufficient to test behavioral activity. We characterized the behavioral effects of HC-070 treatment in the EPM test with and without CCK-4 challenge, the marble-burying test, and a model of chronic social stress that induced hyper-fear learning and memory. In each of these animal models, treatment with HC-070 reduces anxiety/fear behaviors without impacting locomotor activity. HC-070 also has antidepressant-like effects in the tail suspension and forced swim tests, where it significantly reduces immobility. Detailed modeling of the pharmacokinetic-pharmacodynamic (PK-PD) relationship indicates that efficacy in the forced swim test coincides with unbound brain levels near the IC_50_ for TRPC4 and TRPC5 inhibition and further substantiates the finding that the observed behavioral effects are on target.

Taken together, these data suggest that TRPC4 and TRPC5 channels may be important targets for the treatment of anxiety and depression. HC-070 is a potent and selective compound which should be useful in further unraveling the function of these channels.

## Results

### In vitro profiling

**Fig 1:**
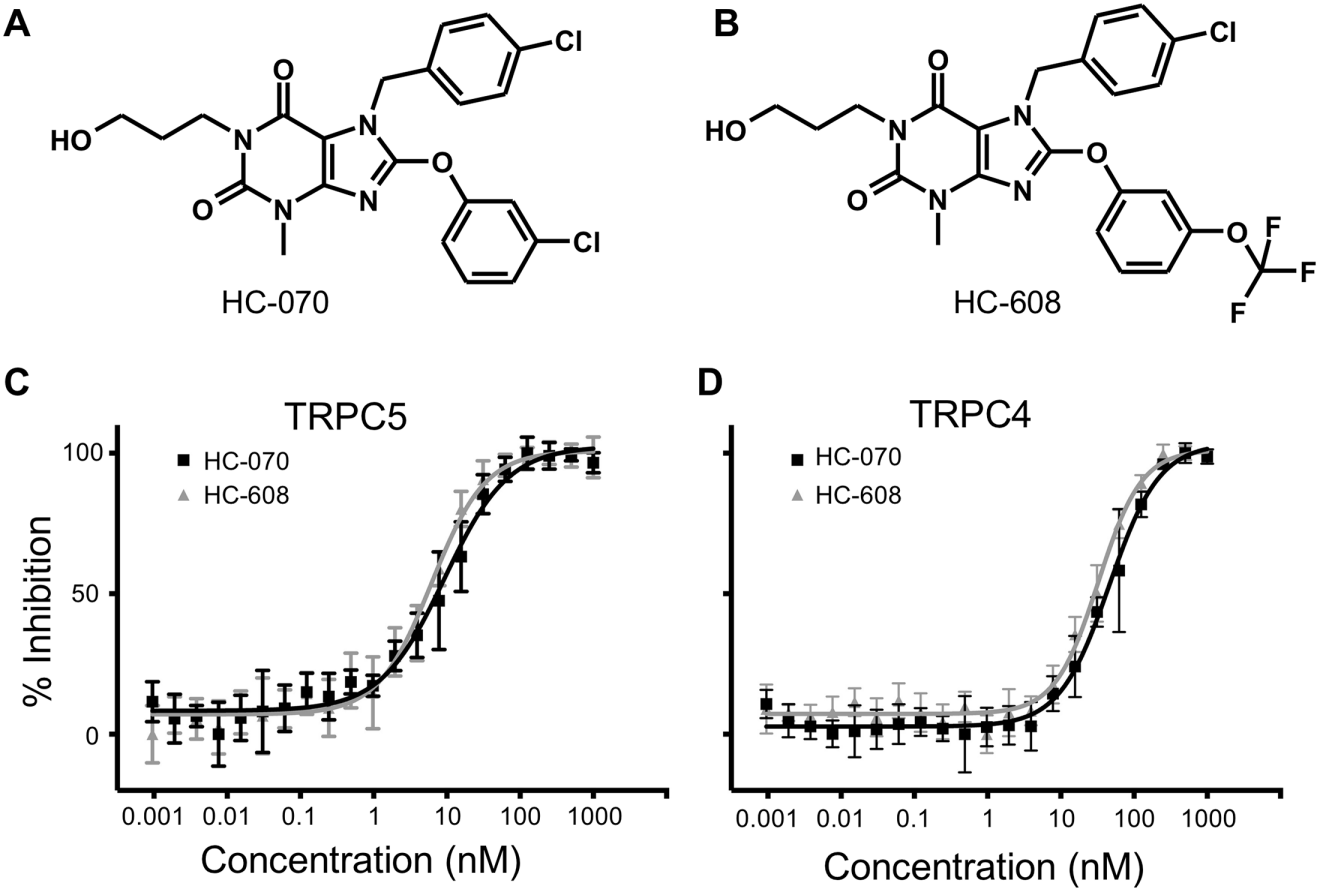
Structures and activity in fluorometric assays of HC-070 and HC-608. The chemical structures of (A) HC-070 and (B) HC-608 (Pico145). (C) Inhibition of hTRPC5 by HC-070 and HC-608 in indicator-assisted calcium influx analysis. Concentrations tested ranged from 1 picomolar (pM) to 1 μM. Each data point represents the average of 8 measurements from a 384-well plate. Error bars show the standard deviation. The IC_50_ values were 9.3 ± 0.9 and 6.2 ± 0.5 nanomolar (nM), respectively. (D) Inhibition of hTRPC4 by HC-070 and HC-608 over the same range of concentrations. The IC_50_ values were 46.0 ± 3.9 nM and 32.5 ± 1.8 nM, respectively (n = 8). Error bars represent the standard deviation.

In order to identify small molecule inhibitors of TRPC5, we performed a high-throughput screen of ~65,000 compounds. The average Z’, a metric of assay robustness, was 0.65 ± 0.07, indicating very good separation between the positive and negative controls. This effort resulted in the identification of several classes of compounds that were subsequently confirmed as being TRPC5 inhibitors in electrophysiology. We chose the class of compounds that we thought exhibited the best combination of potency and drug-like properties and performed iterative cycles of compound design, synthesis, and testing to identify a high-quality inhibitor. This effort resulted in the invention of HC-070 (Fig 1 panel A) [14, 15]. This compound is highly similar to HC-608 (Fig 1 panel B), which we also invented during this effort [12, 14, 15], and was recently published as Pico145 [16]. Both compounds are readily synthesizable in three steps from commercially available starting materials [14].

HC-070 inhibited calcium influx into recombinantly-expressed human TRPC5 (hTRPC5) (Fig 1 panel C) expressing cells with an IC_50_ of 9.3 ± 0.9 nM in a concentration-response study performed in octuplicate, covering concentrations ranging from 1 pM to 1 μM. More than 90% inhibition was observed at concentrations of 31 nM or above, with complete block at the highest concentrations. HC-070 also attenuated TRPC4-activity, inhibiting calcium influx with an IC_50_ of 46.0 ± 3.9 nM (Fig 1 panel D). Again, complete inhibition was observed. Comparable results were achieved with HC-608 (Fig 1 panels C and D). Example time course data from cells expressing TRPC5 are shown in S1 Fig panels A and B. Example time course data from cells expressing TRPC4 are shown in S1 Fig panels C and D.

In whole-cell manual patch clamp, HC-070 inhibited lanthanum-activated hTRPC5-mediated currents with an IC_50_ of 0.52 nM (Table 1). This is significantly more potent than the IC_50_ we observed in the fluorometric assay. Increased potency in electrophysiological compared to fluorometric assays has often been observed and reported for other TRP channels [17].

**Table 1:** HC-070 inhibits recombinantly expressed TRPC4 and TRPC5, as well as TRPC1-containing heteromultimers, in whole-cell manual patch clamp.

		HC-070	
CHANNEL	CURRENT ACTIVATION	Cells Tested	IC_50_ (nM)
mTRPC5	La^3+^ (80 μM)	5	0.55 ± 0.11
hTRPC5	La^3+^ (80 μM)	5	0.52 ± 0.11
rTRPC5	La^3+^ (80 μM)	5	0.32 ± 0.05
mTRPC5	20 μM carbachol/M1R	13	~3.4 ±0.6*
hTRPC5	20 μM carbachol/M1R	5	2.0 ± 1.5
mTRPC4	10 μM carbachol/M2R	7	~1.8 ±0.2*
hTRPC4	10 μM carbachol/M2R	5	0.49 ± 0.10**
hTRPC1/hTRPC5	La^3+^ (80 μM)	5	1.4 ± 0.6
hTRPC1/hTRPC5	20 μM carbachol/M1R	5	4.4 ± 1.0
hTRPC1/hTRPC4	10 μM carbachol/M2R	5	1.3 ± 0.4

Listed IC_50_ values are mean ± S.D.

*—The IC_50_ was calculated from percent inhibition values combined from multiple, different cells, and then fitted with the Hill equation (see Methods); the listed S.D. was determined from the curve fitting.

**—IC_50_ values were determined in cells with internal solutions containing either 0 or 4 mM MgATP (block was very similar in these conditions).

To determine whether HC-070 could also block heteromeric channels, we assessed inhibition when TRPC1 was coexpressed with TRPC4 or TRPC5. Currents arising from TRPC1/TRPC5 heteromers were distinguished by their distinct current-voltage relationship, which lacks the dual-rectification characteristic of TRPC5 homomultimers [5]. HC-070 blocked M2R-activated human TRPC1/TRPC4 channels with an IC_50_ of 1.3 nM and La^3+^- and M1R-activated human TRPC1/5 channels with an IC_50_s of 1.4 nM and 4.4 nM, respectively (Table 1). We did not test TRPC1 expressed on its own, as we do not observe a current when the clone is transfected independently of other TRPC family members, consistent with previous reports [5, 18, 19]. Whether HC-070 would inhibit TRPC1 when heteromultimerized with other channels is not known. Overall, these IC_50_ values are similar to those observed with HC-608 under identical testing conditions (Table 1, S1 Table).

**Fig 2:**
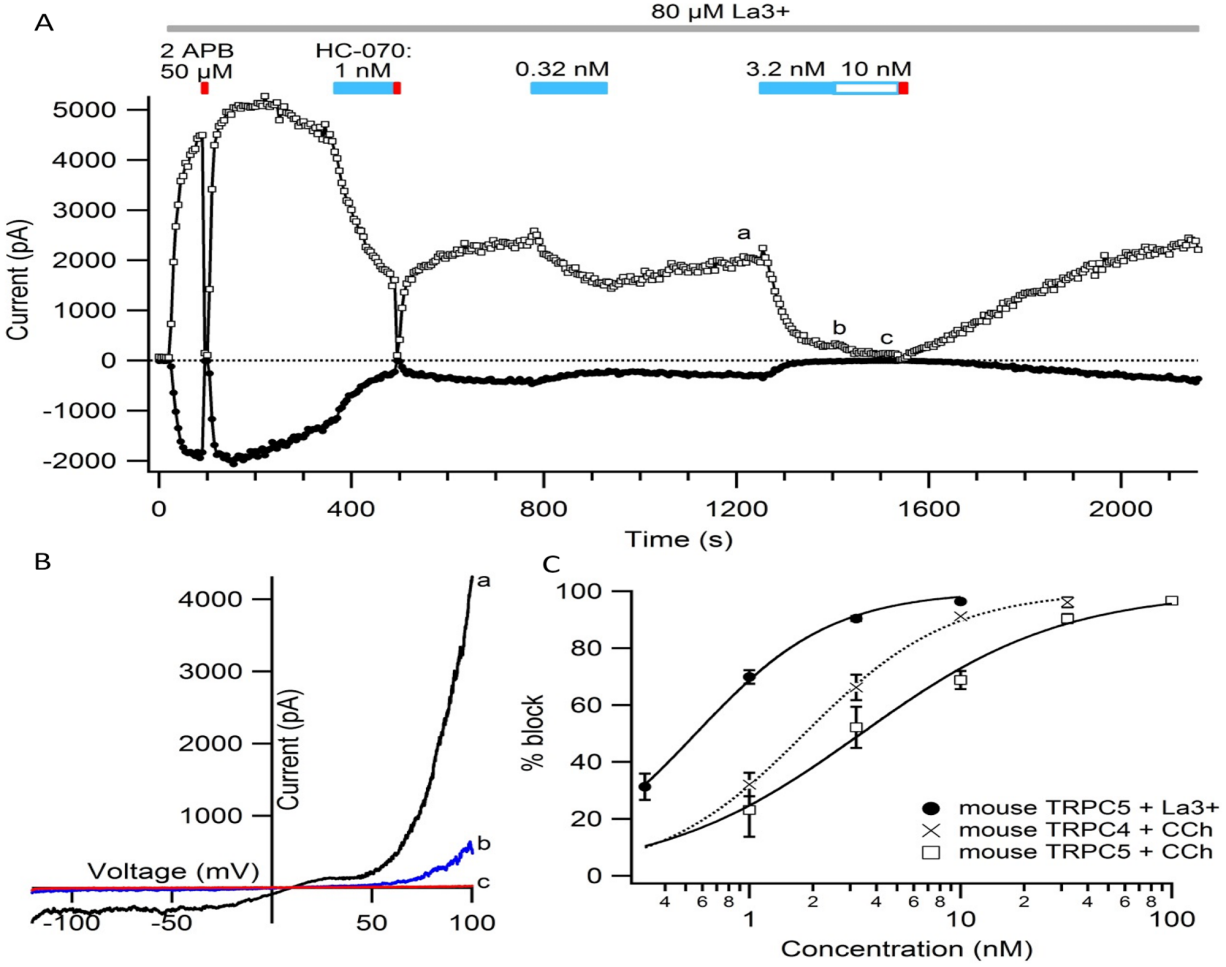
HC-070 inhibition of heterologously expressed mouse TRPC4 and TRPC5 channels. (A) Average currents at -80 mV (filled circles) and +80 mV (open squares) during the application of varying concentrations of HC-070 (blue bars). The TRPC5 current was activated by application of 80 μM lanthanum chloride (LaCl_3_) (gray bar), and 50 μM 2-aminoethyl-diphenyl-borinate (2-APB) (red bar) was used to completely block the TRPC5 current. Dotted lines depict the estimated unblocked current, to account for channel rundown (see Methods). (B) TRPC5 current-voltage curves recorded at the times in A shown by lower case letters (a, b, c). The steps at ±80 mV flanking the ramp, used to calculate the averages in A, are not shown. (C) Dose-response curves of TRPC4 and TRPC5 channel inhibition by HC-070. Symbols are the average % inhibition recorded from 3–13 cells; error bars are the standard error of the mean (SEM; in some cases smaller than the symbol). Solid lines are fits of the Hill equation to the points. Mouse TRPC5 channels activated by 80 μM LaCl_3_ are shown by filled circles (fit parameters IC_50_ = 0.52 nM, Hill coefficient = 1.31). Mouse TRPC4 (mTRPC4) channels activated by carbachol stimulation of human muscarinic 2 receptor (M2R) are shown in crosses (fit parameters IC_50_ = 1.8 nM, Hill coefficient = 1.27), and mTRPC5 channels activated by carbachol stimulation of the human muscarinic type 1 (M1R) is shown in open squares (fit parameters IC_50_ = 3.4 nM, Hill coefficient = 0.91).

HC-070 also reversibly blocked lanthanum-activated mouse TRPC5 (mTRPC5) current (Fig 2 panels A and B) with an IC_50_ of 0.55 nM (Fig 2 panel C and Table 1). A representative current-voltage relationship in the presence and absence of blocker is shown in Fig 2 panel B. The slightly greater potency of HC-070 block of La^3+^-activated versus carbachol-activated TRPC5 channels might result from different extents of channel activation caused by the two stimuli we used, or differential allosteric coupling between activation mechanisms and compound binding site, among other possibilities. Similar dependence of antagonist potency on the method of activation has been reported for other TRP channels [20, 21]. Future work will be needed to detail how HC-070 inhibits TRPC5 channels.

Since *in vivo* TRPC currents are likely to be activated by receptor stimulation, we assessed the ability of HC-070 to affect currents elicited by carbachol stimulation of the muscarinic receptors. HC-070 inhibited human TRPC5 currents activated via M1R with an IC_50_ of 2.0 nM. hTRPC4 currents were activated by the M2R, because M2 produced a more robust current than other receptors tested. HC-070 inhibited hTRPC4 currents with an IC_50_ of 0.49 nM (Table 1). Similar values were observed with HC-608 (S1 Table). Despite variable run-down across cells and conditions of activation, we saw very little impact on our IC_50_s with standard deviations for all conditions tested less than 35%.

Because of our interest in conducting behavioral pharmacology experiments in mice, we also assessed the potency of HC-070 on the murine versions of TRPC4 and TRPC5 when activated by carbachol. Given the high homology of the mouse and human channels, as expected, HC-070 was also highly potent relative to the orthologs tested (Fig 2 panel C and Table 1). These results indicate that HC-070 and HC-608 potently inhibit TRPC4 and TRPC5 currents *in vitro*.

A useful inhibitor requires high selectivity in addition to high potency. We first measured the maximal solubility of HC-070 in Ringer’s solution using high performance liquid chromatography (HPLC). The resulting value was 1.9 μM. Therefore, we used this as the maximal concentration tested in our in vitro patch clamp assays assessing selectivity. We investigated the ability of HC-070 and HC-608 to inhibit the activity of a range of ion channels in whole-cell manual patch clamp, representing some of the major families. These included: TRPC3, TRPC6, transient receptor potential cation channel subfamily vanilloid member 1 (TRPV1), TRPV member 3 (TRPV3), transient receptor potential cation channel subfamily ankyrin member 1 (TRPA1), transient receptor potential cation channel subfamily melastatin member 8 (TRPM8), potassium voltage-gated channel subfamily A member 3 (K_v_1.3), potassium voltage-gated channel subfamily A member 5 (K_v_1.5), potassium voltage-gated channel subfamily D member 3 (K_v_4.3), potassium voltage-gated channel subfamily Q member 1 (K_v_7.1), L-type voltage gated calcium channel (Ca_v_1.2), sodium channel, voltage-gated, type II (Na_v_1.2), sodium channel, voltage-gated type 9 (Na_v_1.7), sodium channel, voltage-gated, type 5 (Na_v_1.5) and potassium voltage-gated channel subfamily H member 2 (hERG). In most cases, HC-070 at 1.9 μM inhibited 50% or less, though TRPC3 was inhibited with an IC_50_ of 1.0 μM (S2 Table). This indicates that HC-070 is at least 400-fold selective for human TRPC4 and TRPC5-containing channels compared to the other channels examined. HC-608 showed a similar selectivity profile (S2 Table).

To probe the effects of HC-070 on other targets that might interfere with the interpretation of in vivo efficacy experiments, we assessed its effects at 1 μM on a selection of physiologically relevant receptors, enzymes, kinases, and transporters (see S3 Table, S1 Report). HC-070 showed less than 50% inhibition in all of the targets assayed, indicating the selectivity is at least 400-fold for human TRPC4 and TRPC5. HC-608 was similarly selective (S4 Table, S1 Report).

Although both compounds performed similarly in our in vitro characterization, we decided to focus on HC-070 for subsequent in-depth investigation.

### HC-070 reduces activity in the basolateral amygdala

CCK-4 administration causes increased feelings of fear and anxiety in humans and induces behaviors indicative of fear and anxiety in rodents. Human functional magnetic resonance imaging studies show the emotions induced by CCK-4 correlate with increases in activity in the cortical and limbic brain regions including the amygdala [22], a brain region shown to be overactive in patients with anxiety and mood disorders associated with negative bias (see Discussion) [23–25]. Previous work has shown strong expression of TRPC4 and TRPC5 in the basolateral amygdala and TRPC4 or TRPC5 null mice have reduced CCK-4 receptor-activated slow, inward currents [7, 8].

**Fig 3:**
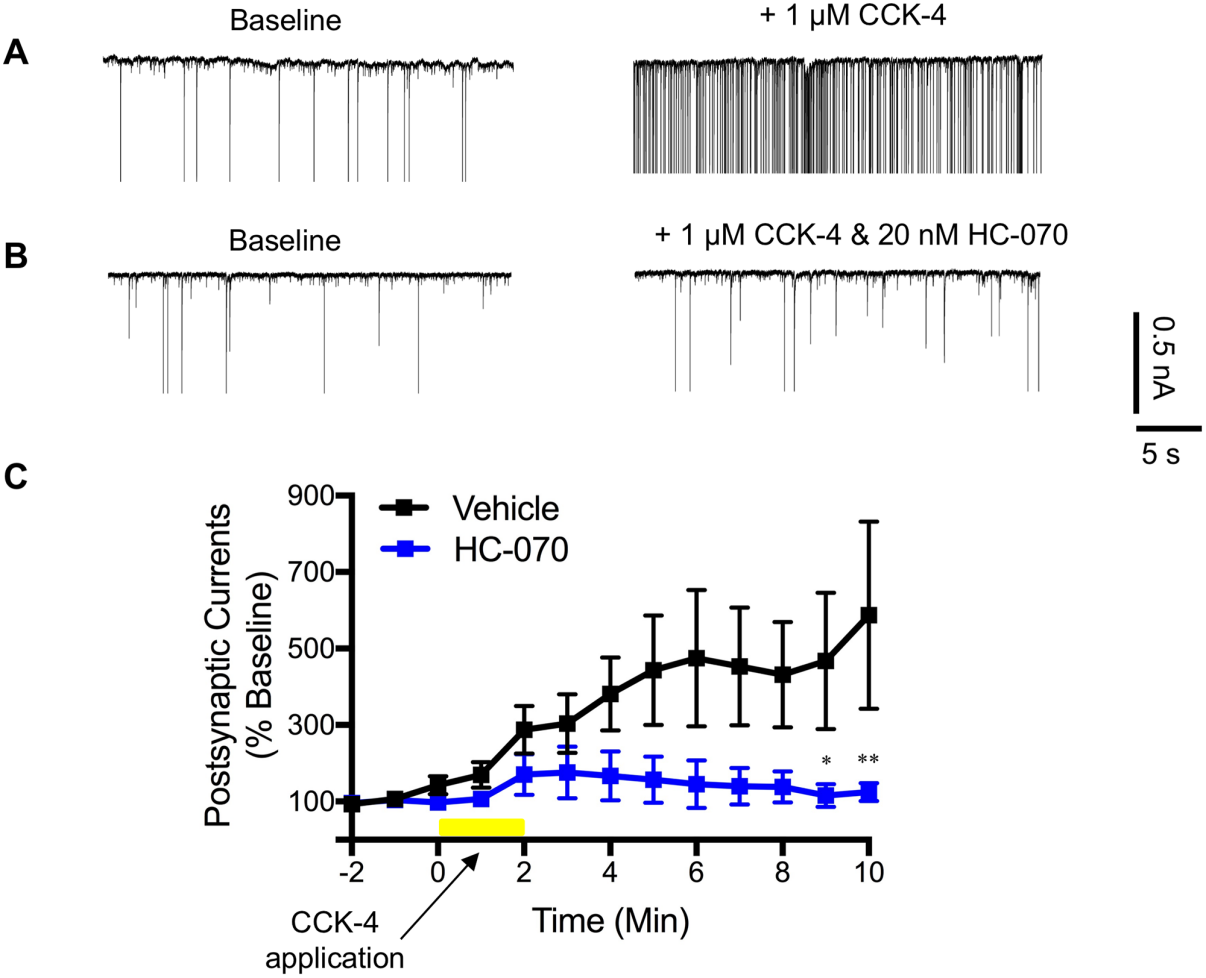
HC-070 attenuates CCK-4 induced EPSCs recorded from the basolateral amygdala in a slice preparation. (A) Representative traces before and after CCK-4 application in the presence of vehicle. (B) Representative traces before and after CCK-4 application in the presence or absence of HC-070. (C) Quantitation of the results (n = 8). HC-070 was pre-incubated with the slice. CCK-4 was applied at the time indicated. Error bars represent the SEM (* p < 0.05, ***p <0.001, Tukey’s multiple comparisons test following two-way analysis of variance (ANOVA)). The holding potential was -70 mV.

We tested whether HC-070 application would prevent or reduce CCK-4 stimulated increases in excitatory post synaptic current (EPSC) frequency recorded from basolateral amygdala neurons (Fig 3). EPSCs were measured by whole-cell recordings made from basolateral amygdala neurons in coronal slices cut from C57BL/6 mouse brains.

Slices were superfused with either 20 nM HC-070 (confirmed by LC/MS) or DMSO vehicle for 5 min. CCK-4 (1 μM) was then applied in the continued presence of HC-070 or vehicle. The number of spontaneous EPSC events (>50pA) was recorded for 10 minutes after CCK-4 addition, and is shown relative to the preceding 2 minute baseline (Fig 3 panel C). The basal synaptic activity prior to CCK-4 addition measured from slices pretreated with HC-070 was 154±35 postsynaptic currents/minute (n = 8) whereas in vehicle treated slices it was 77±18 postsynaptic currents/minute (n = 8).

CCK-4 (1 μM) substantially increased the mean number of postsynaptic currents recorded compared to slices treated with vehicle (Fig 3 panel A), although the variability was high. Application of 20 nM HC-070 led to pronounced attenuation of the CCK-4 related effect (Fig 3 panel B, quantified in Fig 3 panel C). While these EPSCs that we record are distinct from the slow, excitatory currents previously reported [7,8], they are indicative of changes in excitability resulting from increased presynaptic activity from neurons projecting to the recorded neuron. Although we did not directly test the effect of HC-070 on native TRPC4 or TRPC5 channels in neurons, our results show that treatment with HC-070 reduces CCK-4 evoked neuronal activity in the amygdala slices, by impacting the presynaptic inputs to the region.

As expected given the high nonspecific tissue binding of HC-070 (>99%; see section on Pharmacokinetic properties) we found higher compound concentrations in the brain slices (973 ± 173 nM, n = 9) compared to the concentration in the buffer. The buffer concentration of 20 nM, which we assume to reflect the free concentration of compound, should largely block TRPC4- and TRPC5-containing channels. Given the observed selectivity profile, it is likely that the observed compound effects at this concentration are TRPC4 and TRPC5-dependent.

### Pharmacokinetic properties

**Fig 4:**
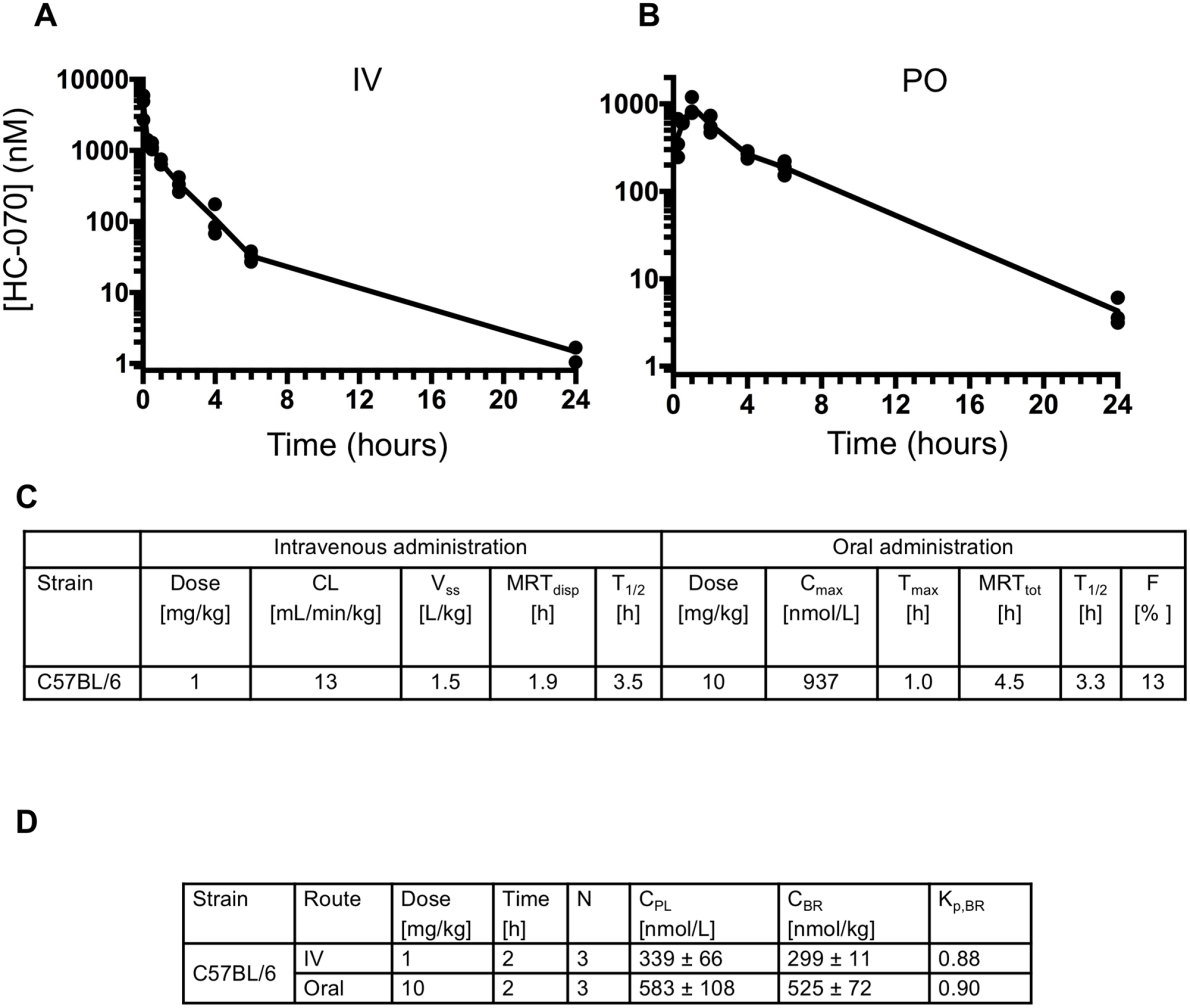
Pharmacokinetic properties of HC-070. PK profiles of HC-070 after intravenous (A) and oral (B) administration in C57BL/6 mice. Plasma concentrations were determined by LC-MS/MS. Points represent the individual concentrations at the times indicated. Lines represent mean exposure (n = 12 mice/arm, n = 3 data points per time point). (C) Summary of PK properties. CL = clearance; V_ss_ = volume of distribution at steady state; MRT_disp_ = mean residence time of drug molecules after intravascular administration; T_1/2_ = half-life. (D) Plasma and brain concentrations measured 2 hours after intravenous or oral administration of 1 or 10 mg/kg HC-070, respectively. C_PL_ = concentration in plasma, C_BR_ = concentration in brain, K_P,BR_ = partitioning coefficient between brain and plasma.

Before testing HC-070 in behavioral assays, we sought to understand the PK properties of the compound in the C57BL/6 mouse. For intravenous (IV) administration, HC-070 was formulated as a solution at a final dose of 1 mg/kg. Plasma concentrations at various time points were determined using liquid chromatography tandem mass spectroscopy (LC-MS/MS) technique (Fig 4 panel A). To assess if there were mouse strain differences, we repeated the pharmacokinetic experiments in Swiss Webster mice (S2 Fig); the concentration-time profiles of the two strains were quite similar.

The elimination half-life of HC-070 was 3.5 hours and 3.6 hours in C57BL/6 and Swiss Webster mice, respectively. We observed a low clearance of 13 and 11 mL/min/kg for the two strains, respectively. The volume of distribution at steady state (V_ss_) for HC-070 was 1.5 and 2.2 L/kg in C57BL/6 and Swiss Webster mice, respectively. This pharmacokinetic parameter is the apparent volume of plasma required to account for all the drug in the body. A value larger than the volume of body water (ca. 0.7 L/kg), as measured for HC-070, indicates that the compound distributes readily into tissue.

For oral gavage dosing, HC-070 was formulated as a simple suspension in 0.5% methyl cellulose. The oral dose of 10 mg/kg achieved its maximal plasma concentration at 1 hour in both strains, indicating relatively rapid absorption (Fig 4 panel B and S2 Fig panel B). The elimination half-life of the compound was 3.3 and 2.7 hours in non-fasted C57BL/6 and Swiss Webster mice, respectively. Comparing the HC-070 area under the curve when administered orally versus intravenously, the oral bioavailability was between 13 and 23%. The PK properties in the two strains are summarized in Fig 4 panel C and S2 Fig panel C.

Since the properties observed were so similar between the two strains of mice tested, we did not perform full pharmacokinetic studies in the other mouse strains used. Rather, we took plasma levels from animals at the end of the study where possible and compared them with the expected values.

Because the proposed site of action for a TRPC4/5 antagonist is the brain, we compared the compound levels in brain with those found in plasma two hours after dosing. Following IV (1 mg/kg) administration, 339 ± 66 nmol/L HC-070 were present in plasma while 299 ± 11 nmol/kg compound were detectable in brain. Brain and plasma exposures, after a 10 mg/kg po dose were similar after oral administration, namely 583 ± 108 nmol/L plasma and 525 ± 72 nmol/kg brain tissue (summarized Fig 4 panel D). Due to the high PPB of HC-070 (99.6%) we also measured the unbound fraction in brain; this was 0.0028 ± 0.0002 at a HC-070 concentration of 1 μM. While the above measured total brain concentration of 525 nmol/kg is high, the pharmacologically-relevant, unbound brain concentrations was only 1.5 nmol/kg. These observations confirmed that HC-070 has ready access to the brain and guided dose selection for in vivo testing in behavioral models, allowing focus on levels that should yield unbound brain exposure within 10-fold of the unbound IC_50_ for TRPC4 and TRPC5.

### In vivo efficacy in tests and models of anxiety and fear

#### Elevated plus maze

**Fig 5:**
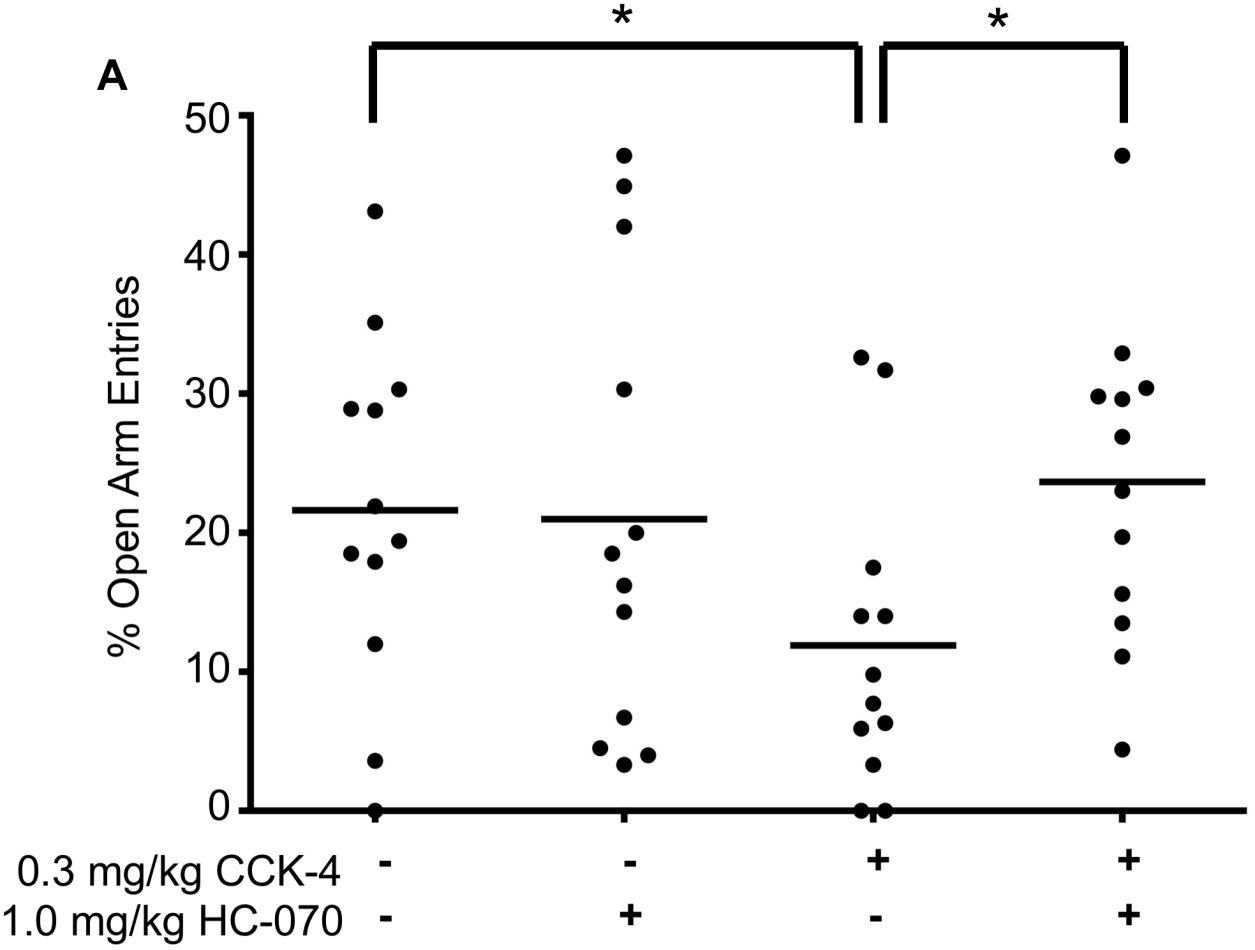
HC-070 decreases anxiety after CCK-4 treatment in an EPM. Treatment with CCK-4 decreased the percent of open arm entries (p < 0.05, t-test), as expected. Administering HC-070 orally at 1 mg/kg prevented the reduction in open arm entries induced by CCK-4 (p<0.05, t-test, n = 12/group). HC-070 exerted no effect on percent of open arm entries in the absence of CCK-4. Each animal is represented on the graph (circles) and the horizontal lines represent the mean. The actual exposures of HC-070 at the time the mice were sacrificed were 262±63 and 301±81nM for the CCK-4 + HC-070 and VEH + HC-070 groups, respectively.

The EPM test is an in vivo test widely used to assess anxiolytic activity. In this test, a conflict (the state of anxiety) is established between the animal’s innate motivations to seek safety (closed arms) and to explore a relatively unsafe environment (open arms). Anxiolytic manipulations reduce the motivation to seek safety, thus increasing entry into, and time spent, on the open arms. Anxiogenic manipulations decrease open arm entries. One such anxiogenic manipulation is activation of the CCKB receptor [26]. Since HC-070 dramatically reduces CCK-4 dependent synaptic activity in brain slices, we tested the effects of the compound on CCK-4 induced anxiety in the mouse. Conditions were modified from the standard EPM test by reducing light intensity in order to reduce basal anxiety, thus allowing detection of a CCK-4 anxiogenic effect. Systemic administration of 0.3 mg/kg (intraperitoneal, IP) CCK-4 decreased the percent of entries into the open arms as expected [26]. This effect was completely blocked by oral administration of HC-070 at 1 mg/kg (Fig 5). HC-070 only affected mice with increased evoked anxiety (CCK-4). Animals treated with HC-070 in the absence of CCK-4 showed no changes in anxiety under these low anxiety conditions. Average HC-070 plasma levels were in line with expected values.

**Fig 6:**
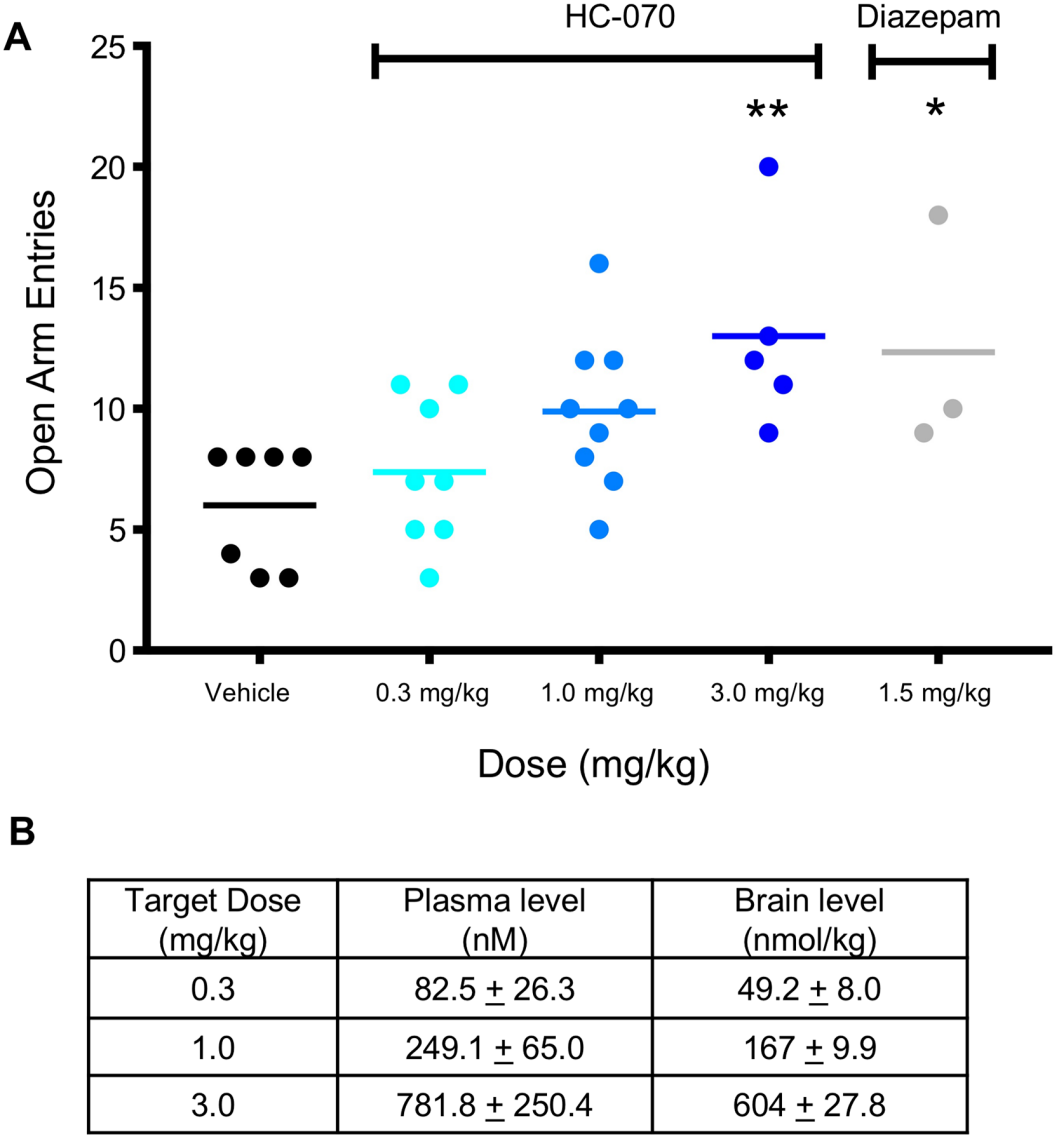
HC-070 decreases anxiety in a standard EPM. (A) Mice were administered vehicle or 0.3, 1 or 3 mg/kg HC-070 orally 60 minutes prior to EPM. The positive control, 1.5 mg/kg diazepam, was administered IP 30 minutes prior to testing. At 3 mg/kg, HC-070 significantly increased the number of open arm entries compared to the oral control (** p<0.01, Dunnett’s post-hoc test following one-way ANOVA). The positive control, 1.5 mg/kg diazepam, also increased open arm entries (* p <0.05, t-test). Animals that jumped or fell off the EPM during the test were excluded, such that the sample size (n) differed between groups. Each animal is shown on the graph (circles) and the horizontal lines represent the mean. (B) Average plasma and brain exposures from satellite animals dosed with 0.3, 1, or 3 mg/kg HC-070, 60 minutes post dosing. Error bars show standard deviation.

To test the impact of HC-070 in a standard EPM (more light/high anxiety), the number of open arm entries was recorded for 5 minutes, 1 hour post oral administration of vehicle or 0.3, 1 or 3 mg/kg HC-070, or 30 minutes post IP administration of 1.5 mg/kg diazepam, the positive control. Administration of HC-070 increased open arm entries compared to vehicle in a dose dependent manner with the 3 mg/kg dose achieving significance (Fig 6 panel A, p <0.01, Dunnett’s post-hoc test for significance following one-way ANOVA), consistent with previous reports from TRPC4 and TRPC5 null mice [7, 8]. The anxiolytic diazepam significantly increased the total number of open arm entries to a similar degree as 3 mg/kg HC-070 (Fig 6 panel A, p <0.01, t-test). The sample size of animals per group was uneven because the small number of mice that jumped or fell off the EPM during the experiment were excluded. Compound exposures were again found to be in the expected range (Fig 6 panel B). The unbound brain concentration at 3 mg/kg, calculated from the total brain concentrations and the fraction unbound in brain, was 1.7 nM.

#### Chronic social defeat and fear learning and memory

Uncontrollable stressful events are known to be etiological factors leading to generalized changes in emotion and increased responsiveness to neutral stimuli predicting aversive stimuli in patients. This can be tested in a model of chronic social stress-induced fear hyperreactivity in mice, where fear was measured using Pavlovian conditioning [27].

Firstly, we studied HC-070 effects in the fear learning and memory test in otherwise non-manipulated mice (S3 Fig). This test comprises placing the animal in an arena (context) and pairing a tone, the conditioned stimulus (CS), with mild electroshocks, the unconditioned stimulus (US), and measuring fear “freezing” (complete immobility) to the CS. On the first test day baseline freezing levels were measured by placing mice in the arena without CS or US, and without drug; all mice showed low baseline freezing (S3 Fig panel A). On the next day, mice were administered vehicle or HC-070 at 1 or 3 mg/kg orally and 2 hours later underwent CS-US fear conditioning. The compound did not affect fear learning, with each group acquiring increased freezing to the CS across successive CS-US trials (Trial-block main effect p<0.0005, S3 Fig panel B). On the next day, mice were administered HC-070 at the same dose and after 2 hours were placed back in the same context in which conditioning took place. There was no drug effect on fear memory expression to the context (p = 0.82 S3 Fig panel C). Immediately thereafter, the CS was presented repeatedly: there was no drug effect on fear memory expression to the CS (p = 0.27 S3 Fig panel D), with mice in each group showing a high and similar level of freezing to the first CS followed by a consistent decrease across CS trials (p<0.0005).

**Fig 7:**
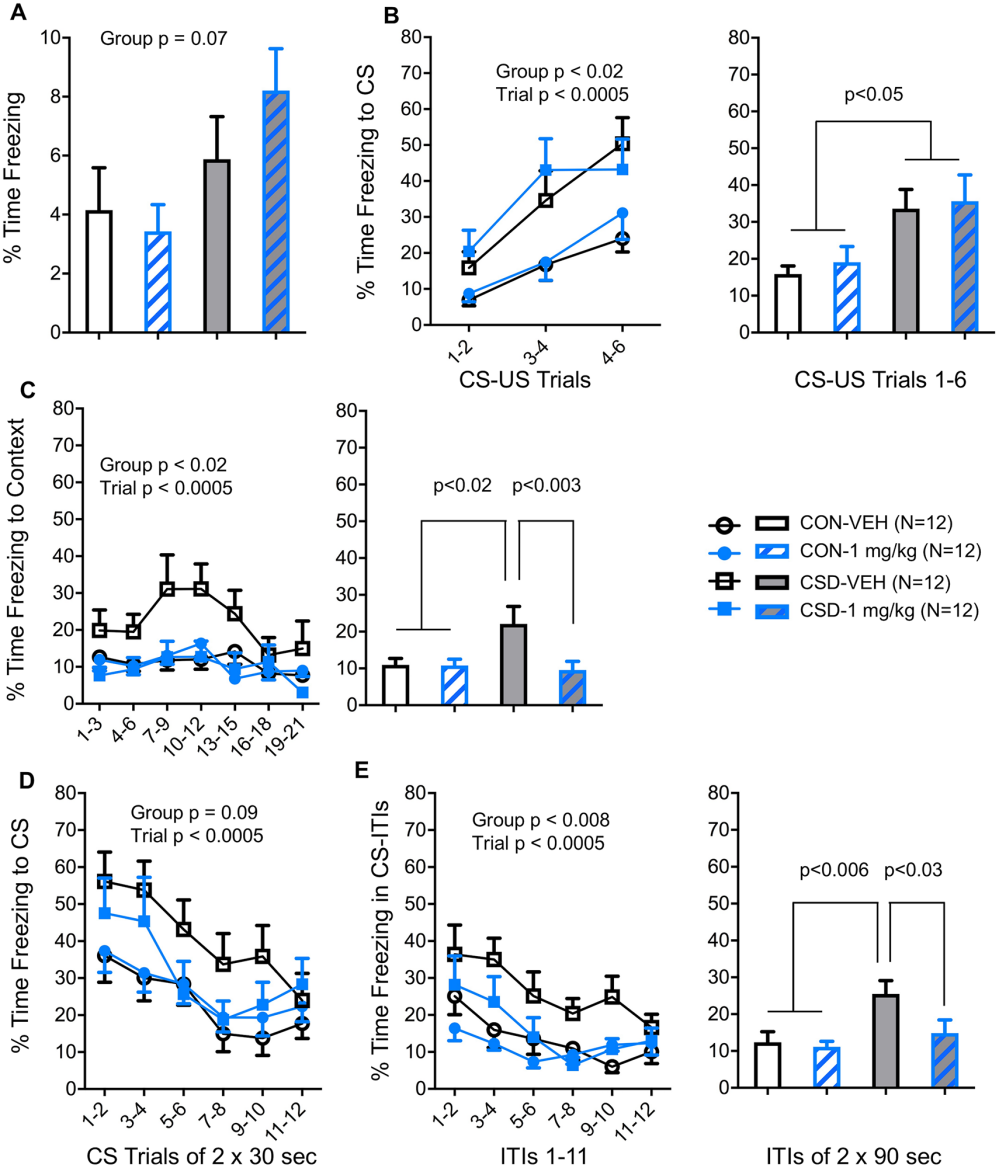
Effects of HC-070 on CSD-induced fear hyper-reactivity. HC-070 reduces the increased capacity for fear memory in mice exposed to chronic social stress on days 1–15. (A) Day 16: Without drug administration, when placed in a relatively unfamiliar arena (context) without tone or electroshock, mice that had been exposed to chronic social defeat (CSD) tended to show more freezing—a fear behavior—than did control mice (CON). (B) Day 17: Without drug administration, mice were placed back in the same context and exposed to 6 pairings of a 20 s tone conditioned stimulus (CS) that announced a 2 s electroshock unconditioned stimulus (US). CSD mice acquired more freezing to the CS than did CON mice. The left panel presents the fear learning curve using the average freezing during CS-US trials 1–2, 3–4 and 5–6, and the right panel presents the average freezing across all six CS-US trials. Immediately after this CS-US conditioning session, CSD and CON mice received either vehicle or 1 mg/kg HC-070 orally. (C) Day 18: Mice received vehicle or 1 mg/kg HC-070 orally and 1 hour later were placed in the same context in which CS-US conditioning took place the previous day, and a 21-min test of context fear memory was conducted, i.e. in the absence of the CS (and US). The left panel presents the context-fear expression curve using the average freezing per 3-min block. The right panel presents the average context-fear expression across all 21 min. HC-070 significantly reduced the context fear memory in CSD mice, as indicated by these mice showing freezing levels similar to those of CON mice and lower than those of CSD-VEH mice. (D) Day 18: Immediately after the context fear memory test, the tone-CS fear memory test was conducted, comprising 12 trials of 30-s CS separated by 90-s inter-trial intervals. The fear expression curve to the CS is presented using the average freezing per pair of consecutive CS trials. HC-070 tended to reduce the CS fear memory in CSD mice, as indicated by the increased rate at which their freezing level during the CS attenuated compared with CSD-VEH mice, i.e. faster extinction learning. (E) Day 18: In the tone CS memory test presented in (D), freezing was also measured in the inter-trial intervals (ITIs) between CS presentations. The left panel presents the fear expression curve in ITIs between CSs using the average freezing per pair of consecutive ITIs. The right panel presents the average freezing across all 12 ITIs. Here also, HC-070 reduced fear memory in CSD mice, as indicated by the increased rate at which their freezing level during the ITIs attenuated compared with CSD-VEH mice, i.e. faster extinction learning.

Chronic social defeat (CSD) involves exposing mice to prolonged social stress, and increases fear learning and memory [28]. In this paradigm, a smaller (CSD) C57BL/6 mouse is housed next to a larger, dominant CD-1 mouse, separated by a Plexiglas barrier permeable to sensory cues. Once daily for 15 days, for a brief period of time the submissive mouse is introduced to the aggressor mouse. Following this period of social stress, the CSD mice and control mice were studied in the fear learning and memory test, in which time spent freezing provides a measure of fear learning and subsequent fear memory. On day 16, the mice were placed in the arena to test baseline freezing levels, and there was a trend to increased freezing in CSD compared with control mice (Fig 7 panel A). On day 17, mice underwent CS-US fear conditioning; relative to controls, CSD mice showed increased CS-US fear learning (Fig 7 panel B), as expected based on previous studies [28]. Immediately after this experience, both CSD and control mice were administered vehicle or HC-070 at 1 mg/kg PO. The next day, mice received the same dose of HC-070 (vehicle or 1 mg/kg) and 1 hour later were placed back in the arena. Firstly, mice underwent a test of context fear memory, that is, they were placed in the arena without the CS or US. TRPC4/5 inhibition reduced the relatively high context fear memory in CSD mice (Fig 7 panel C). Second, mice underwent a test of CS memory, with the CS presented during repeated trials and separated by inter-trial intervals. TRPC4/5 inhibition reduced the relatively high CS fear memory in CSD mice, with the duration of freezing decreasing more substantially across successive CS presentations in CSD-HC-070 mice than in CSD-vehicle mice (Fig 7 panel D). Also in the intervals between CS presentations, CSD-vehicle mice showed high levels of freezing whereas freezing in CSD-HC-070 mice was ameliorated to the level of control mice (Fig 7 panel E). These results indicate that TRPC4/5 inhibition acts to reverse the CSD stress effect of increased consolidation/expression of the fear memory of the discrete CS and the context. The time course of the effects suggests that the primary effect of TRPC4/5 inhibition is to increase the extinction of these fear memories (Fig 7 panels D-E).

#### Marble burying test

In addition to using bedding material to bury noxious materials, rodents also bury harmless objects such as rat chow pellets and glass marbles. It has been proposed that marble burying shares features of compulsive behavior [29]. Selective serotonin-reuptake inhibitors, which are used clinically in the treatment of patients with obsessive compulsive disorder, reduce the number of marbles buried [29]. Because there are features of anxiety in this model, we probed the effect of inhibiting TRPC4 and TRPC5.

**Fig 8:**
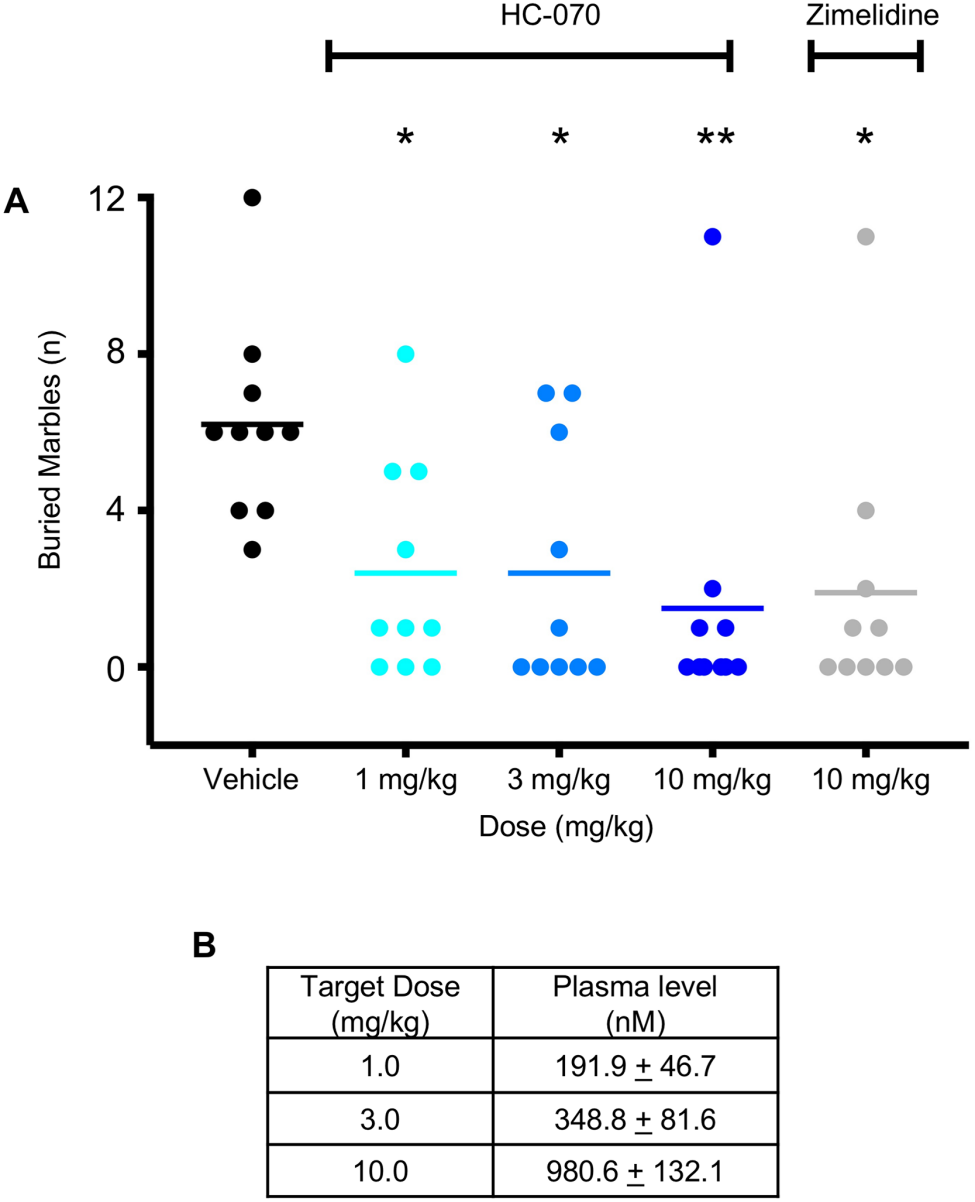
HC-070 reduces marble burying behavior. (A) Mice were administered vehicle or 1, 3 or 10 mg/kg HC-070 orally, 60 minutes prior to testing. The positive control, 10 mg/kg zimelidine, was administered IP 45 minutes prior to testing. At 1, 3, and 10 mg/kg, HC-070 significantly decreased the number of buried marbles compared to vehicle (p <0.05 *, p<0.01**, Dunnett’s post-hoc test following one-way ANOVA, n = 10/group). Zimelidine also decreased the number of buried marbles (p < 0.05, t-test, n = 10/group). Each animal is represented on the graph and the horizontal lines represent the mean. (B) Average plasma exposures from animals that completed the test. Error bars are the standard deviation.

We treated female C57BL/6 mice with vehicle or 1, 3 or 10 mg/kg HC-070 PO 60 minutes prior to the marble burying test. The positive control, 10 mg/kg zimelidine, was administered IP 45 minutes prior to testing. The number of buried marbles was recorded after 30 minutes by an observer blind to compound treatment. At each dose, HC-070 decreased the total number of marbles buried by more than 50% compared to vehicle (Fig 8 panel A, p <0.05, Dunnett’s post-hoc test for significance following one-way ANOVA). Zimelidine also significantly reduced the total number of marbles buried, as expected (Fig 8 panel A, p <0.05, t-test). Compound exposures were consistent with other studies (Fig 8 panel B).

One interesting feature of this test that distinguishes it from others is that efficacy is based on the animal decreasing its activity i.e. reduced marble burying. This is distinct from tests of anxiety such as the EPM and tests of anti-depressant activity such as the forced swim test where an increase in activity provides the positive readout.

### In vivo efficacy in tests of anti-depressant activity

**Fig 9:**
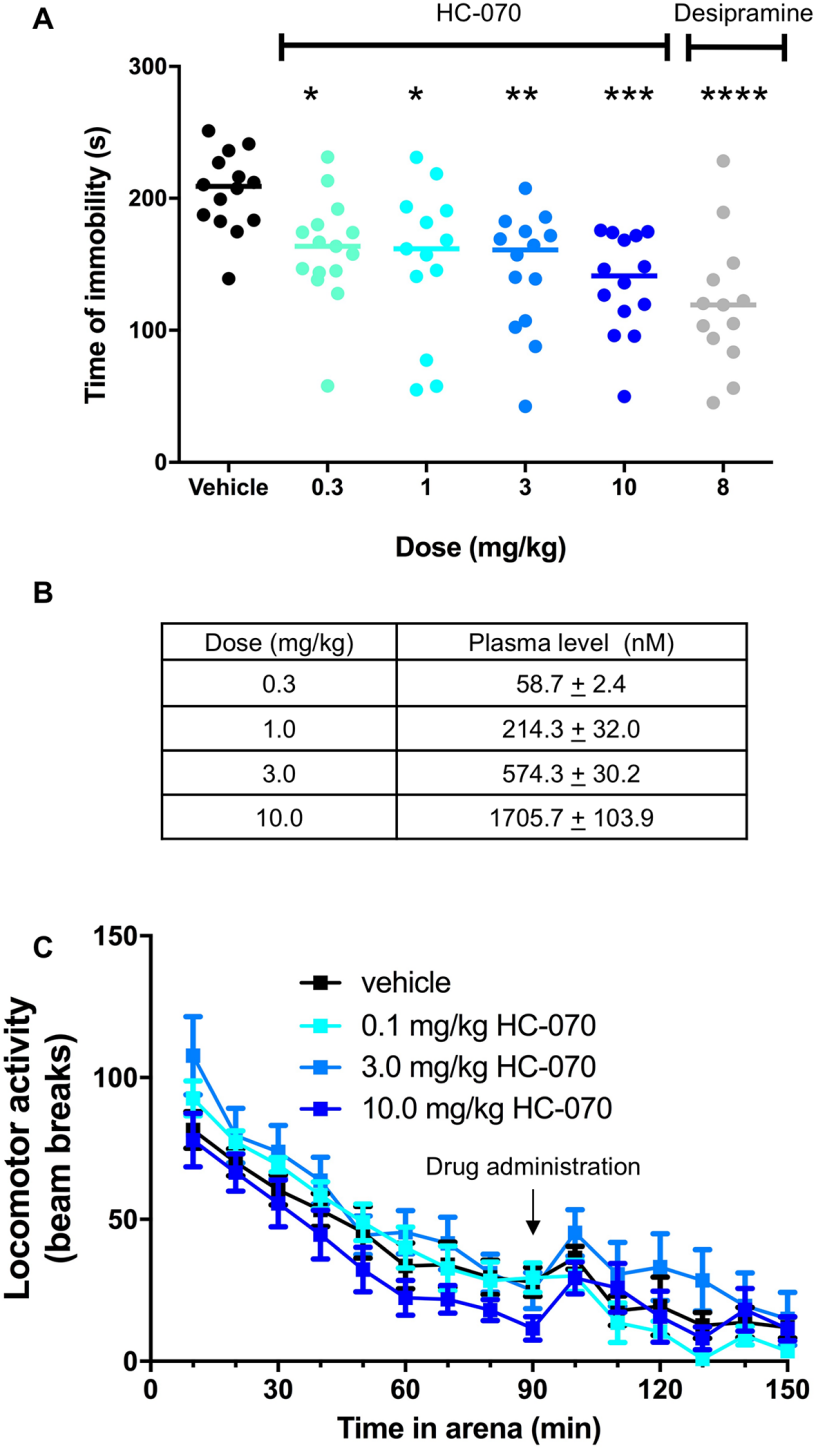
HC-070 reduces time of immobility in a tail suspension test but does not impact locomotor activity. (A) Mice were administered vehicle or 0.3, 1, 3 or 10 mg/kg HC-070 PO 60 minutes prior to testing. The positive control, 8 mg/kg desipramine, was also administered orally. All doses of HC-070 reduced the time of immobility (p <0.05 *, p<0.01**, p < 0.001 *** Dunnett’s post-hoc test following one-way ANOVA, n = 13–16 group), as did desipramine (p <0.0001, t-test) Each animal is shown on the graph and the horizontal lines represent the mean. (B) Associated exposures. Error bars are the standard deviation. (C) Locomotor activity was not impacted. After 90 minutes of habituation to an activity chamber, mice were administered vehicle or 0.1, 3 or 10 mg/kg HC-070 PO. Activity was recorded for another 60 minutes. There was no effect of dose (2-way ANOVA followed by a Tukey’s multiple comparison’s test, p>0.5 for all treatments; n = 8/group).

HC-070 also demonstrated significant effects in tests known to be sensitive to anti-depressants. One commonly used test is the tail suspension test. NMRI mice are suspended by their tails and the amount of time they are active versus remaining immobile is recorded. HC-070 was delivered orally at 0.3, 1.0, 3.0 or 10 mg/kg 60 minutes prior to assessment. Compound treatment reduced the amount of time animals were immobile at all doses (Fig 9 panel A, p <0.05, Dunnett’s post-hoc test for significance following one-way ANOVA). The effect size was similar to that observed with the positive control, the tricyclic anti-depressant, desipramine (Fig 9 panel A, p <0.05, t-test). Plasma levels were as expected in test animals (Fig 9 panel B).

Locomotor activity in transparent square cages equipped with 2 light barrier systems was not impacted by these doses of HC-070 (two-way ANOVA, followed by a Tukey’s multiple comparisons test; p>0.5) (Fig 9 panel C), suggesting that the effects observed were specific to an acutely stressful situation. Phencyclidine (PCP), the positive control in the locomotor activity test, had the anticipated effect of decreasing activity (S4 Fig). Together, these data suggest that HC-070 exerts a behavioral effect similar to that of anti-depressants in in the mouse tail suspension test.

**Fig 10:**
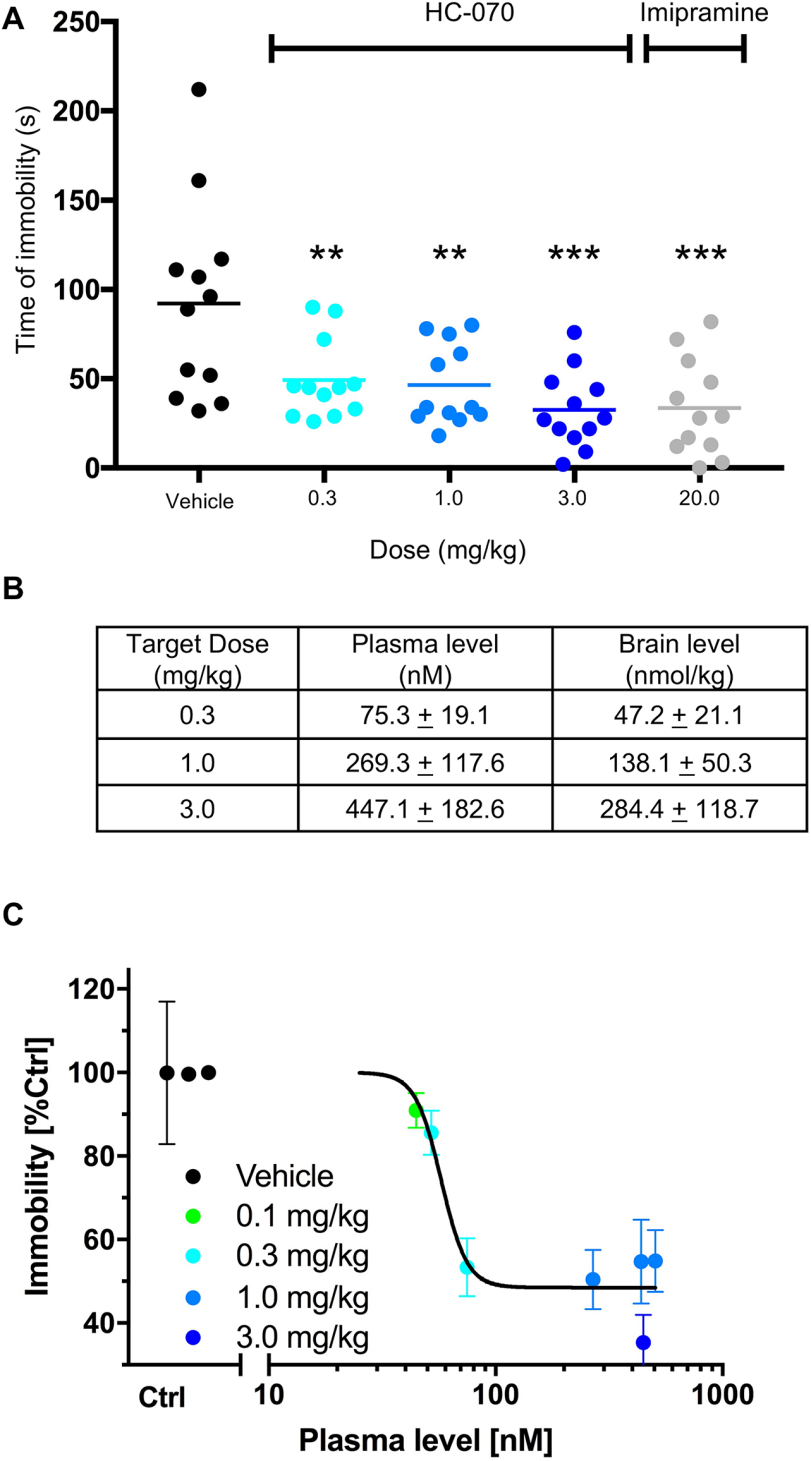
HC-070 reduces time spent immobile in the FST in a dose and exposure-dependent fashion. (A) HC-070 reduces the time spent immobile in the forced swim test to a similar degree as the positive control. Mice were administered vehicle, or 0.3, 1 or 3 mg/kg HC-070 PO, 60 minutes prior to FST. The positive control, imipramine, was administered IP at 20 mg/kg 45 minutes prior to testing. At all doses tested, HC-070 significantly decreased the time mice spent immobile (p<0.01**, p<0.01***, Dunnett’s post-hoc test following one-way ANOVA, n = 12/group). The positive control, 20 mg/kg imipramine, also decreased immobility (p < 0.001, t-test, n = 12/group). (C) PK-PD modelling of the HC-070 concentration-effect from 3 separate forced swim studies. The included studies had the following treatment arms: vehicle, 0.3, 1, 3 mg/kg dose of HC-070 in male CD-1 mice, n = 12 per group (also shown in A); a vehicle and 1 mg/kg dose of HC-070 in male C57BL/6 mice (n = 11 per group); and a vehicle, 0.1, 0.3, 1 mg/kg dose of HC-070 in male C57/BL6 (n = 12 per group). Vehicle and HC-070 were administered PO, 60 min prior to testing. Immobility was normalized to the vehicle control of each individual study. Error bars are SEM.

To extend these findings, we also examined the effects of HC-070 in the forced swim test (FST) in CD-1 mice. In this test, mice are placed in a water-filled cylinder and the amount of time they are mobile (swim, climb) versus the amount of time they are immobile is recorded. The immobility recorded in this test can be reversed by all major classes of antidepressants [30]. HC-070 at doses of 0.3, 1.0 or 3.0 mg/kg 60 minutes prior to testing reduced the immobility time (p <0.05, Dunnett’s post-hoc test for significance following one-way ANOVA, Fig 10 panel A). The positive control, the tricyclic anti-depressant imipramine (20 mg/kg, IP), also decreased immobility time (p < 0.05 t-test, Fig 10 panel A) and to a similar magnitude to that of 3 mg/kg HC-070. Plasma levels of HC-070 were as anticipated based on PK studies performed in other strains of mice (Fig 10 panel B).

Since we could observe graded responses in the forced swim test, we used this paradigm to probe the relationship between pharmacodynamic effects of HC-070 and pharmacokinetics. To ensure the robustness and reproducibility of the response, we tested a second mouse strain at a second site. Results between the two strains and sites were consistent and the efficacious doses were similar, with 1 mg/kg exerting significant efficacy. Plotting the average effect on immobility versus the average plasma exposure resulted in a sigmoidal curve with an IC_50_ corresponding to 58 nM (Fig 10 panel C), establishing a clear relationship between PK and PD in the FST. When corrected for protein binding, this corresponds to an unbound plasma level of 0.23 nM and calculated free brain concentrations of 0.16 nM. These values are lower than the IC_50_ values after receptor activation in the heterologous system for mouse TRPC5 (3.4 nM) and mouse TRPC4 (1.8 nM). Observing biological effects at unbound levels below the IC_50_ is not uncommon for ion channel targets [31–33].

## Discussion

Our results provide strong evidence that acute inhibition of TRPC4 and TRPC5 with HC-070 reduces behavior associated with anxiety and depression in mouse models.

In brain slices, application of HC-070 dramatically reduces EPSC-frequency in the basolateral amygdala stimulated by the panic inducing peptide, CCK-4. Which presynaptic neurons within the network are the source of the observed change in activity was not investigated in our experiments and remains to be determined. It is hypothesized that in patients with anxiety and mood disorders an overactive amygdala is a major contributor to attentional bias to negative stimuli, pessimistic thoughts, and anxiety [23–25]. In their recently published Research Domain Criteria (RDoC) framework, the NIMH suggests that changed cortico-limbic reactivity is a central feature of the symptoms associated with negative emotional processing such as anxiety, rumination, and depressed mood [34]. Normalization of the hyperactive circuits may therefore improve emotional control and reduce these negative valence systems. Behaviorally, HC-070 increases exploratory behaviors in EPM after CCK-4 exposure. It is possible that HC-070 exerts its behavioral effects, at least in part, by reducing excitability of the amygdala. Other brain regions where TRPC4 and TRPC5 are expressed may also be important in mediating the behavioral effects of HC-070.

Pharmacokinetic studies reveal that oral dosing of HC-070 as a simple methylcellulose suspension results in plasma and brain exposures compatible with efficacy testing. HC-070 has a high non-specific binding to tissue, thus total plasma and tissue concentrations are not reflective of the pharmacologically relevant exposure, and unbound plasma and unbound brain concentrations are more relevant for in vivo efficacy. As is observed with many ion channel inhibitors, free concentrations below the IC_50_ already result in efficacy. Overall, the unbound plasma and brain concentration range at which we observe efficacy are within 10-fold of the IC_50_ for TRPC4 and 5 determined in vitro.

In the behaving mouse, inhibition of TRPC4 and TRPC5 results in anxiolytic disinhibition of exploration in a CCK-anxiety model, decreased marble burying, increased activity in tests of antidepressant efficacy, and amelioration of hyper-fear memory in socially stressed mice. The latter effect is largely due to acceleration of memory extinction for the aversive conditioned stimulus by HC-070. As amygdala activity is also a known regulator of emotional processing and stress induced by CSD [27, 28], we speculate that reduced amygdala activity may also underlie some of HC-070 efficacy in the behavioral effects observed.

Consistent with work in null mice [7, 8], inhibition of TRPC4 and TRPC5 did not impact fear learning or fear memory in non-manipulated (control) mice. Blocking the channels also had no generalized effect on locomotion, suggesting that HC-070 is more likely to reduce the negative valence systems than impact arousal or psychomotor function.

Whilst the present data demonstrate that HC-070 is a potent and selective blocker of TRPC4 and TRPC5 and a modulator of neuronal activity and anxious behavior, some questions remain regarding the specific neuropharmacological effects of HC-070. The methods of activation are different for the two channels and multiple pathways are likely to contribute to channel activation *in vivo*. In our studies, we have not investigated whether or not HC-070 preferentially inhibits TRPC4 or TRPC5 or whether it inhibits both equably.

In a native system, we show that HC-070 reduces CCK-4 stimulated activity in the basolateral amygdala, a region of high TRPC4 and TRPC5 expression that is essential in emotional neural circuitry. Nonetheless, future studies will be needed to determine whether additional brain regions are important in mediating the behavioral effects of HC-070. Furthermore, it will be important to address which cells types express TRPC4 and/or TRPC5 channels, and which G-protein coupled receptors within those cells activate them and finally how exactly HC-070 inhibits TRPC4 and TRPC5 dependent currents in neurons.

Taken together, the data presented provide a strong rationale for considering TRPC4 and TRPC5 as targets for the alleviation of symptoms associated with human psychiatric disorders. Given the profile observed, conditions such as major depressive disorder, obsessive-compulsive disorder, post-traumatic stress disorder, and generalized anxiety disorder could be amenable to treatment.

The mechanism of action, TRPC4 and TRPC5 inhibition, is distinct from current therapeutics and has the potential to impact multiple transmitter systems. The channels are downstream of numerous signaling pathways including any GPCRs that signal via G-alpha q as well as Gi and Go [5, 6, 10, 11]. In addition, activity of TRPC5 homomultimers is strongly potentiated by calcium [35], linking the activity of the channel to several other signaling events. Being mechanistically distinct, antagonists to TRPC4 and TRPC5 channels could have potential to provide relief to patients who do not respond to current therapies. The precise neurotransmitter systems and circuitry impacted by inhibition of TRPC4 and TRPC5 await further study. Such work will be useful in identifying relevant symptoms and patient populations most likely to respond to this mechanism of action.

This contribution to the understanding of the biological role of these channels was made possible by the invention and characterization of the highly potent and selective chemical antagonist to TRPC4 and TRPC5 that we call HC-070. HC-070 shows all the properties of a high-quality small molecule inhibitor. We believe this compound represents an important lead molecule with which to progress the understanding of TRPC channel biology. To enable these efforts, we are making HC-070 available to the research community free of charge. Interested parties should direct inquiries to: Christian.Eickmeier@Boehringer-Ingelheim.com.

## Materials and methods

### Cells

The cDNAs for various full-length ion channels were subcloned into pcDNA5/TO. Stable clonal T-REx-293 (Invitrogen) lines were selected after transfection with the plasmid of interest with 100 μg/mL hygromycin and 5 μg/mL blasticidin. Some cell lines stably expressed the human muscarinic type 1 or 2 receptor (M1R and M2R) under G418 selection (1 mg/mL). Cells were maintained according to manufacturer’s instructions. TRPC4 channels were the TRPC4beta isoform. Human hERG channels and human Kv1.3 channels were constitutively expressed in Chinese hamster ovary cell stable lines. CHO cells were cultured in DMEM/F-12 (1:1) supplemented with 10% FCS, 100 U/mL penicillin, 100 μg/mL streptomycin, 2 mM glutamine. Inducible cell lines were treated with tetracycline and plated on glass coverslips 18–24 hours before patch clamping.

### High-throughput screening

Cells stably expressing the human TRPC5 cDNA were plated at a density of ~35,000 cells/well in 384 well black wall clear bottom CellBind plates (Corning). Cells were induced with 1 μg/ml tetracycline, and allowed to grow for 20–30 hours. Cells were loaded with 25 μL of either 4 μM Fura-2/AM or 0.5 μM Fluo4/AM (Invitrogen) in Hank’s Balanced Salt Solution (HBSS; 0.185 g/L D-glucose, 0.9767 g/L MgS0_4_ (anhydrous), 0.4 g/L KCl, 0.06 g/L KH_2_PO_4_ (anhydrous), 0.35 g/L NaHCO_3_, 8.0 g/L NaCl, and 0.04788 g/L Na_2_HPO_4_ (anhydrous); pH 7.4) supplemented with 0.02% Pluronic acid for ~1 hour in the dark at room temperature. The dye was removed and replaced with 25 μl HBSS. Following recovery from loading, cells were assayed using the Hamamatsu FDSS 6000 system, which permitted illumination alternately at 340 nm and 380 nm for Fura-2 experiments, or at 485 nm for Fluo-4 experiments. Images were acquired at a rate of ~0.2 Hz. Baseline fluorescence of the plates was measured followed by addition of 26 μl of 50 μM test compound to each well. Fluorescence was monitored for 2 minutes then 13 μl high- Ca^2+^ Ringer’s solution (62.55 mM NaCl, 4.5 mM KCl, 62 mM CaCl_2_, 1 mM MgCl_2_, 10 mM HEPES, 10 mM Glucose pH 7.4) was added resulting in a final concentration of 14 mM Ca^2+^ and 10 μM test compound. Different concentrations of calcium were tested during assay development, and although lower concentrations of calcium elicited responses, 14 mM was chosen as the final concentration as it gave the best screening window. Data were collected for ~3 minutes following addition of high Ca^2+^ Ringer. Positive and negative control wells were included on each plate consisting of 200 μM of the promiscuous blocker 2-APB or HBSS alone, respectively.

For fluorescent IC_50_ determination the test compounds were serially diluted to concentrations of: 2.5 μM, 1.25 μM, 625 nM, 312.5 nM, 156.25 nM, 78.13 nM, 39.06 nM, 19.53 nM, 9.77 nM, 4.88 nM, 2.44 nM, 1.22 nM, 610 pM, 305 pM, 153 pM, 76 pM, 38 pM, 19 pM, 10 pM, 5 pM, and 2 pM. 8 replicates of the serial dilution were performed. The fluorometric assay was then performed as above so that the following final compound concentrations were achieved in the assays: 1 μM, 500 nM, 250 nM, 125 nM, 62.5 nM, 31.25 nM, 15.63 nM, 7.81 nM, 3.91 nM, 1.95 nM, 977 pM, 488 pM, 244 pM, 122 pM, 61 pM, 31 pM, 15 pM, 8 pM, 4 pM, 2 pM, and 1 pM. To construct concentration response curves the fluorescence intensity for each well at each time point was divided by the initial fluorescence intensity in that well. The response to agonist was determined by averaging the last four time points except the final time point. The percent inhibition was determined by comparing the average agonist response in the test wells with the average agonist response in the positive and negative control wells.

Screening of TRPC4 was performed essentially as above with the following differences: cells stably expressing human TRPC4 and the muscarinic acetylcholine receptor type 1 (M1) were used in the assay. The cells were activated by adding carbachol diluted in high calcium Ringer’s to a final concentration of 7 μM.

### Electrophysiology

Cells expressing the channel of interest were voltage clamped with an Axopatch 200B or 700B controlled by pClamp 10 (Molecular Devices). Pipettes were 1.2–3 Mohm resistance, and 60–75% of the series resistance was compensated. Currents were filtered at 2 kHz, and acquired at 10 kHz with a Digidata 1320 or 1321. The typical voltage protocol for TRP channel currents consisted of a holding potential of -40 mV, a 20 ms step to -120 mV, followed by a 400 ms ramp to +100 mV. These ramps were preceded by a 100 ms step to -80 mV, and immediately followed by a 15 ms step to +80 mV. In some recordings, the ramp was 400 ms from -80 to +80 mV (with 40 ms at ±80 mV, before and after the ramp). Ramps were applied every 5 s, and currents were averaged at ±80 mV, as the average of 5–20 ms at the end of the steps flanking the ramp (when any residual capacity current was finished flowing). Liquid junction potentials were not corrected.

The standard bath solution was Ringer’s solution (in mM) 145 NaCl, 4.5 KCl, 2 CaCl_2_, 1 MgCl_2_, 10 HEPES, 10 glucose, pH 7.4 (NaOH). 80 μM LaCl_3_ or 20 μM carbachol were added to this solution to activate TRPC5; 10 μM carbachol was used for TRPC4 recordings (the method of channel activation is detailed in Table 1). Cellular TRPC4 channels were not activated by the addition of lanthanum alone, and stimulation with carbachol and M1R activation yielded variable currents that desensitized rapidly. M2R stimulation via carbachol gave much more robust and long-lasting currents, so TRPC4 channel assays were performed using this activation method. The internal solution for TRPC4 and TRPC5 recordings contained (in mM) 140 Cs-aspartate, 1.91 CaCl_2_, 2.27 MgCl_2_, 10 HEDTA, 10 HEPES, pH 7.2 (with CsOH). The predicted free [Ca^2+^] of this solution is 1.4 μM. The internal solution for receptor-activated currents contained 0.3 mM Na-GTP and 1–4 mM ATP. All other components were the same. External solutions at the cell were switched using a gravity fed, local perfusion system.

For most ion channel selectivity experiments (S2 Table), the internal solution was K-aspartate based for potassium channel recordings and Cs-aspartate for all others (in mM, 140 K-aspartate or Cs-aspartate, 1.91 CaCl_2_, 2.27 MgCl_2_, 10 EGTA, 10 HEPES, pH 7.2 with KOH or CsOH). The predicted free [Ca^2+^] of this solution was 50 nM. The standard external solution was Ringer’s except for TRPA1, where we used a Ca^2+^-free Ringer’s (145 NaCl, 4.5 KCl, 3 MgCl_2_, 1 EGTA, 10 HEPES, 10 glucose, pH 7.4 with NaOH).

The standard bath solution for patch clamp experiments with K_V_7.1/minK expressing cells was (in mM): 140 NaCl, 4.0 KCl, 1.0 MgCl_2_, 1.8 CaCl_2_, 10 glucose, 10 HEPES (pH 7.4 with NaOH). The intracellular solution was (in mM): 120 K-aspartate, 5.0 EGTA, 5.0 HEPES, 2.0 Mg_2_-ATP, 0.3 Na_3_-GTP, 14.0 Na_2_-phosphocreatine, 50 U/mL creatine phosphokinase (pH 7.2 with KOH), 30 μM PMA (phorbol 12-myristate 13-acetate). Whole-cell recordings were made using an EPC-10 amplifier and PatchMaster software (HEKA, Lambrecht, Germany). The following voltage protocol was used: -80 mV (Vhold), 20 mV (2000 ms),-40 mV (500 ms), -120 mV (10 ms) (applied every 30 sec).

Patch clamp recordings of K_V_1.5 and K_V_4.3 were performed at bSys (Basel, Switzerland), as follows: CHO cells stably expressing either K_V_1.5 or K_V_4.3/KChiP2 were voltage clamped at a holding potential of -80 mV and test steps were applied to +40 mV (500 ms at 0.1 Hz for K_V_1.5 and 5000 ms at 0.05 Hz for K_V_4.3). The peak current during the step to +40 mV was analyzed.

IC_50_ measurements were made as follows: After seal formation, break-in, and the setting of parameters for series resistance compensation, the current was activated as indicated in Fig 2 and Table 1, as well as S1 and S2 Tables. In some cases, the current reached a steady-state that remained stable, at which point the test compound application began. In other cases, the current reached a peak and then began to decrease, reflecting either desensitization and/or channel run-down. For these cases, we waited to apply test compound until the current decrease slowed down (which varied from cell to cell). After the current reached a new steady-state following test compound addition, we applied compound-free external solution to determine the extent of current recovery. In some recordings, recovery was preceded by application of control blocker (to determine the amount of leak current) or by a higher concentration of test compound (e.g., Fig 2 panel A). Throughout the experiment, we monitored the shape of the currents during the test pulse or ramp.

To calculate the percent block, the current remaining after compound addition was measured and compared to the predicted unblocked current (after leak was subtracted from both). This was predicted as the linear interpolation of the current amplitudes in control external solution immediately before compound addition and after compound washout (Fig 2 panel A). This process, when updated with multiple recovery periods throughout the recording, accounts for some of the cell to cell variability in run-down.

Typically 3–4 concentrations of test compound were applied to a single cell. The percent blocks from individual cells were fitted with the Hill equation (using Levenberg-Marquardt algorithm, base and max block set to 0 and 100%, respectively, IC_50_ and Hill slope allowed to vary). The resulting IC_50_s were then averaged.

### Selectivity profiling

All other selectivity work was performed at CEREP, as described in S1 and S2 Reports. Data are reported as the per cent inhibition at 1 μM.

### Solubility assay

Solubility in Normal Ringer Solution was determined by dissolving a standard range of volumes of 10 mM DMSO stock of HC-070 in Normal Ringer’s Solution at room temperature. Following vortex and incubation for 40 minutes at room temperature, solutions were filtered, quenched, and analyzed by liquid chromatography. Solubility limits were determined by comparison to a standard curve generated by dissolving compound in acetonitrile.

### Slice recordings

Coronal brain slices (250 μm) containing the basolateral amygdala were prepared from male C57BL/6 mice using a VT1200S vibratome (Leica). Slices were continuously superfused in solution containing the following (in mM): 125 NaCl, 2.5 KCl, 1.8 CaCl_2_, 0.8 MgCl_2_, 1.3 NaH_2_PO4, 26 NaHCO_3_, 25 glucose and 0.5 Na- L-Lactate and equilibrated with 95% O_2_ and 5% CO_2_, pH 7.3–7.4, at 22–23°C.

Whole-cell recordings of postsynaptic currents were obtained from pyramidal neurons in the basolateral amygdala under visual guidance (infrared optics) with an EPC-10 amplifier and PatchMaster 2x73 software (HEKA Elektronik). In voltage-clamp experiments, the recording patch electrodes (2.5–4 MΩ resistance) contained the following (in mM): 110 KCl, 10 NaCl, 1 MgCl_2_, 0.5 CaCl_2_, 10 HEPES, 4 MgATP, 0.4 NaGTP and 10 phosphocreatine di(tris)salt (adjusted to pH 7.2 with Tris(hydroxymethyl) aminomethane. CCK-4 and the HC-070 were applied to the superfusion solution via a gravity-driven application system.

Currents were filtered at 2.9 kHz and digitized at 10 kHz. Spontaneous postsynaptic currents were detected using Mini Analysis 6.0.3 software (Synaptosoft) and the amplitude of currents was measured as the difference between the mean current during a pre-current baseline over a 2 ms window and the current at its peak.

### Animal Welfare

All experimental procedures were authorized by the Local Animal Care and Use Committee in accordance with local animal care guidelines, AAALAC regulations and the USDA Animal Welfare Act. For the EPM, some FST, marble burying, experiments were approved by the Hydra Biosciences Institutional Animal Care and Use Committee (IACUC), governed by the rules and regulations of the Cambridge Public Health Department and the Commissioner of Laboratory Animals (Cambridge, MA, USA). The elevated plus maze with CCK-4, some FST, TST and locomotor activity experiments were conducted at Boehringer Ingelheim, Biberach, Germany, in accordance with the German and European Animal Welfare Act and authorized by the Regierungspräsidium Tübingen as the responsible local German authority. The chronic social defeat work was conducted under a permit (170/2012) issued by the Veterinary Office, Zurich, Switzerland. All efforts were made to minimize the number of mice used and any stress to the animals.

### Pharmacokinetic studies

For the intravenous (IV) group HC-070 was dissolved in 4% DMSO, 10% Solutol HS-15, and 86% water and administered as a bolus via the tail vein (0.25 mg/mL @ 4 mL/kg = 1 mg/kg) to male C57BL/6 mice or Swiss Webster mice. Oral doses were given at 1 mg/mL at a 10 mL/kg dose volume to yield a final dose of 10 mg/kg in the fed state. A simple suspension of HC-070 in 0.5% methyl cellulose was used for oral administration. 12 animals were dosed per arm.

Two blood samples per mouse were taken, either via retroorbital or submandibular bleed at pre-determined time points following compound administration. The second sampling was done under terminal anaesthesia. Blood samples were added to EDTA microtainers and placed on wet ice for no longer than 30 minutes prior to being spun at 8000 rpm for 5 minutes to yield at least 100 μL of plasma. Plasma samples were immediately drawn off, transferred to 1.7 mL Eppendorf tubes, and placed on dry ice/-80°C prior to analysis. One plasma sample from each of 3 mice was collected per sampling time point. In the intravenous arm of the study with Swiss Webster mice, two of the 24 samples could not be taken and the analytical data for one sample were invalid resulting in 21 valid data points for this arm of the study.

At study termination, animals were sacrificed via CO_2_ inhalation and blood was collected. The collection timepoints were recorded and used in subsequent analysis.

### Bioanalytical

Plasma samples along with samples of dose solution were analyzed by protein precipitation followed by liquid chromatography-tandem mass spectrometry (LC-MS/MS). The triple quadrupole mass spectrometer was operated in positive ion mode. Brain was homogenized in ice cold water (2 mL/brain) and diluted 1 to 4 in plasma prior to analysis. Brain slices were homogenized in ice cold water (40μL/10mg brain slice) without further dilution prior to quantification.

### Calculating PK properties

The HC-070 concentration data were determined and the concentration-time relationship was analyzed by a pharmacokinetic data analysis software package (ToxKin^®^, Version 3, Boehringer Ingelheim, Germany) to obtain pharmacokinetic parameters. AUC was calculated using the linear trapezoidal rule for ascending and the logarithmic trapezoidal rule for descending parts of the concentration-time curve. For IV dosing, concentration at time zero was estimated by linear interpolation of the concentrations from the two first sampling points.

### Brain homogenate assay

Frozen rat brains were homogenized in PBS buffer (dilution factor D = 4). The homogenate was spiked with test compounds (1 μM final concentration, final concentration of DMSO was 0.1%) and subsequently dialyzed against PBS buffer for 8 hrs at 37°C in rapid equilibrium dialysis cells (RED-device, Pierce). At the end of the dialysis period, the dialysate was transferred into reaction tubes and mixed with an analytical internal standard. Tissue homogenate was added to buffer samples and buffer was added to tissue samples to ensure identical matrix composition for bioanalytical measurement. After protein precipitation using acetonitrile, samples were measured by LC-MS/MS. The free fraction was calculated as fu,app = (C_PBS_/C_homogenate_). Since the brains were homogenized in the buffer, the observed free fraction values (fu,app) were extrapolated to the fractions unbound in the brain (fu*) based on the dilution (D) using the following formula:

fu*=(1/D)/((1/fu,app−1)+1/D).

Unbound brain concentrations are very comparable over species [36] allowing the calculation of unbound mouse brain concentrations by using rat brain homogenate data.

### Elevated plus maze with CCK-4

The EPM is a cross-shaped platform consisting of four equally sized arms (50 cm × 10 cm) elevated 50 cm above ground. There are 2 closed arms (opposing each other), flanked by 40-cm opaque walls, while the remaining two arms are without walls (open arms). All arms are connected via a central zone (10 cm × 10 cm), allowing animals to move freely into each zone of the maze.

Male C57BL/6 mice were obtained from Charles River Labs (Charles River, Germany). Animals weighing 30±1 g were housed in groups of five and kept at 21–23 °C under a 12-h regular light/dark cycle with lights on at 6:00 a.m.

For this paradigm, light intensities in the central zone were set to 100 lux. Animals were habituated to experimental conditions, but not to the maze. Their movements on the platform were monitored for a 5-min period with a camera. The animal was initially placed in the central zone facing an open arm. Percentage of time spent in open arms (time in open arms/[time in open arms + time in closed arms] × 100), as well as the number of entries in opened and closed arms were analyzed with the ANY-maze software (Stoelting Co.). A zone entry was defined as the presence of 85% of the animal’s body in a specific zone.

### Standard elevated plus maze

The room was illuminated with fluorescent lighting on a 12-hour light/dark cycle. The light cycle was reversed, so that the dark cycle was from 6am-6pm daily and studies were performed when animals were more active. Groups of male C57/BL6 mice (10 weeks old, Jackson Labs, Bar Harbor, ME, USA) were dosed PO with 0.5% methyl cellulose or HC-070 at 0.3, 1 or 3 mg/kg (n = 10). The positive control, 1.5 mg/kg diazepam, was administered IP 30 minutes prior to testing (n = 10). Immediately following dosing, mice were returned to their home cage. At 60 minutes post vehicle or HC-070 administration, and 30 minutes post diazepam administration, mice were placed onto the elevated plus maze (0547M, Mouse Elevated Plus Maze, Columbus Instruments, Columbus, OH), one at a time, and their session recorded for 5 minutes. Videos were manually scored for number of open arm entries by a scorer blinded to treatment. All animals that fell off the maze during the test were removed from analysis.

Separate groups of satellite animals (n = 3/group) were dosed with 0.3, 1 and 3 mg/kg HC-070. These animals were sacrificed via CO_2_ narcosis at 1 hour post dose and plasma and brains harvested to determine levels of HC-070.

### Chronic social defeat

C57BL/6J mice bred in-house (Zurich) were used in these experiments. Male offspring were weaned aged 3 weeks and caged in groups of 2–3 littermates. Mice were aged 12 weeks and weighed 26–30 g at study onset. Aggressor mice were male CD-1 (Janvier Labs, Saint-Berthevin, France). They were aged 8 months, were ex-breeders, and caged singly. Mice were maintained on a reversed 12:12 h light-dark cycle in an individually-ventilated caging system.

Chronic social defeat (CSD) was conducted using a refined protocol as described elsewhere [37]. Briefly, a C57BL/6 (CSD) mouse was housed singly in the home cage of a CD-1 mouse separated by a transparent, perforated divider. The CSD mouse was placed with the CD-1 mouse for either a cumulative total of 60 sec physical attack or 10 min maximum. To avoid bite wounds, the lower incisors of CD-1 mice were trimmed every third day across CSD. Each day for 15 days, the CSD x CD-1 mouse pairings were rotated so that CSS mice were confronted daily with a novel CD-1 mouse. On days 16–18, CSD mice remained adjacent to the same CD-1 mouse without any further attacks. Control (CON) mice remained in littermate pairs and were handled and weighed daily. Mice were weighed daily, and a reduction below 90% body weight at experiment onset was used as one discontinuation measure; as in previous CSD studies (e.g. 19, 20), no mouse went below 95% onset body weight on any day during the experiment and all mice maintained or increased absolute body weight across the experiment. After each CSD session mice were checked for bite wounds: as noted above, a major refinement of our CSD protocol over similar protocols [38] is that the lower incisors of CD-1 mice are trimmed regularly to prevent bite wounds. This refinement, and also that of observing and limiting each CSD session to a maximum of 60 s attack time, results in the complete absence of deep bite wounds (skin penetration) (19, 20). A localized surface wound (skin abrasion) was received once by each of four CSD mice and the discontinuation measure of three surface wounds did not occur in any mouse.

### Pavlovian fear learning and memory

Behavioral testing was conducted under dim lighting in a room adjacent to the holding room, using a Multi-Conditioning System with an infrared movement-detection system (TSE Systems GmbH, Bad Homburg, Germany) [39]. The test comprised four stages, each conducted in the same arena (context): pre-exposure to the arena (context), tone-footshock fear conditioning, fear expression to the context and fear expression to the tone. The tone served as conditioned stimulus (CS); it was paired with footshock which was the aversive unconditioned stimulus (US). The conditioning and expression stages of Pavlovian learning are inter-dependent in that what is learned during the conditioning stage determines what can be consolidated and recalled during the expression stage. Freezing was the readout for fear conditioning and expression, defined as no detectable movement for at least 2 sec; freezing was measured as percent time spent in this state. Locomotor activity was also measured continuously.

#### Context pre-exposure

The mouse was placed on the grid floor in the arena (context) for 15 min, and activity and % time freezing were recorded continuously.

#### CS-US fear conditioning

The next day, the mouse was placed on the grid floor in the same context and, following 5-min adaptation, exposed to six trials of a discrete, neutral tone of 5 kHz and 85 dB (CS) presented via a speaker for 20 seconds; the final 2 seconds were contiguous with a 2 second x 0.15 mA footshock (US). The inter-trial interval (ITI) was 120 s. Percent time freezing was measured during the six CSs and five ITIs; for analysis, these trials were grouped into CS blocks 1–2, 3–4, 5–6.

#### Context fear expression

The next day, the mouse was placed on the grid floor in the same arena for 21 min. Percent time freezing was measured and for analysis time was grouped into 3-min blocks.

#### CS fear expression

Immediately after the context fear expression test, mice were exposed to 12 trials of the tone CS for 30 sec and with an ITI of 90 sec. Percent time freezing was measured during the 12 CSs and 11 ITIs; for analysis, trials were grouped into CS blocks 1–3, 4–6, 7–9, 10–12 and ITIs 1, 2–3, 4–5, 6–7, 8–9, 10–11.

#### Experimental design

In week 1 of each experiment, mice were handled on five consecutive days. In a first experiment, we investigated for effects of HC-070 on Pavlovian fear learning and memory in otherwise non-manipulated mice. Mice (n = 10 per group) were allocated at random to the 0, 1 or 3 mg/kg group, and the same dose was administered at 2 h before the CS-US conditioning test and, on the next day, at 2 h before the onset of the two expression tests. In a second experiment, we investigated for effects of HC-070 on Pavlovian fear learning and memory in CSD versus control (CON) mice. An initial context pre-exposure test was conducted prior to CSD to determine baseline freezing levels; these were used to counterbalance allocation of mice to CSD and CON groups. Following 15 days of CSD/CON, within each group mice were allocated at random to vehicle (VEH) or HC-070 1 mg/kg, giving four groups and 12 mice per group. On day 16 another context pre-exposure test was conducted, on day 17 the CS-US conditioning test, and day 18 the two expression tests. VEH or HC-070 were administered at 5 min after the CS-US conditioning test and at 1 h prior to the onset of the two expression tests. At the end of testing mice were decapitated and trunk blood was collected into EDTA-coated tubes.

### Marble burying

Groups of female C57BL/6 mice (7 weeks old, Charles River Laboratories, Wilmington, MA USA) were dosed PO with 0.5% methyl cellulose or HC-070 at 1, 3 or 10 mg/kg (n = 10). The positive control, 10 mg/kg zimelidine, was administered intraperitoneally 45 minutes prior to testing (n = 10). Immediately following dosing, mice were returned to their home cage. At 60 minutes post vehicle or HC-070 administration, and 45 minutes post zimelidine administration, mice were placed into the larger cages containing 20 black glass marbles evenly spaced over 5 cm of BetaChip bedding for a 30 minute test session. Upon completion, the animals were carefully removed and each cage was photographed for later review. The animals were sacrificed via CO_2_ narcosis upon removal from the cages and plasma was harvested to determine levels of HC-070.

Photos were manually scored for number of unburied marbles (>25% visible) by a scorer blinded to treatment. The number of buried marbles is equal to the number of unburied marbles subtracted from 20.

### Tail suspension test

NMRI mice were habituated to their room for at least 2 to 3 weeks after the delivery and kept under standard conditions in groups of 4–5 animals per cage. Food and water were available ad libitum.

The tail suspension test was performed using with a BIOSEB system. In preparation for the experiment, a piece of tape (approx. 15 cm long) was wrapped around the end of the mouse tail approximately 2 mm before the tip. The tape was then attached to a holding device within the measuring unit at a height of approximately 30cm.

Movements of the animals were monitored and automatically evaluated by corresponding software. The duration of immobility during 6 min observation period was recorded. At the end of the experiment plasma samples were taken and the animals were sacrificed via isofluorane inhalation and subsequent decapitation.

### Locomotor activity

NMRI mice were habituated to their room for at least 2 weeks after the delivery and kept under standard conditions in groups of 4–5 animals per cage. Food and water were available ad libitum.

Spontaneous motility and rearing were measured by an automated system (Coulbourn Instruments, USA). Animals were introduced into transparent square cages (40 cm length) that are equipped with 2 light barrier systems in order to monitor the movements as well as rearing behavior automatically. The movements of the animals were recorded for 30 minutes. At the end of the experiment, the animals were sacrificed via isofluorane inhalation and subsequent decapitation.

### Forced swim test

For the initial forced swim test work performed in the US, Groups of male CD-1 mice (7 weeks old, Charles River Labs, Wilmington, MA, USA) were dosed PO with 0.5% methyl cellulose or HC-070 at 0.3, 1 or 3 mg/kg (n = 12/dose). The positive control, imipramine, was administered IP at 20 mg/kg (n = 12). Immediately following dosing, mice were returned to their home cage. At 60 minutes post vehicle or HC-070 administration, and 45 minutes post imipramine administration, 4 mice were placed individually into chambers filled with 15 cm of water, maintained at a temperature of 24±1°C. After a 6-min swim session mice were towel-dried and returned to the home cage.

Digital video output of swim sessions was analyzed by a computer running Noldus (Ethovision XT Version 8 Video Tracking, Noldus Information Technology, Wageningen, Netherlands) software. Individual mouse immobility time is acquired and the last 4 minutes of the 6-minute swim session analyzed. The software traces each animal’s displacement in a defined arena; images of these displacements are acquired five times per second.

For the confirmatory forced swim test work performed in Germany, male C57BL/6 mice were obtained from Charles River Labs (Charles River, Germany). HC-070 was administered 1 hour prior to testing. Mice were gently placed into a 1 liter glass beaker (height: 14.5 cm, diameter: 11 cm) containing 650 ml (7.5 cm height) water at temperature of 21–22°C, for 5 min.

During the FST, animals were constantly monitored by video recording and data collected for the entire 5 min period. The duration of animal immobility within the last 2 min was evaluated. A mouse was considered immobile when it ceased struggling and remained floating in the water making only those movements necessary to keep its head above the water. Animals were assigned to dose groups at random. Groups were equally divided among five test beakers. The positive control desipramine was administered IP at 15 mg/kg (8 ml/kg) 60 min prior to the test. After a 5 min swim session mice were towel-dried under heated red light and returned to the home cage. Aliquots of each dosing solution were collected on each study day and stored at -20°C prior to analysis. Ten to fifteen minutes after FST, blood was collected into tubes containing EDTA (1.6 mg/ml) and centrifuged at 3000 x g for 20 min at 4°C. Then, plasma was stored at -20°C until compound level determination.

For the PK/PD modeling, results were combined from both sets of forced swim test work.

### Statistical analysis

For the analysis of the slice recordings, a two-way ANOVA followed by a Tukey’s multiple comparison’s test was used to compare the EPSCs evoked by CCK-4 in the presence and absence of HC-070.

For all acute in vivo tests, a one-way ANOVA was used to determine statistical significance for effect of HC-070 versus the vehicle followed by a Dunnett’s test for post-hoc significance. A one or two-tailed t-test was used to determine the statistical significance of the reference compounds versus the vehicle control group.

For the fear conditioning and chronic social defeat work, statistical analysis of % time spent freezing was conducted using SPSS (version 20, SPSS Inc., Chicago IL, USA) and mixed model ANOVA. In the first experiment the design was drug dose (0, 1, 3 mg/kg) X Trial-block (ITI or CS trial blocks), and in the second experiment the design was Group (CON-VEH, CON-Drug, CSS-VEH, CSS-Drug) X Trial-block (ITI or CS trial blocks). Significant effects were analyzed using Fisher’s least significant difference (LSD) *post hoc* test.

## Supporting information

S1 Table Potency of HC-608 at recombinant TRPC4 and TRPC5 containing channels. : HC-608 inhibits recombinantly expressed TRPC4 and TRPC5 as well as TRPC1-containing heteromultimers in whole-cell manual patch clamp. Listed IC_50_ values are mean ± S.D. *—The IC_50_ was calculated by combining percent inhibition at 1–2 concentrations measured in multiple, different cells, and then by data fitting with the Hill equation; the listed S.D. was determined from the curve fitting.
(DOCX)

S2 Table Summary of the selectivity of HC-070 and HC-608 for various ion channels relative to TRPC5, as determined by electrophysiology. Where specified, values are means ± S.D. The fold-selectivity is calculated relative to the carbachol activated human TRPC5 IC_50_. N.D. indicates the experiment was not done.
(DOCX)

S3 Table Summary of the effects of the HC-070 on receptors, kinases, channels and transporters in binding or enzyme inhibition assays. (DOCX)

S4 Table Summary of the effects of the HC-608 on receptors, kinases, channels and transporters in binding or enzyme inhibition assays. (DOCX)

S1 Fig Time course of responses following compound and agonist addition in TRPC5 and TRPC4 expressing cells. (A & B) Time course of cells expressing TRPC5 in the fluorometric assays. Cells plated into 384 black wall clear bottom plates were loaded with the fluorescent calcium indicator Fluo-4AM and the fluorescence intensity of each well was determined periodically over the course of ~5 minutes. The raw fluorescence in each well at each time point was divided by the initial fluorescence in each well. HC-070 (A) or HC-608 (B) (blue symbols) at the indicated concentration was added at the indicated time point and cells were incubated for ~2 minutes. High calcium buffer was added at the time point indicated and the responses monitored for ~2.5 minutes. Values at the indicated time points were averaged to determine the response to agonist. Negative control wells (black symbols) which received only the compound vehicle and assay buffer were included on each plate as were positive control wells (grey symbols) containing the 2-APB. (C & D) Time course of cells expressing TRPC4 in the fluorometric assays. Cells plated into 384 black wall clear bottom plates were loaded with the fluorescent calcium indicator Fluo-4AM and the fluorescence intensity of each well was determined periodically over the course of ~5 minutes. The raw fluorescence in each well at each time point was divided by the initial fluorescence in each well. HC-070 (C) or HC-608 (D) (blue symbols) at the indicated concentration was added at the indicated time point and cells were incubated for ~2 minutes. High calcium buffer containing Carbachol (7 μM final concentration) was added at the time point indicated and the responses monitored for ~2.5 minutes. Values at the indicated time points were averaged to determine the response to agonist. Negative control wells (black symbols) which received only the compound vehicle and assay buffer were included on each plate as were positive control wells (grey symbols) containing the 2-APB.
(TIF)

S2 Fig Pharmacokinetic properties of HC-070. PK profiles of HC-070 after (A) intravenous and (B) oral administration in Swiss Webster mice. Plasma concentrations were determined by LC-MS/MS. Points represent the individual concentrations at the times indicated. Lines represent mean exposure (n = 12 mice/arm). (C) Summary of PK properties. CL = clearance; V_ss_ = volume of distribution at steady state; MRT_disp_ = mean residence time of drug molecules after intravascular administration; T_1/2_ = half-life. (D) Plasma and brain concentrations measured 2 hours after intravenous or oral administration of 1 or 10 mg/kg HC-070, respectively. C_PL_ = concentration in plasma, C_BR_ = concentration in brain, K_P,BR_ = partitioning coefficient between brain and plasma.
(TIF)

S3 Fig Control experiment for the chronic social defeat-fear conditioning study. (A) Without drug administration, when placed in an unfamiliar arena (context), mice showed a low level of freezing, a fear behavior. (B) The next day, mice were administered vehicle, 1 or 3 mg/kg HC-070 PO and 2 hours later were placed in the same context, and exposed to a tone conditioned stimulus (CS) that announced an electroshock unconditioned stimulus (US). Mice acquired increased CS freezing across successive pairings of the CS and US, indicating the learning of fear of the CS, without an effect of HC-070. (C) The next day, mice received vehicle, 1 or 3 mg/kg HC-070 PO (same dose allocation as on previous day) and 2 hours later were placed in the same context in which CS-US conditioning took place the previous day. HC-070 was without effect on context fear memory, as indicated by each drug group showing low and similar freezing levels. (D) Immediately after the context memory test, mice were presented with the tone CS memory test. Freezing to the CS was higher than to the context, but there was still no effect of HC-070 on CS fear memory in these otherwise non-manipulated mice.
(TIF)

S4 Fig Effects of positive control PCP on locomotor activity. After 90 minutes of habituation to the chamber, mice were administered vehicle or 5 mg/kg PCP subcutaneously. Activity was recorded for another 60 minutes. PCP substantially increased activity, as expected. (2-way ANOVA followed by a Dunnett’s multiple comparison’s test, p<0.0001; n = 8/group).
(TIF)

S1 Report *In vitro* Pharmacology Study of HC-070. (PDF)

S2 Report *In vitro* Pharmacology Study of HC-608. (PDF)
