# Supplementary material for: Treatment with HC-070, a potent inhibitor of TRPC4 and TRPC5, leads to anxiolytic and antidepressant effects in mice
Source: PLoS One. 2018 Jan 31;13(1):e0191225. doi: 10.1371/journal.pone.0191225 (PMC5791972; doi:10.1371/journal.pone.0191225)
Supplement: S1 Table — : HC-608 inhibits recombinantly expressed TRPC4 and TRPC5 as well as TRPC1-containing heteromultimers in whole-cell manual patch clamp. Listed IC50 values are mean ± S.D. *—The IC50 was calculated by combining percent inhibition at 1–2 concentrations measured in multiple, different cells, and then by data fitting with the Hill equation; the listed S.D. was determined from the curve fitting. (DOCX) [file pone.0191225.s001.docx]

|  |  | **HC-608** | |
| --- | --- | --- | --- |
| **CHANNEL** | **CURRENT ACTIVATION** | **Cells Tested** | **IC_50_ (nM)** |
| mTRPC5 | La^3+^ (80 µM) | 3 | ~0.62 ±0.30 |
| hTRPC5 | La^3+^ (80 µM) | 7 | ~0.17 ± 0.04 |
| rTRPC5 | La^3+^ (80 µM) | 3 | ~0.41 ± 0.12 |
| hTRPC5 | 20 µM carbachol /M1R | 5 | ~0.67 ± 0.21 |
| mTRPC4 | 10 µM carbachol /M1R | 5 | ~0.45 ± 0.03 |
| hTRPC4 | 10 µM carbachol /M1R | 3 | ~0.17 ± 0.05 |
| hTRPC1/hTRPC5 | La^3+^ (80 µM) | 3 | ~1.4 ± 0.5 |
| hTRPC1/hTRPC5 | 20 µM carbachol /M1R | 4 | ~4.7 ± 2.8 |
