# Supplementary material for: Treatment with HC-070, a potent inhibitor of TRPC4 and TRPC5, leads to anxiolytic and antidepressant effects in mice
Source: PLoS One. 2018 Jan 31;13(1):e0191225. doi: 10.1371/journal.pone.0191225 (PMC5791972; doi:10.1371/journal.pone.0191225)
Supplement: S2 Table — Where specified, values are means ± S.D. The fold-selectivity is calculated relative to the carbachol activated human TRPC5 IC50. N.D. indicates the experiment was not done. (DOCX) [file pone.0191225.s002.docx]

|  |  | **HC-070** | | | **HC-608** | | |
| --- | --- | --- | --- | --- | --- | --- | --- |
| **CHANNEL** | **CURRENT ACTIVATION** | **Cells Tested** | **IC_50_ (μM)** | **Selectivity** | **Cells Tested** | **IC_50_ (μM)** | **Selectivity** |
| hTRPV1 | 500 nM Capsaicin | 3 | >1.9 | >760 | 3 | >1.64 | >2000 |
| hTRPV3 | 30 μM 2-APB | 3 | >1.9 | >760 | 3 | >1.64 | >2000 |
| hTRPA1 | 2-10 μM AITC | 3 | >1.9 | >760 | 3 | >1.64 | >2000 |
| TRPM8 | 100 μM menthol | 3 | 1.9±0.3 | ~760 | 3 | >1.64 | >2000 |
| TRPC3 | 1 μM carbachol | 3 | 1.0±0.1 | 400 | N.D. | - | - |
| TRPC6 | 5 μM OAG | 3 | >1.9 | >760 | 3 | >1.64 | >2000 |
| TRPC7 | 250 nM carbachol | 3 | >1.9 | >760 | N.D. | - | - |
| hERG | Voltage (-40 mV tail) | 3 | ~1.9±0.2 | ~760 | 4 | 1.15±0.13 | >1430 |
| K_v_1.3 | Voltage (+20 mV) | 3 | ~1.9±0.3 | ~760 | 5 | 1.05±0.34 | >1310 |
| K_v_1.5 | Voltage (+40 mV) | 2 | >1.9 | >760 | N.D. | - | - |
| K_v_4.3 | Voltage (+40 mV) | 2 | >1.9 | >760 | N.D. | - | - |
| K_v_7.1 | Voltage (+20 mV) | 3 | >1.9 | >760 | N.D. | - | - |
| Ca_v_1.2 | Voltage (0 mV) | 3 | >1.9 | >760 | 8 | >1.64 | >2000 |
| Na_v_1.2 | Voltage (+20 mV) | 3 | >1.9 | >760 | 4 | >1.64 | >2000 |
| Na_v_1.5 | Voltage (+20 mV) | 3 | >1.9 | >760 | 3 | >1.64 | >2000 |
