# Supplementary material for: Treatment with HC-070, a potent inhibitor of TRPC4 and TRPC5, leads to anxiolytic and antidepressant effects in mice
Source: PLoS One. 2018 Jan 31;13(1):e0191225. doi: 10.1371/journal.pone.0191225 (PMC5791972; doi:10.1371/journal.pone.0191225)
Supplement: S3 Table — (DOCX) [file pone.0191225.s003.docx]

| **Target** | **Species** | **Assay type** | **% Inhibition** | | |
| --- | --- | --- | --- | --- | --- |
|  |  |  | 1st | 2nd | Mean |
| a_1A_ Adrenergic receptor | human | Antagonist binding | -6.1 | 3.1 | -1.5 |
| a_2B_ Adrenergic receptor | human | Antagonist binding | -22.3 | -1.2 | -11.7 |
| a_2C_ Adrenergic receptor | human | Agonist binding | 7.9 | -1.0 | 3.5 |
| BZD Benzodiazepine Receptor | rat | Agonist binding | -26.0 | -9.6 | -17.8 |
| CB_1_ Canabinoid Receptor | human | Agonist binding | 8.4 | 18.8 | 13.6 |
| D_1_ Dopamine Receptor | human | Antagonist binding | 3.0 | -4.8 | -0.9 |
| D2S Dopamine Receptor | human | Agonist binding | -3.0 | 3.4 | 0.2 |
| D3 Dopamine Receptor | human | Antagonist binding | 3.6 | 6.4 | 5.0 |
| D4.4 Dopamine Receptor | human | Antagonist binding | -15.8 | -1.8 | -8.8 |
| GABAA1 (α1,β2,γ2) Receptor | human | Agonist binding | 27.9 | 16.4 | 22.1 |
| NMDA Receptor | rat | Antagonist binding | 4.4 | 25.7 | 15.1 |
| TNF-α Receptor | human | Agonist binding | -5.4 | -5.8 | -5.6 |
| H1 Histamine Receptor | human | Antagonist binding | -15.6 | -11.4 | -13.5 |
| MT3 (ML2) Melatonin Receptor | hamster | Agonist binding | 46.1 | 49.5 | 47.8 |
| Monoamine Oxidase A | rat | Antagonist binding | 12.6 | 13.1 | 12.8 |
| M5 Muscarinic Receptor | human | Antagonist binding | -7.3 | 10.1 | 1.4 |
| δ2 Opioid Receptor | human | Agonist binding | 6.8 | 5.9 | 6.3 |
| Glutamate Receptor (PCP) | rat | Antagonist binding | -7.6 | -10.0 | -8.8 |
| 5-HT1A Serotonin Receptor | human | Agonist binding | 8.7 | -24.2 | -7.7 |
| 5-HT2A Serotonin Receptor | human | Agonist binding | -1.3 | 6.1 | 2.4 |
| 5-HT2C Serotonin Receptor | human | Agonist binding | -13.4 | -9.5 | -11.5 |
| 5-HT3 Serotonin Receptor | human | Antagonist binding | 4.7 | 2.6 | 3.7 |
| Sigma Receptor (non-selective) | human | Agonist binding | 0.3 | 5.1 | 2.7 |
| Androgen Receptor | human | Agonist binding | 11.1 | 3.2 | 7.1 |
| SKCa Potassium Channel | rat | Antagonist binding | -10.1 | 3.5 | -3.3 |
| Na+ channel (site 2) | rat | Antagonist binding | -6.9 | -12.5 | -9.7 |
| Cl- channel (GABA-gated) | rat | Antagonist binding | 24.2 | 17.5 | 20.9 |
| Norepinephrine transporter | human | Antagonist binding | -10.1 | -0.6 | -5.4 |
| Dopamine transporter | human | Antagonist binding | 8.9 | 8.6 | 8.8 |
| GABA transporter | rat | Antagonist binding | -3.4 | -5.2 | -4.3 |
| 5-HT transporter | human | Antagonist binding | -0.3 | 18.4 | 9.1 |
| PDE5 (non-selective) | human | Enzyme inhibition | 27.3 | 50.4 | 38.9 |
| Acetylcholinesterase | human | Enzyme inhibition | 2.1 | -0.7 | 0.7 |
| GABA transaminase | rat | Enzyme inhibition | -33.1 | -36.0 | -34.6 |
| Tyrosine Hydroxylase | rat | Enzyme inhibition | -27.2 | -18.7 | -23.0 |
