# Supplementary material for: Treatment with HC-070, a potent inhibitor of TRPC4 and TRPC5, leads to anxiolytic and antidepressant effects in mice
Source: PLoS One. 2018 Jan 31;13(1):e0191225. doi: 10.1371/journal.pone.0191225 (PMC5791972; doi:10.1371/journal.pone.0191225)
Supplement: S4 Table — (DOCX) [file pone.0191225.s004.docx]

| **Target** | **Species** | **Assay type** | **% Inhibition** | | |
| --- | --- | --- | --- | --- | --- |
|  |  |  | 1st | 2nd | Mean |
| a_1A_ Adrenergic receptor | rat | Antagonist binding | -2.8 | 1.1 | -0.8 |
| a_2B_ Adrenergic receptor | human | Antagonist binding | -7.4 | -0.7 | -4 |
| a_2C_ Adrenergic receptor | human | Antagonist binding | -4.1 | -10.2 | -7.1 |
| BZD Benzodiazepine Receptor | rat | Agonist binding | -3 | -31.5 | -17.3 |
| CB_1_ Canabinoid Receptor | human | Agonist binding | 27 | 25.5 | 26.2 |
| D_1_ Dopamine Receptor | human | Antagonist binding | 1.1 | 5 | 3.1 |
| D3 Dopamine Receptor | human | Antagonist binding | 4 | 9.7 | 6.9 |
| D4.4 Dopamine Receptor | human | Antagonist binding | -20.4 | 3.4 | -8.5 |
| GABA_A1_ (α1,β2,γ2) Receptor | human | Agonist binding | 0.6 | 7.9 | 4.2 |
| NMDA Receptor | rat | Antagonist binding | 19 | 3.2 | 11.1 |
| TNF-α Receptor | human | Agonist binding | 8.8 | -8.6 | 0.1 |
| H1 Histamine Receptor | human | Antagonist binding | -8.1 | -0.9 | -4.5 |
| MT3 (ML2) Melatonin Receptor | hamster | Agonist binding | -0.7 | 1.2 | 0.2 |
| Monoamine Oxidase A | rat | Antagonist binding | -3.8 | 3.6 | -0.1 |
| M5 Muscarinic Receptor | human | Antagonist binding | -15.6 | -12.8 | -14.2 |
| δ2 Opioid Receptor | human | Agonist binding | -10.8 | 3.1 | -3.9 |
| Glutamate Receptor (PCP) | rat | Antagonist binding | -9 | -0.1 | -4.6 |
| Phosphodiesterase (rolipram) | mouse | Antagonist binding | -4.6 | 0.5 | -2.1 |
| 5-HT1A Serotonin Receptor | human | Agonist binding | -0.2 | 14.8 | 7.3 |
| 5-HT2A Serotonin Receptor | human | Agonist binding | 1.8 | -14.3 | -6.2 |
| 5-HT2C Serotonin Receptor | human | Agonist binding | -8.5 | -4.7 | -6.6 |
| 5-HT3 Serotonin Receptor | human | Antagonist binding | -4.1 | 9.5 | 2.7 |
| Sigma Receptor (non-selective) | human | Agonist binding | 28 | 30.6 | 29.3 |
| Androgen Receptor | human | Agonist binding | -3.8 | -9 | -6.4 |
| SKCa Potassium Channel | rat | Antagonist binding | -2.5 | -4.7 | -3.6 |
| Na+ channel (site 2) | rat | Antagonist binding | 12 | -7.3 | 2.3 |
| Cl- channel (GABA-gated) | rat | Antagonist binding | 24.7 | 23.2 | 23.9 |
| Norepinephrine transporter | human | Antagonist binding | 4.8 | 18.1 | 11.5 |
| Dopamine transporter | human | Antagonist binding | 22.8 | 37.1 | 29.9 |
| GABA transporter | rat | Antagonist binding | -13.1 | -18.4 | -15.7 |
| 5-HT transporter | human | Antagonist binding | -6.2 | 4.6 | -0.8 |
| PDE5 (non-selective) Phosphodiesterase Enzyme | human | Enzyme inhibition | 54.6 | 59.4 | 57 |
| Acetylcholinesterase | human | Enzyme inhibition | -5.9 | -3.5 | -4.7 |
