# Supplementary figures and images for: Treatment with HC-070, a potent inhibitor of TRPC4 and TRPC5, leads to anxiolytic and antidepressant effects in mice
Source: PLoS One. 2018 Jan 31;13(1):e0191225. doi: 10.1371/journal.pone.0191225 (PMC5791972; doi:10.1371/journal.pone.0191225)

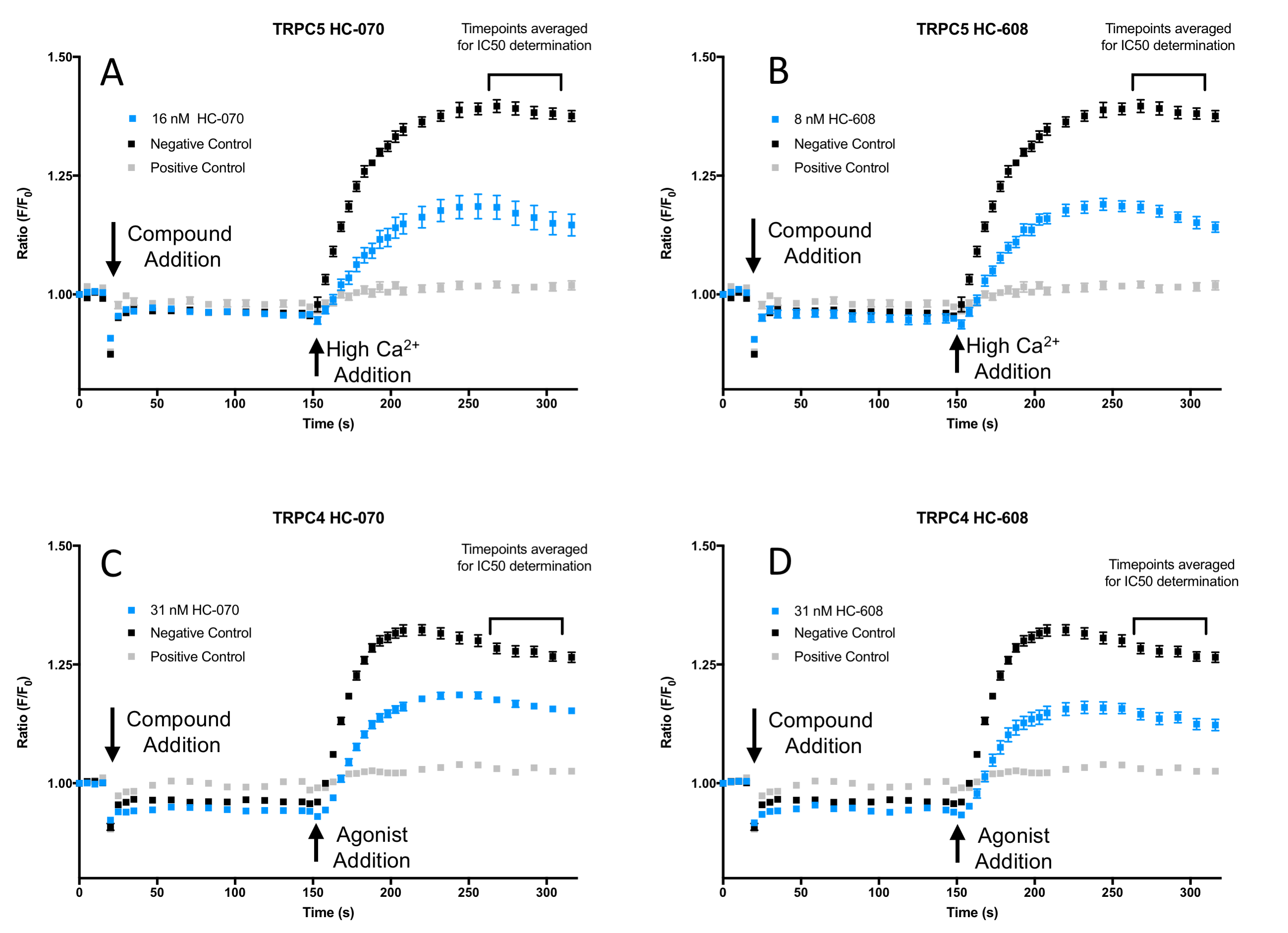

Supplement: S1 Fig — (A & B) Time course of cells expressing TRPC5 in the fluorometric assays. Cells plated into 384 black wall clear bottom plates were loaded with the fluorescent calcium indicator Fluo-4AM and the fluorescence intensity of each well was determined periodically over the course of ~5 minutes. The raw fluorescence in each well at each time point was divided by the initial fluorescence in each well. HC-070 (A) or HC-608 (B) (blue symbols) at the indicated concentration was added at the indicated time point and cells were incubated for ~2 minutes. High calcium buffer was added at the time point indicated and the responses monitored for ~2.5 minutes. Values at the indicated time points were averaged to determine the response to agonist. Negative control wells (black symbols) which received only the compound vehicle and assay buffer were included on each plate as were positive control wells (grey symbols) containing the 2-APB. (C & D) Time course of cells expressing TRPC4 in the fluorometric assays. Cells plated into 384 black wall clear bottom plates were loaded with the fluorescent calcium indicator Fluo-4AM and the fluorescence intensity of each well was determined periodically over the course of ~5 minutes. The raw fluorescence in each well at each time point was divided by the initial fluorescence in each well. HC-070 (C) or HC-608 (D) (blue symbols) at the indicated concentration was added at the indicated time point and cells were incubated for ~2 minutes. High calcium buffer containing Carbachol (7 μM final concentration) was added at the time point indicated and the responses monitored for ~2.5 minutes. Values at the indicated time points were averaged to determine the response to agonist. Negative control wells (black symbols) which received only the compound vehicle and assay buffer were included on each plate as were positive control wells (grey symbols) containing the 2-APB. (TIF) [file pone.0191225.s005.tif]

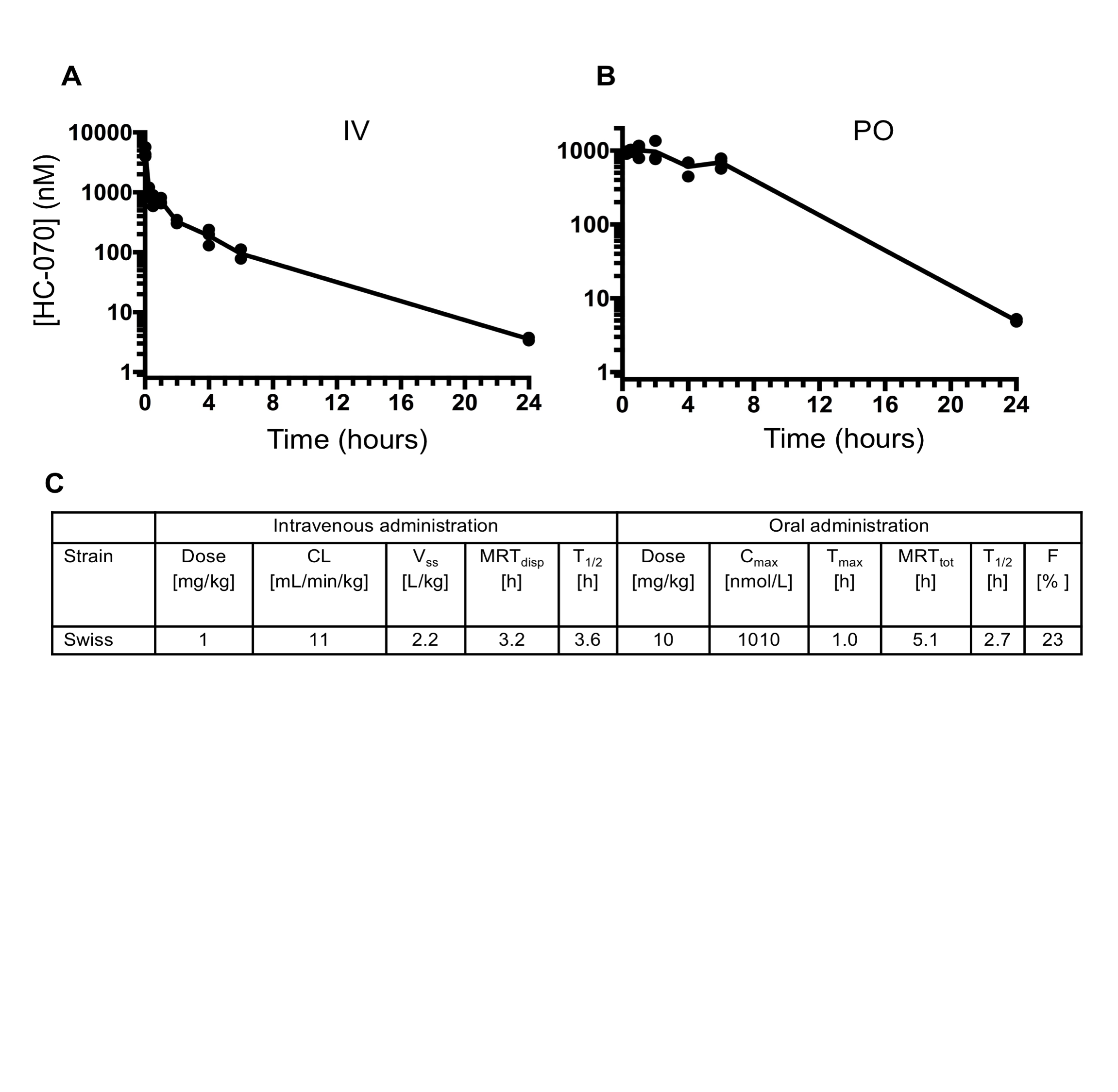

Supplement: S2 Fig — PK profiles of HC-070 after (A) intravenous and (B) oral administration in Swiss Webster mice. Plasma concentrations were determined by LC-MS/MS. Points represent the individual concentrations at the times indicated. Lines represent mean exposure (n = 12 mice/arm). (C) Summary of PK properties. CL = clearance; Vss = volume of distribution at steady state; MRTdisp = mean residence time of drug molecules after intravascular administration; T1/2 = half-life. (D) Plasma and brain concentrations measured 2 hours after intravenous or oral administration of 1 or 10 mg/kg HC-070, respectively. CPL = concentration in plasma, CBR = concentration in brain, KP,BR = partitioning coefficient between brain and plasma. (TIF) [file pone.0191225.s006.tif]

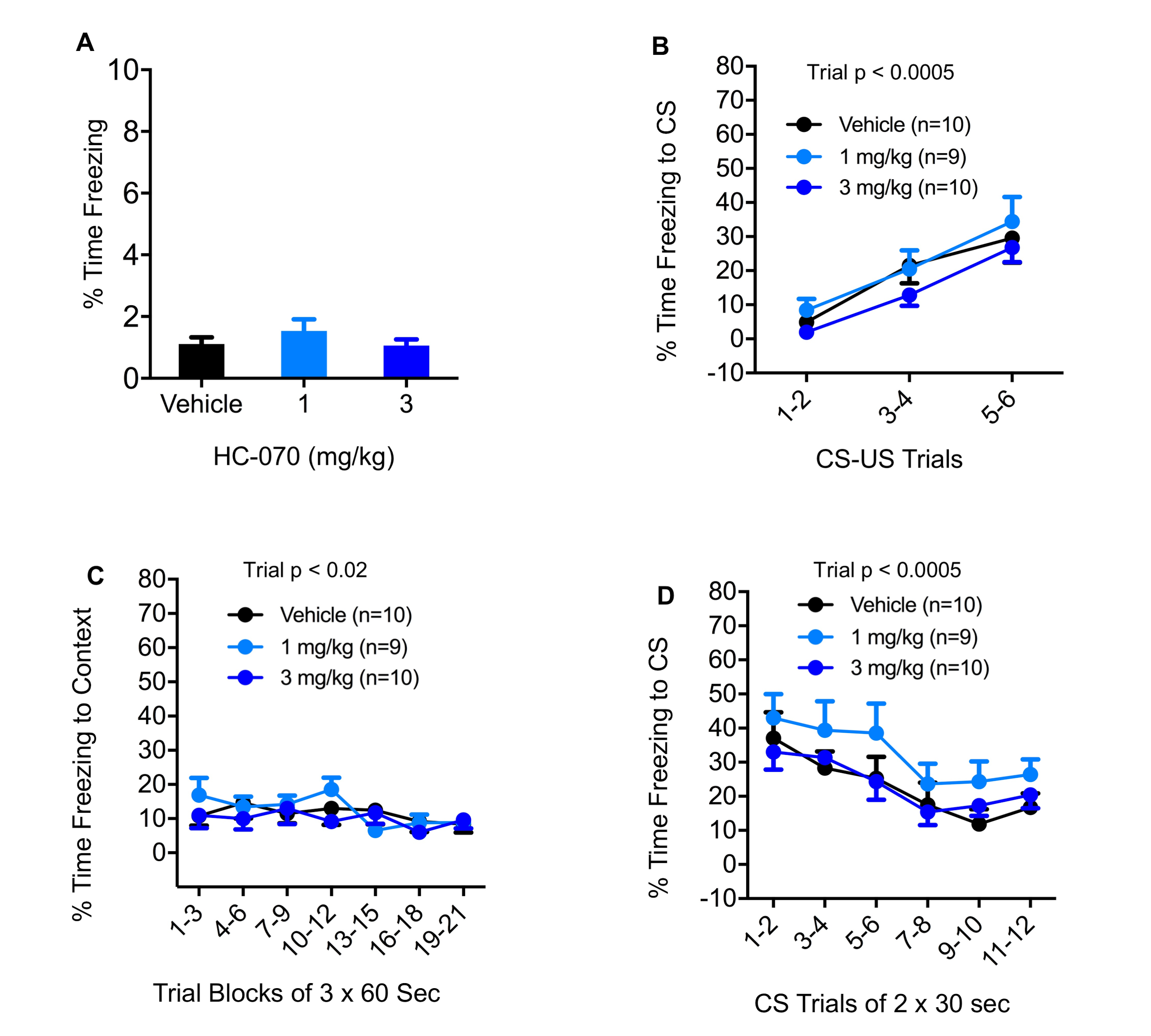

Supplement: S3 Fig — (A) Without drug administration, when placed in an unfamiliar arena (context), mice showed a low level of freezing, a fear behavior. (B) The next day, mice were administered vehicle, 1 or 3 mg/kg HC-070 PO and 2 hours later were placed in the same context, and exposed to a tone conditioned stimulus (CS) that announced an electroshock unconditioned stimulus (US). Mice acquired increased CS freezing across successive pairings of the CS and US, indicating the learning of fear of the CS, without an effect of HC-070. (C) The next day, mice received vehicle, 1 or 3 mg/kg HC-070 PO (same dose allocation as on previous day) and 2 hours later were placed in the same context in which CS-US conditioning took place the previous day. HC-070 was without effect on context fear memory, as indicated by each drug group showing low and similar freezing levels. (D) Immediately after the context memory test, mice were presented with the tone CS memory test. Freezing to the CS was higher than to the context, but there was still no effect of HC-070 on CS fear memory in these otherwise non-manipulated mice. (TIF) [file pone.0191225.s007.tif]

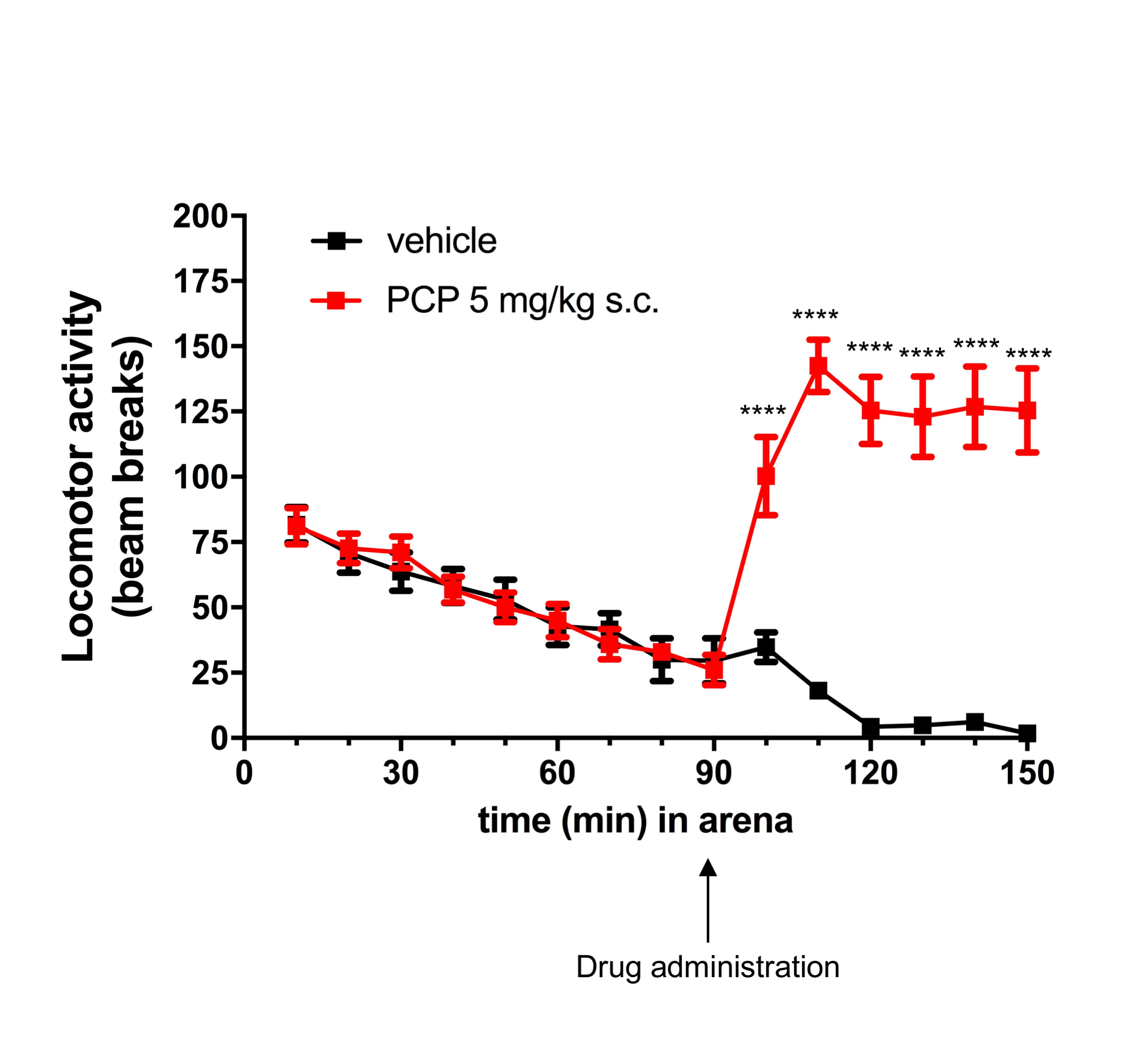

Supplement: S4 Fig — After 90 minutes of habituation to the chamber, mice were administered vehicle or 5 mg/kg PCP subcutaneously. Activity was recorded for another 60 minutes. PCP substantially increased activity, as expected. (2-way ANOVA followed by a Dunnett’s multiple comparison’s test, p<0.0001; n = 8/group). (TIF) [file pone.0191225.s008.tif]
