# Supplementary material for: Treatment with HC-070, a potent inhibitor of TRPC4 and TRPC5, leads to anxiolytic and antidepressant effects in mice
Source: PLoS One. 2018 Jan 31;13(1):e0191225. doi: 10.1371/journal.pone.0191225 (PMC5791972; doi:10.1371/journal.pone.0191225)
Supplement: S1 Report — (PDF) [file pone.0191225.s009.pdf]

FINAL REPORT

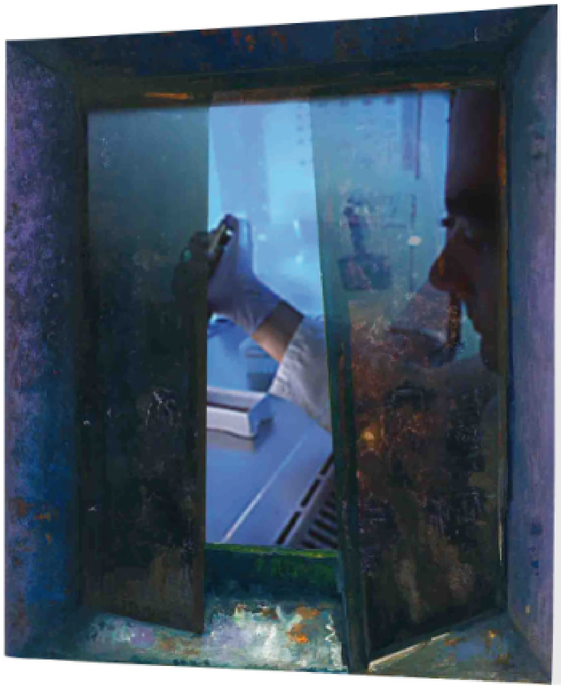

In Vitro Pharmacology

Study of HC-070

STUDY NUMBER
100005024

Annie OTTO-BRUC, Ph.D.

September 26, 2012

CONFIDENTIAL

STUDY REFERENCES

**Study title**	***In Vitro* Pharmacology**<br>**Study of HC-070**	
**Cerep study number**	**100005024**	**FINAL REPORT**<br>**September 26, 2012**
**Experimental period**	September 6, 2012 - September 25, 2012	
**Client personal study number**	-	
**PO number**		

PERSONS INVOLVED IN THE STUDY

**Investigator**	**Cerep**<br>Le Bois l'Evêque<br>B.P. 30001<br>86 600 Celle l'Evescault<br>France<br>Tel: +33 (0)5 49 89 30 00<br>Fax: +33 (0)5 49 43 21 70	**Annie OTTO-BRUC, Ph.D.**<br>Principal Scientist, Pharmacology<br><br>Tel: +33 (0)5 49 89 33 87<br>Fax: +33 (0)5 49 43 21 70
**Study sponsor**	**HYDRA BIOSCIENCES**<br>Target Discovery-Channel group<br>790 Memorial Drive<br><br>CAMBRIDGE, MA 02139<br>U.S.A.	**Dr. Magdalene MORAN**<br>Senior Scientist and Director
**Testing facilities**	**Cerep**<br>Le Bois l'Evêque<br>B.P. 30001<br>86 600 Celle l'Evescault<br>France	**Cerep**<br>15318 NE 95th Street<br>Redmond, WA 98052<br>U.S.A.

APPROVAL

**Investigator**	**Cerep**<br>Le Bois l'Evêque<br>B.P. 30001<br>86 600 Celle l'Evescault<br>France<br>Tel: +33 (0)5 49 89 30 00<br>Fax: +33 (0)5 49 43 21 70	**Annie OTTO-BRUC, Ph.D.**<br>Principal Scientist, Pharmacology<br><br>Tel: +33 (0)5 49 89 33 87<br>Fax: +33 (0)5 49 43 21 70<br><br>Date September 27, 2012<br><br>Signature
**Quality assurance statement**	Cerep's Quality Unit certifies that results presented in this report were generated using the materials and methods mentioned and that these results accurately reflect the raw data.<br><br>Date September 28, 2012<br><br>Signature<br><br>Nadine Pasquier.	

CONTENTS

1. SUMMARY 5

2. COMPOUNDS 6
 2.1. Test Compounds 6
 2.2. Reference Compounds 6

3. RESULTS 7
 3.1. *In Vitro* Pharmacology: Binding Assays 7
  3.1.1. Test Compound Results 7
  3.1.2. Reference Compound Results 10
 3.2. *In Vitro* Pharmacology: Enzyme and Uptake Assays 12
  3.2.1. Test Compound Results 12
  3.2.2. Reference Compound Results 12

4. RESULTS INTERPRETATION GUIDE 13

5. STORAGE AND RETENTION OF RECORDS 13

6. MATERIALS AND METHODS 14
 6.1. Experimental Conditions 14
  6.1.1. *In Vitro* Pharmacology: Binding Assays 14
  6.1.2. *In Vitro* Pharmacology: Enzyme and Uptake Assays 16
 6.2. Analysis and expression of results 17
  6.2.1. *In Vitro* Pharmacology: Binding Assays 17
  6.2.2. *In Vitro* Pharmacology: Enzyme and Uptake Assays 17

7. BIBLIOGRAPHY 19

1. SUMMARY

The purpose of this study was to test HC-070 in binding and enzyme and uptake assays.

2. COMPOUNDS

2.1. Test Compounds

From: **HYDRA BIOSCIENCES**

Client Compound ID	Cerep ID	Reference Number	Batch Number	FW	MW	Purity	Received Form	Stock solution	Flag
HC-070	100005024-1	-	2	475.0	475.0	-	Liquid	1.E-02 M DMSO*	-

FW: Formula Weight - MW: Molecular Weight

*: For *in vitro* pharmacology assays, depending on the assay volume and solvent tolerance, the stock solutions were diluted to [100x], [333x] or [1000x] in 100% solvent, then either added directly or further diluted to [10x] or [5x] in H_2O or assay buffer before addition to the assay well (final solvent concentration kept constant).

2.2. Reference Compounds

In each experiment and if applicable, the respective reference compound was tested concurrently with HC-070, and the data were compared with historical values determined at Cerep. The experiment was accepted in accordance with Cerep's validation Standard Operating Procedure.

3. RESULTS

3.1. *In Vitro* Pharmacology: Binding Assays

3.1.1. Test Compound Results

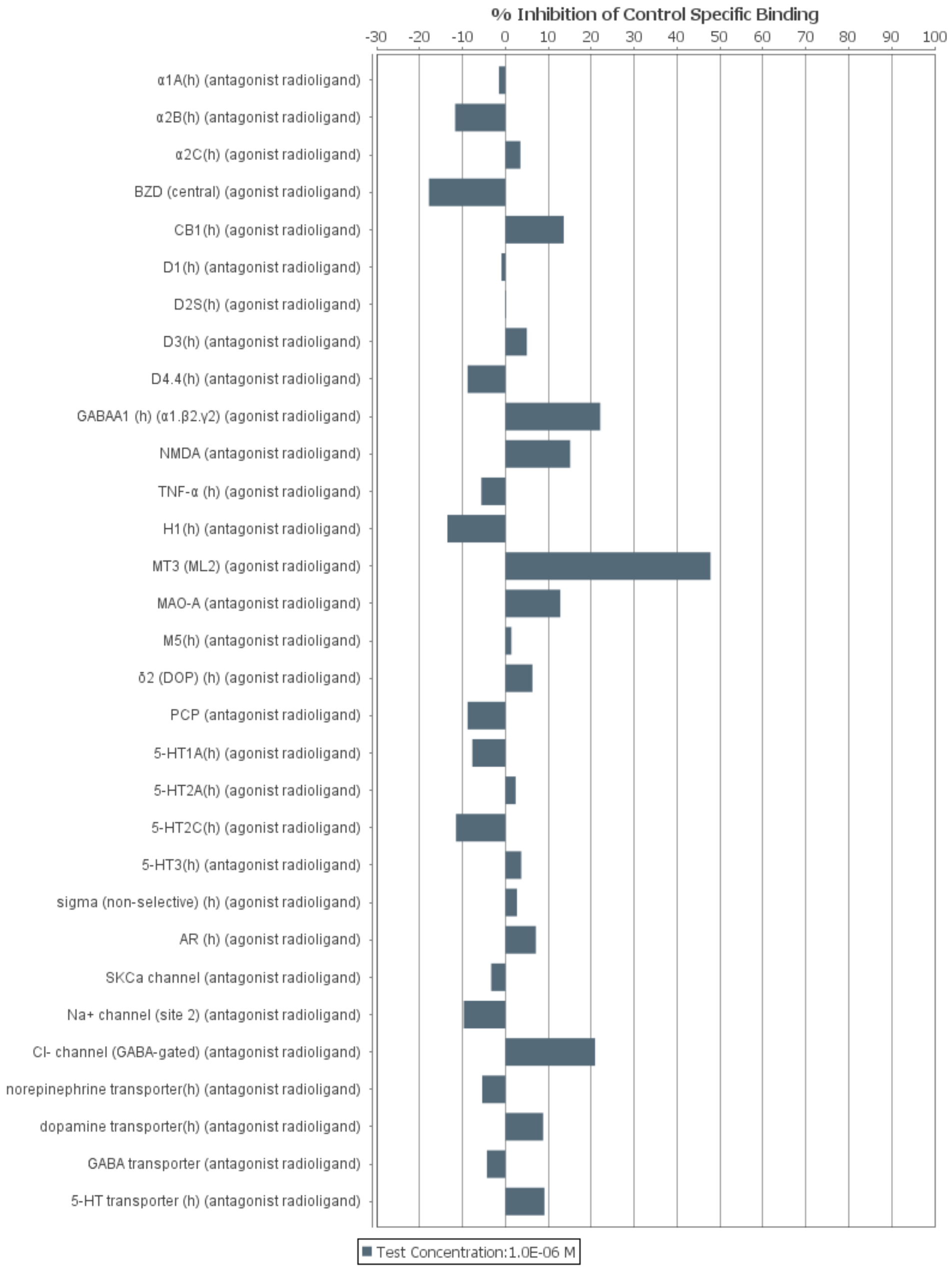

Figure 1. Histogram for HC-070

Cerep Compound I.D.	Client Compound I.D.	Test Concentration	% Inhibition of Control Specific Binding		
			1st	2nd	Mean
**α_{1A} *(h)* (antagonist radioligand)**					
100005024-1	HC-070	1.0E-06 M	-6.1	3.1	-1.5
**α_{2B} *(h)* (antagonist radioligand)**					
100005024-1	HC-070	1.0E-06 M	-22.3	-1.2	-11.7
**α_{2C} *(h)* (agonist radioligand)**					
100005024-1	HC-070	1.0E-06 M	7.9	-1.0	3.5
**BZD (central) (agonist radioligand)**					
100005024-1	HC-070	1.0E-06 M	-26.0	-9.6	-17.8
**CB_1 *(h)* (agonist radioligand)**					
100005024-1	HC-070	1.0E-06 M	8.4	18.8	13.6
**D_1 *(h)* (antagonist radioligand)**					
100005024-1	HC-070	1.0E-06 M	3.0	-4.8	-0.9
**D_{2S} *(h)* (agonist radioligand)**					
100005024-1	HC-070	1.0E-06 M	-3.0	3.4	0.2
**D_3 *(h)* (antagonist radioligand)**					
100005024-1	HC-070	1.0E-06 M	3.6	6.4	5.0
**$D_{4.4}$ *(h)* (antagonist radioligand)**					
100005024-1	HC-070	1.0E-06 M	-15.8	-1.8	-8.8
**$GABA_{A1}$ *(h)* (α1,β2,γ2) (agonist radioligand)**					
100005024-1	HC-070	1.0E-06 M	27.9	16.4	22.1
**NMDA (antagonist radioligand)**					
100005024-1	HC-070	1.0E-06 M	4.4	25.7	15.1
**TNF-α *(h)* (agonist radioligand)**					
100005024-1	HC-070	1.0E-06 M	-5.4	-5.8	-5.6
**H_1 *(h)* (antagonist radioligand)**					
100005024-1	HC-070	1.0E-06 M	-15.6	-11.4	-13.5
**MT_3 (ML_2) (agonist radioligand)**					
100005024-1	HC-070	1.0E-06 M	46.1	49.5	47.8
**MAO-A (antagonist radioligand)**					
100005024-1	HC-070	1.0E-06 M	12.6	13.1	12.8
**M_5 *(h)* (antagonist radioligand)**					
100005024-1	HC-070	1.0E-06 M	-7.3	10.1	1.4
**δ_2 (DOP) *(h)* (agonist radioligand)**					
100005024-1	HC-070	1.0E-06 M	6.8	5.9	6.3
**PCP (antagonist radioligand)**					
100005024-1	HC-070	1.0E-06 M	-7.6	-10.0	-8.8
**$5\text{-}HT_{1A}$ *(h)* (agonist radioligand)**					
100005024-1	HC-070	1.0E-06 M	8.7	-24.2	-7.7
**$5\text{-}HT_{2A}$ *(h)* (agonist radioligand)**					
100005024-1	HC-070	1.0E-06 M	-1.3	6.1	2.4
**$5\text{-}HT_{2C}$ *(h)* (agonist radioligand)**					
100005024-1	HC-070	1.0E-06 M	-13.4	-9.5	-11.5
**$5\text{-}HT_3$ *(h)* (antagonist radioligand)**					
100005024-1	HC-070	1.0E-06 M	4.7	2.6	3.7
**sigma (non-selective) (h) (agonist radioligand)**					
100005024-1	HC-070	1.0E-06 M	0.3	5.1	2.7
**AR *(h)* (agonist radioligand)**					
100005024-1	HC-070	1.0E-06 M	11.1	3.2	7.1
**SK_{Ca} channel (antagonist radioligand)**					
100005024-1	HC-070	1.0E-06 M	-10.1	3.5	-3.3
**Na^+ channel (site 2) (antagonist radioligand)**					
100005024-1	HC-070	1.0E-06 M	-6.9	-12.5	-9.7

Cerep Compound I.D.	Client Compound I.D.	Test Concentration	% Inhibition of Control Specific Binding		
			1st	2nd	Mean
**Cl^- channel (GABA-gated) (antagonist radioligand)**					
100005024-1	HC-070	1.0E-06 M	24.2	17.5	20.9
**norepinephrine transporter *(h)* (antagonist radioligand)**					
100005024-1	HC-070	1.0E-06 M	-10.1	-0.6	-5.4
**dopamine transporter *(h)* (antagonist radioligand)**					
100005024-1	HC-070	1.0E-06 M	8.9	8.6	8.8
**GABA transporter (antagonist radioligand)**					
100005024-1	HC-070	1.0E-06 M	-3.4	-5.2	-4.3
**5-HT transporter *(h)* (antagonist radioligand)**					
100005024-1	HC-070	1.0E-06 M	-0.3	18.4	9.1

3.1.2. Reference Compound Results

Reference Compound	IC_{50} (M)	K_i (M)	nH
**α_{1A} *(h)* (antagonist radioligand)**			
WB 4101	2.2E-10 M	1.1E-10 M	1.4
**α_{2B} *(h)* (antagonist radioligand)**			
yohimbine	5.1E-09 M	3.4E-09 M	1.0
**α_{2C} *(h)* (agonist radioligand)**			
epinephrine	4.8E-09 M	3.0E-09 M	1.1
**BZD (central) (agonist radioligand)**			
diazepam	8.4E-09 M	7.1E-09 M	0.9
**CB_1 *(h)* (agonist radioligand)**			
CP 55940	4.0E-10 M	3.5E-10 M	0.8
**D_1 *(h)* (antagonist radioligand)**			
SCH 23390	2.6E-10 M	1.0E-10 M	1.0
**D_{2S} *(h)* (agonist radioligand)**			
7-OH-DPAT	3.5E-09 M	1.4E-09 M	0.8
**D_3 *(h)* (antagonist radioligand)**			
(+)butaclamol	2.9E-09 M	6.4E-10 M	1.3
**$D_{4.4}$ *(h)* (antagonist radioligand)**			
clozapine	5.6E-08 M	2.2E-08 M	1.2
**$GABA_{A1}$ *(h)* (α1,β2,γ2) (agonist radioligand)**			
muscimol	1.0E-07 M	6.7E-08 M	0.8
**NMDA (antagonist radioligand)**			
CGS 19755	1.5E-06 M	1.2E-06 M	0.8
**TNF-α *(h)* (agonist radioligand)**			
TNF-alpha	1.3E-10 M	4.5E-11 M	0.9
**H_1 *(h)* (antagonist radioligand)**			
pyrilamine	1.6E-09 M	9.9E-10 M	0.9
**MT_3 (ML_2) (agonist radioligand)**			
melatonin	3.8E-08 M	3.7E-08 M	0.8
**MAO-A (antagonist radioligand)**			
clorgyline	1.2E-09 M	7.1E-10 M	1.6
**M_5 *(h)* (antagonist radioligand)**			
4-DAMP	7.6E-10 M	3.8E-10 M	0.8
**δ_2 (DOP) *(h)* (agonist radioligand)**			
DPDPE	3.7E-09 M	2.2E-09 M	1.0
**PCP (antagonist radioligand)**			
MK 801	7.9E-09 M	4.5E-09 M	1.0
**$5\text{-}HT_{1A}$ *(h)* (agonist radioligand)**			
8-OH-DPAT	7.4E-10 M	4.6E-10 M	0.9
**$5\text{-}HT_{2A}$ *(h)* (agonist radioligand)**			
(±)DOI	2.3E-10 M	1.7E-10 M	0.8
**$5\text{-}HT_{2C}$ *(h)* (agonist radioligand)**			
(±)DOI	9.8E-10 M	8.8E-10 M	0.8
**$5\text{-}HT_3$ *(h)* (antagonist radioligand)**			
MDL 72222	7.9E-09 M	5.5E-09 M	1.1
**sigma (non-selective) (h) (agonist radioligand)**			
haloperidol	3.3E-08 M	2.6E-08 M	0.6
**AR *(h)* (agonist radioligand)**			
mibolerone	2.8E-09 M	1.2E-09 M	1.1
**SK_{Ca} channel (antagonist radioligand)**			
apamin	9.9E-12 M	5.0E-12 M	1.1

Reference Compound	IC_{50} (M)	K_i (M)	nH
**Na^+ channel (site 2) (antagonist radioligand)**			
veratridine	9.4E-06 M	8.4E-06 M	0.9
**Cl^- channel (GABA-gated) (antagonist radioligand)**			
picrotoxinin	1.3E-07 M	1.1E-07 M	0.9
**norepinephrine transporter *(h)* (antagonist radioligand)**			
protriptyline	3.3E-09 M	2.5E-09 M	1.3
**dopamine transporter *(h)* (antagonist radioligand)**			
BTCP	7.4E-09 M	3.9E-09 M	1.2
**GABA transporter (antagonist radioligand)**			
nipecotic acid	2.0E-06 M	2.0E-06 M	0.8
**5-HT transporter *(h)* (antagonist radioligand)**			
imipramine	1.6E-09 M	7.1E-10 M	0.9

3.2. *In Vitro* Pharmacology: Enzyme and Uptake Assays

3.2.1. Test Compound Results

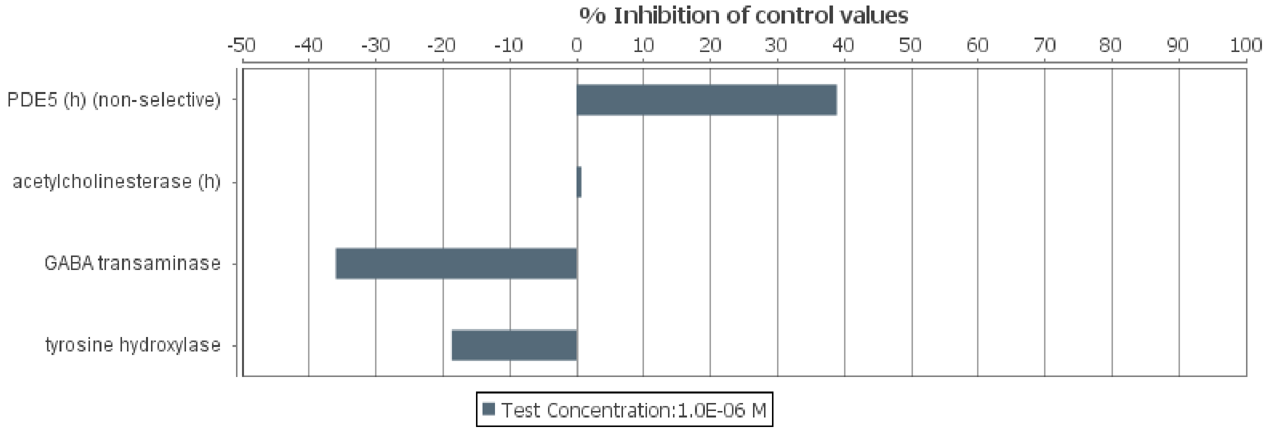

Figure 2. Histogram for HC-123070

Cerep Compound I.D.	Client Compound I.D.	Test Concentration	% Inhibition of control values 1st	2nd	Mean
**PDE5 *(h)* (non-selective)**					
100005024-1	HC-123	1.0E-06 M	27.3	50.4	38.9
**acetylcholinesterase *(h)***					
100005024-1	HC-070	1.0E-06 M	2.1	-0.7	0.7
**GABA transaminase**					
100005024-1	HC-070	1.0E-06 M	-33.1	-38.8	-36.0
**tyrosine hydroxylase**					
100005024-1	HC-070	1.0E-06 M	-27.2	-10.1	-18.7

3.2.2. Reference Compound Results

Reference Compound	IC_{50} (M)	nH
**PDE5 *(h)* (non-selective)**		
dipyridamole	9.0E-07 M	1.7
**acetylcholinesterase *(h)***		
neostigmine	2.7E-08 M	1.2
**GABA transaminase**		
AoAA	1.3E-07 M	1.7
**tyrosine hydroxylase**		
3-iodo L-tyrosine	6.1E-07 M	1.0

4. RESULTS INTERPRETATION GUIDE

In Vitro Pharmacology

Results showing an inhibition (or stimulation for assays run in basal conditions) higher than 50% are considered to represent significant effects of the test compounds. 50% is the most common cut-off value for further investigation (determination of IC_{50} or EC_{50} values from concentration-response curves) that we would recommend.
Results showing an inhibition (or stimulation) between 25% and 50% are indicative of weak to moderate effects (in most assays, they should be confirmed by further testing as they are within a range where more inter-experimental variability can occur).
Results showing an inhibition (or stimulation) lower than 25% are not considered significant and mostly attributable to variability of the signal around the control level.
Low to moderate negative values have no real meaning and are attributable to variability of the signal around the control level. High negative values (≥ 50%) that are sometimes obtained with high concentrations of test compounds are generally attributable to non-specific effects of the test compounds in the assays. On rare occasion they could suggest an allosteric effect of the test compound.

5. STORAGE AND RETENTION OF RECORDS

All documents generated during the performance of the study (raw data, various records such as QA audit reports, an original copy of the study report, study plan…) will be archived by Cerep for a 10-year period after study completion. The access to the archives is restricted to authorized employees only.

The original final report provided to the sponsor will be kept by the sponsor under its sole responsibility.

6. MATERIALS AND METHODS

6.1. Experimental Conditions

6.1.1. *In Vitro* Pharmacology: Binding Assays

Assay	Source	Ligand	Conc.	Kd	Non Specific	Incubation	Detection Method	Bibl.
**Receptors**								
**α_{1A} *(h)* (antagonist radioligand)**	human recombinant (CHO cells)	[^{3}H]prazosin	0.1 nM	0.1 nM	epinephrine (0.1 mM)	60 min RT	Scintillation counting	897
**α_{2B} *(h)* (antagonist radioligand)**	human recombinant (CHO cells)	[^{3}H]RX 821002	2.5 nM	5 nM	(-)epinephrine (100 µM)	60 min RT	Scintillation counting	56
**α_{2C} *(h)* (agonist radioligand)**	human recombinant (CHO cells)	[^{3}H] UK 14.304	3 nM	5 nM	RX 821002	60 min RT	Scintillation counting	1126
**CB_1 *(h)* (agonist radioligand)**	human recombinant (CHO cells)	[^{3}H]CP 55940	0.5 nM	3.5 nM	WIN 55212-2 (10 µM)	120 min 37°C	Scintillation counting	657
**D_1 *(h)* (antagonist radioligand)**	human recombinant (CHO cells)	[^{3}H]SCH 23390	0.3 nM	0.2 nM	SCH 23390 (1 µM)	60 min RT	Scintillation counting	281
**D_{2S} *(h)* (agonist radioligand)**	human recombinant (HEK-293 cells)	[^{3}H]7-OH-DPAT	1 nM	0.68 nM	butaclamol (10 µM)	60 min RT	Scintillation counting	87
**D_3 *(h)* (antagonist radioligand)**	human recombinant (CHO cells)	[^{3}H]methyl-spiperone	0.3 nM	0.085 nM	(+)butaclamol (10 µM)	60 min RT	Scintillation counting	145
**$D_{4.4}$ *(h)* (antagonist radioligand)**	human recombinant (CHO cells)	[^{3}H]methyl-spiperone	0.3 nM	0.19 nM	(+)butaclamol (10 µM)	60 min RT	Scintillation counting	252
**$GABA_{A1}$ *(h)* (α1,β2,γ2) (agonist radioligand)**	human recombinant (CHO cells)	[^{3}H]muscimol	15 nM	30 nM	muscimol (10 µM)	120 min RT	Scintillation counting	1096
**TNF-α *(h)* (agonist radioligand)**	U-937 cells	[^{125}I]TNF-α	0.1 nM	0.05 nM	TNF-α (10 nM)	120 min 4°C	Scintillation counting	26
**H_1 *(h)* (antagonist radioligand)**	human recombinant (HEK-293 cells)	[^{3}H]pyrilamine	1 nM	1.7 nM	pyrilamine (1 µM)	60 min RT	Scintillation counting	492
**MT_3 (ML_2) (agonist radioligand)**	hamster brain	[^{125}I]2-iodomelatonin	0.1 nM	4.8 nM	melatonin (30 µM)	60 min 4°C	Scintillation counting	186
**M_5 *(h)* (antagonist radioligand)**	human recombinant (CHO cells)	[^{3}H]4-DAMP	0.3 nM	0.3 nM	atropine (1 µM)	60 min RT	Scintillation counting	59
**δ_2 (DOP) *(h)* (agonist radioligand)**	human recombinant (CHO cells)	[^{3}H]DADLE	0.5 nM	0.73 nM	naltrexone (10 µM)	120 min RT	Scintillation counting	501
**$5\text{-}HT_{1A}$ *(h)* (agonist radioligand)**	human recombinant (HEK-293 cells)	[^{3}H]8-OH-DPAT	0.3 nM	0.5 nM	8-OH-DPAT (10 µM)	60 min RT	Scintillation counting	164
**$5\text{-}HT_{2A}$ *(h)* (agonist radioligand)**	human recombinant (HEK-293 cells)	[^{125}I](±)DOI	0.1 nM	0.3 nM	(±)DOI (1 µM)	60 min RT	Scintillation counting	288

Assay	Source	Ligand	Conc.	Kd	Non Specific	Incubation	Detection Method	Bibl.
**$5\text{-}HT_{2C}$ *(h)* (agonist radioligand)**	human recombinant (HEK-293 cells)	[^{125}I](±)DOI	0.1 nM	0.9 nM	(±)DOI (10 µM)	60 min 37°C	Scintillation counting	288
**sigma (non-selective) (h) (agonist radioligand)**	cellules Jurkat (endogène)	[3H]DTG	10 nM	41 nM	Haloperidol (10 µM)	120 min RT	Scintillation counting	1136
**AR *(h)* (agonist radioligand)**	LNCaP cells (cytosol)	[^{3}H]methyltrienolone	1 nM	0.8 nM	mibolerone (1 µM)	24 hr 4°C	Scintillation counting	498
**Ion channels**								
**BZD (central) (agonist radioligand)**	rat cerebral cortex	[^{3}H]flunitrazepam	0.4 nM	2.1 nM	diazepam (3 µM)	60 min 4°C	Scintillation counting	227
**NMDA (antagonist radioligand)**	rat cerebral cortex	[^{3}H]CGP 39653	5 nM	23 nM	L-glutamate (100 µM)	60 min 4°C	Scintillation counting	221
**PCP (antagonist radioligand)**	rat cerebral cortex	[^{3}H]TCP	10 nM	13 nM	MK 801 (10 µM)	120 min 37°C	Scintillation counting	257
**$5\text{-}HT_3$ *(h)* (antagonist radioligand)**	human recombinant (CHO cells)	[^{3}H]BRL 43694	0.5 nM	1.15 nM	MDL 72222 (10 µM)	120 min RT	Scintillation counting	109
**SK_{Ca} channel (antagonist radioligand)**	rat cerebral cortex	[^{125}I]apamin	0.007 nM	0.007 nM	apamin (100 nM)	60 min 4°C	Scintillation counting	112
**Na^+ channel (site 2) (antagonist radioligand)**	rat cerebral cortex	[^{3}H]batrachotoxinin	10 nM	91 nM	veratridine (300 µM)	60 min 37°C	Scintillation counting	28
**Cl^- channel (GABA-gated) (antagonist radioligand)**	rat cerebral cortex	[^{35}S]TBPS	3 nM	14.6 nM	picrotoxinin (20 µM)	120 min RT	Scintillation counting	136
**Transporters**								
**norepinephrine transporter *(h)* (antagonist radioligand)**	human recombinant (CHO cells)	[^{3}H]nisoxetine	1 nM	2.9 nM	desipramine (1 µM)	120 min 4°C	Scintillation counting	180
**dopamine transporter *(h)* (antagonist radioligand)**	human recombinant (CHO cells)	[^{3}H]BTCP	4 nM	4.5 nM	BTCP (10 µM)	120 min 4°C	Scintillation counting	190
**GABA transporter (antagonist radioligand)**	rat cerebral cortex	[^{3}H]GABA (+ 10 µM isoguvacine) (+ 10 µM baclofen)	10 nM	4600 nM	GABA (1 mM)	30 min RT	Scintillation counting	214
**5-HT transporter *(h)* (antagonist radioligand)**	human recombinant (CHO cells)	[^{3}H]imipramine	2 nM	1.7 nM	imipramine (10 µM)	60 min RT	Scintillation counting	566
**Other enzymes**								
**MAO-A (antagonist radioligand)**	rat cerebral cortex	[^{3}H]Ro 41-1049	10 nM	14 nM	clorgyline (1 µM)	60 min 37°C	Scintillation counting	36

6.1.2. *In Vitro* Pharmacology: Enzyme and Uptake Assays

Assay	Source	Substrate/ Stimulus/Tracer	Incubation	Measured Component	Detection Method	Bibl.
**Other enzymes**						
**PDE5 *(h)* (non-selective)**	human platelets	[^{3}H]cGMP + cGMP (1 µM)	60 min RT	[^{3}H]5'GMP	Scintillation counting	263
**acetylcholinesterase *(h)***	human recombinant (HEK-293 cells)	AMTCh (400 µM)	30 min RT	5 thio 2 nitrobenzoic acid	Photometry	63
**GABA transaminase**	rat brain	GABA (9 mM) + α-ketoglutarate (9 mM)	60 min 37°C	succinic semialdehyde	Fluorimetry	286
**tyrosine hydroxylase**	rat striatum	[^{3}H]tyrosine (10 µM)	40 min 37°C	[^{3}H]H_2O	Scintillation counting	168

6.2. Analysis and expression of results

6.2.1. *In Vitro* Pharmacology: Binding Assays

The results are expressed as a percent of control specific binding

$$\frac{\text{measured specific binding}}{\text{control specific binding}} * 100$$

and as a percent inhibition of control specific binding

$$100 - \left(\frac{\text{measured specific binding}}{\text{control specific binding}} * 100\right)$$

obtained in the presence of HC-070.

REDACTED

6.2.2. *In Vitro* Pharmacology: Enzyme and Uptake Assays

The results are expressed as a percent of control specific activity

$$\frac{\text{measured specific activity}}{\text{control specific activity}} * 100$$

and as a percent inhibition of control specific activity

$$100 - \left(\frac{\text{measured specific activity}}{\text{control specific activity}} * 100\right)$$

obtained in the presence of HC-070.

REDACTED

7. BIBLIOGRAPHY

26. Brockhaus, M. et al. (1990), *Proc. Natl. Acad. Sci. U.S.A.*, 87 *: 3127-3131.*
28. Brown, G.B. (1986), *J. Neurosci.*, 6 *: 2064-2070.*
36. Cesura, A.M. et al. (1990), *Mol. Pharmacol.*, 37 *: 358-366.*
56. Devedjian, J.C. et al. (1994), *Eur. J. Pharmacol.*, 252 *: 43-49.*
59. Dorje, F. et al. (1991), *J. Pharmacol. Exp. Ther.*, 256 *: 727-733.*
63. Ellman, G.L. et al. (1961), *Biochem. Pharmacol.*, 7 *: 88-95.*
87. Grandy, D.K. et al. (1989), *Proc. Natl. Acad. Sci. U.S.A.*, 86 *: 9762-9766.*
109. Hope, A.G. et al. (1996), *Brit. J. Pharmacol.*, 118 *: 1237-1245.*
112. Hugues, M. et al. (1982), *J. Biol. Chem.*, 257 *: 2762-2769.*
136. Lewin, A.H. et al. (1989), *Mol. Pharmacol.*, 35 *: 189-194.*
145. Mackenzie, R.G. et al. (1994), *Eur. J. Pharmacol.*, 266 *: 79-85.*
164. Mulheron, J.G. et al. (1994), *J. Biol. Chem.*, 269 *: 12954-12962.*
168. Nagatsu, T. et al. (1964), *Anal. Biochem.*, 9 *: 122-126.*
180. Pacholczyk, T. et al. (1991), *Nature*, 350 *: 350-354.*
186. Pickering, D.S. and Niles, L.P. (1990), *Eur. J. Pharmacol.*, 175 *: 71-77.*
190. Pristupa, Z.B. et al. (1994), *Mol. Pharmacol.*, 45 *: 125-135.*
214. Shank, R.P. et al. (1990), *J. Neurochem.*, 54 *: 2007-2015.*
221. Sills, M.A. et al. (1991), *Eur. J. Pharmacol.*, 192 *: 19-24.*
227. Speth, R.C. et al. (1979), *Life Sci.*, 24 *: 351-358.*
252. Van Tol, H.H.M. et al. (1992), *Nature*, 358 *: 149-152.*
257. Vignon, J. et al. (1986), *Brain Res.*, 378 *: 133-141.*
263. Weishaar, R.E. et al. (1986), *Biochem. Pharmacol.*, 35 *: 787-800.*
281. Zhou, Q.Y. et al. (1990), *Nature*, 347 *: 76-80.*
286. Losher, W. (1981), *J. Neurochem.*, 36 *: 1521-1527.*
288. Bryant, H.U. et al. (1996), *Life Sci.*, 15 *: 1259-1268.*
492. Smit, M.J. et al. (1996), *Brit. J. Pharmacol.*, 117 *: 1071-1080.*
498. Zava, D.T. et al. (1979), *Endocrinology*, 104 *: 1007-1012.*
501. Simonin, F. et al. (1994), *Mol. Pharmacol.*, 46 *: 1015-1021.*
566. Tatsumi, M. et al. (1999), *Eur. J. Pharmacol.*, 368 *: 277-283.*
657. Rinaldi-Carmona, M. et al. (1996), *J. Pharmacol. Exp. Ther.*, 278 *: 871-878.*
897. Schwinn, D.A. et al. (1990), *J. Biol. Chem.*, 265 *: 8183-8189.*
1096. Wang, X.K. (2001), *Acta. Pharmacol. Sin.*, 22 *: 521-523.*
1126. Hoeren, M. et al. (2008), *Nauny-Schmiedeberg's Arch Pharmacol*, 378 *:17-26*
1136. GANAPATHY ME. et al. (1999), *JPET*, 289 *: 251-260*
