# Supplementary material for: Treatment with HC-070, a potent inhibitor of TRPC4 and TRPC5, leads to anxiolytic and antidepressant effects in mice
Source: PLoS One. 2018 Jan 31;13(1):e0191225. doi: 10.1371/journal.pone.0191225 (PMC5791972; doi:10.1371/journal.pone.0191225)
Supplement: S2 Report — (PDF) [file pone.0191225.s010.pdf]

FINAL REPORT

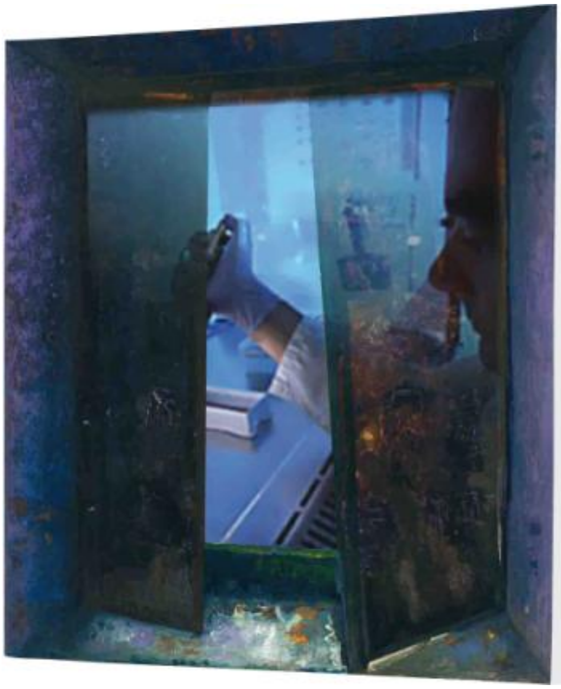

In Vitro Pharmacology

Study of HC-122608

STUDY NUMBER
100004002

Annie OTTO-BRUC, Ph.D.

September 6, 2012

CONFIDENTIAL

STUDY REFERENCES

**Study title**	***In Vitro* Pharmacology**<br>**Study of HC-122608**	
**Cerep study number**	**100004002**	**FINAL REPORT**<br>**September 6, 2012**
**Experimental period**	August 10, 2012 -August 31, 2012	
**Client personal study number**	-	
**PO number**	HBS14359	

PERSONS INVOLVED IN THE STUDY

**Investigator**	**Cerep**<br>Le Bois l'Evêque<br>B.P. 30001<br>86 600 Celle l'Evescault<br>France<br>Tel: +33 (0)5 49 89 30 00<br>Fax: +33 (0)5 49 43 21 70	**Annie OTTO-BRUC, Ph.D.**<br>Principal Scientist, Pharmacology<br><br>Tel: +33 (0)5 49 89 33 87<br>Fax: +33 (0)5 49 43 21 70
**Study sponsor**	**HYDRA BIOSCIENCES**<br>Target Discovery-Channel group<br>790 Memorial Drive<br><br>CAMBRIDGE, MA 02139<br>U.S.A.	**Dr. Magdalene MORAN**<br>Senior Scientist and Director
**Testing facilities**	**Cerep**<br>Le Bois l'Evêque<br>B.P. 30001<br>86 600 Celle l'Evescault<br>France	**Cerep**<br>15318 NE 95th Street<br>Redmond, WA 98052<br>U.S.A.

APPROVAL

**Investigator**	**Cerep**<br>Le Bois l'Evêque<br>B.P. 30001<br>86 600 Celle l'Evescault<br>France<br>Tel: +33 (0)5 49 89 30 00<br>Fax: +33 (0)5 49 43 21 70	**Annie OTTO-BRUC, Ph.D.**<br>Principal Scientist, Pharmacology<br><br>Tel: +33 (0)5 49 89 33 87<br>Fax: +33 (0)5 49 43 21 70

Date September 6, 2012

Signature

Quality assurance statement

Cerep's Quality Unit certifies that results presented in this report were generated using the materials and methods mentioned and that these results accurately reflect the raw data.

Date Septembre 07, 2012

Signature

Nadine Pasquier.

CONTENTS

1. SUMMARY **5**

2. COMPOUNDS **6**

2.1. Test Compounds **6**

2.2. Reference Compounds **6**

3. RESULTS **7**

3.1. *In Vitro* Pharmacology: Binding Assays **7**

3.1.1. Test Compound Results 7

3.1.2. Reference Compound Results 13

3.2. *In Vitro* Pharmacology: Enzyme and Uptake Assays **17**

3.2.1. Test Compound Results 17

3.2.2. Reference Compound Results 18

4. RESULTS INTERPRETATION GUIDE **20**

5. STORAGE AND RETENTION OF RECORDS **20**

6. MATERIALS AND METHODS **21**

6.1. Experimental Conditions 21

6.1.1. *In Vitro* Pharmacology: Binding Assays 21

6.1.2. *In Vitro* Pharmacology: Enzyme and Uptake Assays 26

6.2. Analysis and expression of results 27

6.2.1. *In Vitro* Pharmacology: Binding Assays **27**

6.2.2. *In Vitro* Pharmacology: Enzyme and Uptake Assays **27**

7. BIBLIOGRAPHY **29**

1. SUMMARY

The purpose of this study was to test HC-122608 in binding and enzyme assays.

2. COMPOUNDS

2.1. Test Compounds

From: **HYDRA BIOSCIENCES**

Client Compound ID	Cerep ID	Reference Number	Batch Number	FW	MW	Purity	Received Form	Stock solution	Flag
HC-122608	100004002-1	-	5	525.0	525.0	-	Liquid	1.E-02 M DMSO*	-

FW: Formula Weight - MW: Molecular Weight

*: For *in vitro* pharmacology assays, depending on the assay volume and solvent tolerance, the stock solutions were diluted to [100x], [333x] or [1000x] in 100% solvent, then either added directly or further diluted to [10x] or [5x] in H_2O or assay buffer before addition to the assay well (final solvent concentration kept constant).

2.2. Reference Compounds

In each experiment and if applicable, the respective reference compound was tested concurrently with HC-122608, and the data were compared with historical values determined at Cerep. The experiment was accepted in accordance with Cerep's validation Standard Operating Procedure.

3. RESULTS

3.1. *In Vitro* Pharmacology: Binding Assays

3.1.1. Test Compound Results

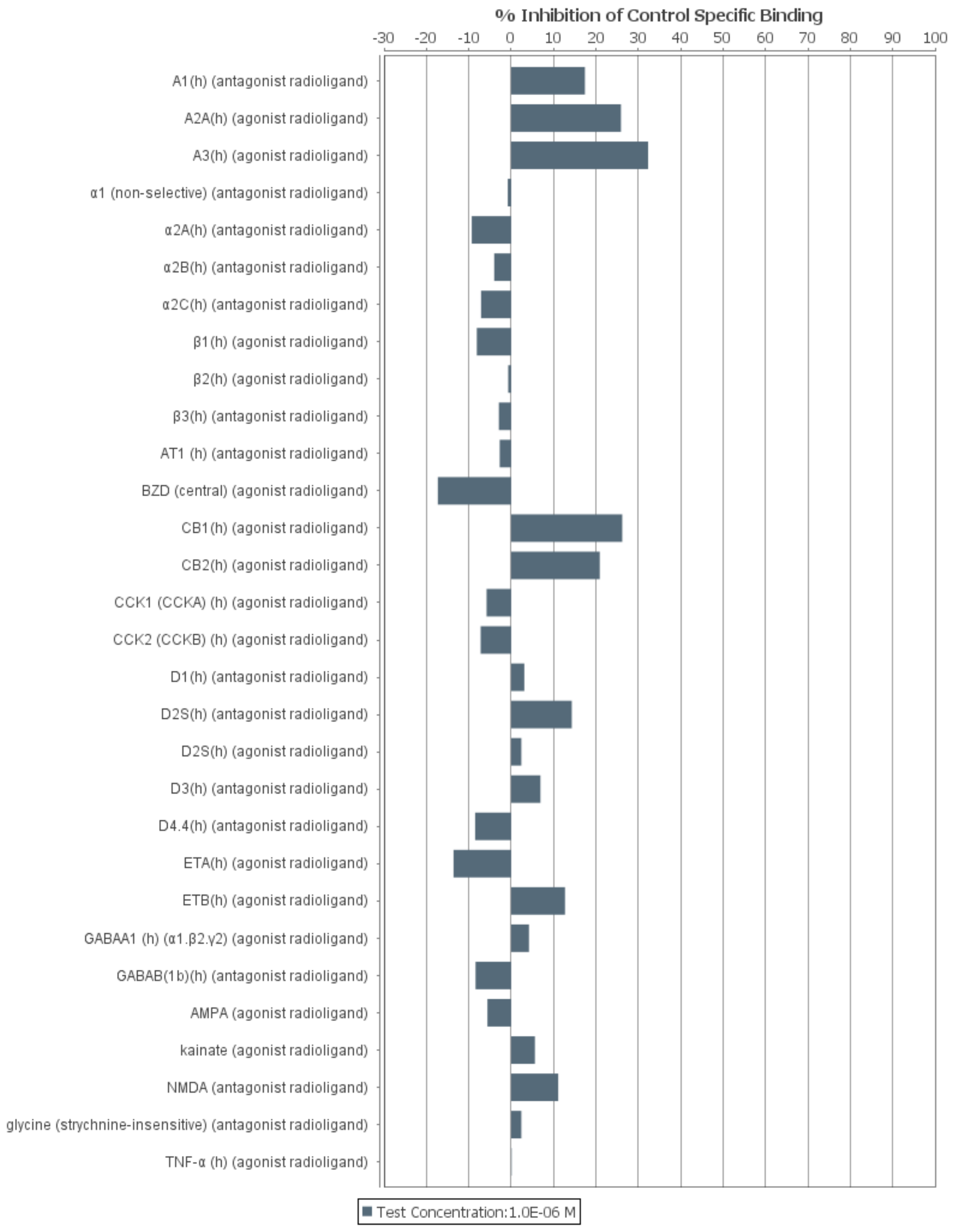

Figure 1. Histogram for HC-122608 [1/3]

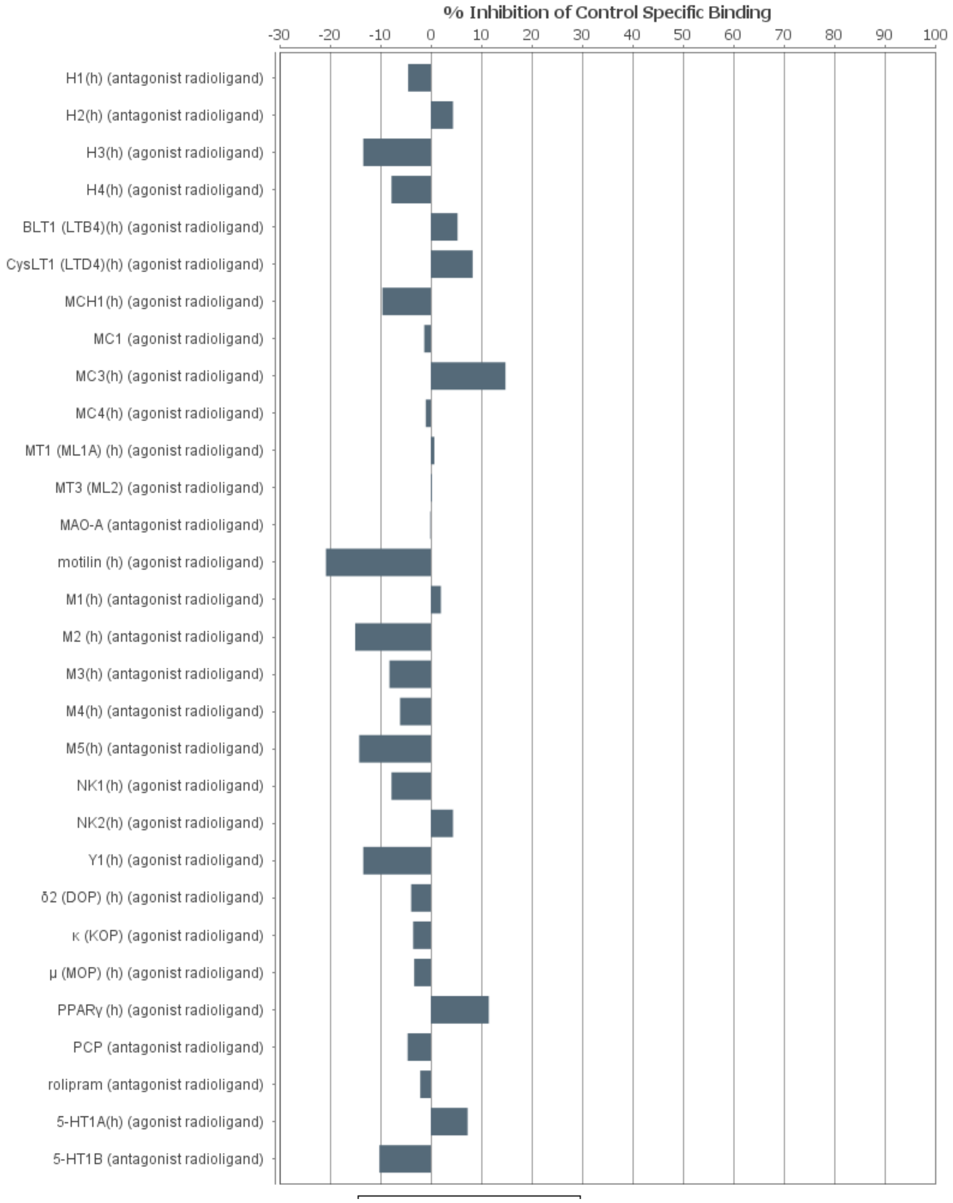

Figure 2. Histogram for HC-122608 [2/3]

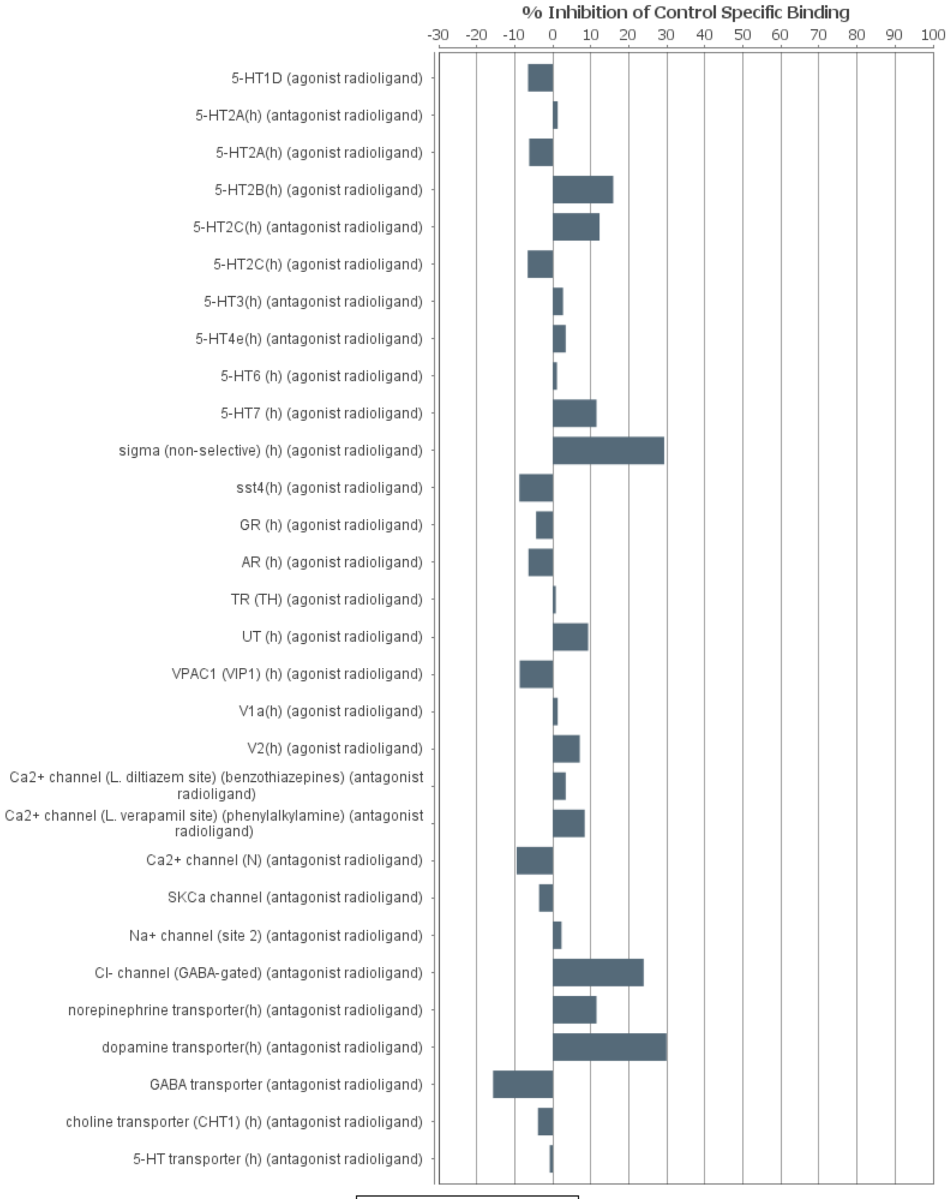

Figure 3. Histogram for HC-122608 [3/3]

Cerep Compound I.D.	Client Compound I.D.	Test Concentration	% Inhibition of Control Specific Binding 1st	2nd	Mean
**A_1 *(h)* (antagonist radioligand)**					
100004002-1	HC-122608	1.0E-06 M	22.5	12.3	17.4
**A_{2A} *(h)* (agonist radioligand)**					
100004002-1	HC-122608	1.0E-06 M	20.8	31.1	25.9
**A_3 *(h)* (agonist radioligand)**					
100004002-1	HC-122608	1.0E-06 M	28.3	36.4	32.3
**α_1 (non-selective) (antagonist radioligand)**					
100004002-1	HC-122608	1.0E-06 M	-2.8	1.1	-0.8
**α_{2A} *(h)* (antagonist radioligand)**					
100004002-1	HC-122608	1.0E-06 M	-14.9	-3.7	-9.3
**α_{2B} *(h)* (antagonist radioligand)**					
100004002-1	HC-122608	1.0E-06 M	-7.4	-0.7	-4.0

Cerep Compound I.D.	Client Compound I.D.	Test Concentration	% Inhibition of Control Specific Binding		
			1st	2nd	Mean
**α_{2C} *(h)* (antagonist radioligand)**					
100004002-1	HC-122608	1.0E-06 M	-4.1	-10.2	-7.1
**β_1 *(h)* (agonist radioligand)**					
100004002-1	HC-122608	1.0E-06 M	-14.5	-1.8	-8.1
**β_2 *(h)* (agonist radioligand)**					
100004002-1	HC-122608	1.0E-06 M	-1.9	0.5	-0.7
**β_3 *(h)* (antagonist radioligand)**					
100004002-1	HC-122608	1.0E-06 M	0.9	-6.7	-2.9
**AT_1 *(h)* (antagonist radioligand)**					
100004002-1	HC-122608	1.0E-06 M	0.5	-6.0	-2.7
**BZD (central) (agonist radioligand)**					
100004002-1	HC-122608	1.0E-06 M	-3.0	-31.5	-17.3
**CB_1 *(h)* (agonist radioligand)**					
100004002-1	HC-122608	1.0E-06 M	27.0	25.5	26.2
**CB_2 *(h)* (agonist radioligand)**					
100004002-1	HC-122608	1.0E-06 M	16.6	25.1	20.9
**CCK_1 (CCK_A) *(h)* (agonist radioligand)**					
100004002-1	HC-122608	1.0E-06 M	-11.2	-0.4	-5.8
**CCK_2 (CCK_B) *(h)* (agonist radioligand)**					
100004002-1	HC-122608	1.0E-06 M	-13.8	-0.7	-7.2
**D_1 *(h)* (antagonist radioligand)**					
100004002-1	HC-122608	1.0E-06 M	1.1	5.0	3.1
**D_{2S} *(h)* (antagonist radioligand)**					
100004002-1	HC-122608	1.0E-06 M	19.2	9.4	14.3
**D_{2S} *(h)* (agonist radioligand)**					
100004002-1	HC-122608	1.0E-06 M	4.6	0.2	2.4
**D_3 *(h)* (antagonist radioligand)**					
100004002-1	HC-122608	1.0E-06 M	4.0	9.7	6.9
**$D_{4.4}$ *(h)* (antagonist radioligand)**					
100004002-1	HC-122608	1.0E-06 M	-20.4	3.4	-8.5
**ET_A *(h)* (agonist radioligand)**					
100004002-1	HC-122608	1.0E-06 M	-33.9	6.7	-13.6
**ET_B *(h)* (agonist radioligand)**					
100004002-1	HC-122608	1.0E-06 M	25.5	-0.1	12.7
**$GABA_{A1}$ *(h)* (α1,β2,γ2) (agonist radioligand)**					
100004002-1	HC-122608	1.0E-06 M	0.6	7.9	4.2
**$GABA_{B(1b)}$ *(h)* (antagonist radioligand)**					
100004002-1	HC-122608	1.0E-06 M	-9.0	-7.8	-8.4
**AMPA (agonist radioligand)**					
100004002-1	HC-122608	1.0E-06 M	-2.1	-9.2	-5.6
**kainate (agonist radioligand)**					
100004002-1	HC-122608	1.0E-06 M	5.9	5.3	5.6
**NMDA (antagonist radioligand)**					
100004002-1	HC-122608	1.0E-06 M	19.0	3.2	11.1
**glycine (strychnine-insensitive) (antagonist radioligand)**					
100004002-1	HC-122608	1.0E-06 M	1.7	3.1	2.4
**TNF-α *(h)* (agonist radioligand)**					
100004002-1	HC-122608	1.0E-06 M	8.8	-8.6	0.1
**H_1 *(h)* (antagonist radioligand)**					
100004002-1	HC-122608	1.0E-06 M	-8.1	-0.9	-4.5
**H_2 *(h)* (antagonist radioligand)**					
100004002-1	HC-122608	1.0E-06 M	1.1	7.7	4.4
**H_3 *(h)* (agonist radioligand)**					
100004002-1	HC-122608	1.0E-06 M	-25.9	-0.8	-13.4
**H_4 *(h)* (agonist radioligand)**					
100004002-1	HC-122608	1.0E-06 M	-13.6	-1.9	-7.8

Cerep Compound I.D.	Client Compound I.D.	Test Concentration	% Inhibition of Control Specific Binding		
			1st	2nd	Mean
**BLT_1 (LTB_4) *(h)* (agonist radioligand)**					
100004002-1	HC-122608	1.0E-06 M	-2.9	13.4	5.3
**$CysLT_1$ (LTD_4) *(h)* (agonist radioligand)**					
100004002-1	HC-122608	1.0E-06 M	13.2	3.4	8.3
**MCH_1 *(h)* (agonist radioligand)**					
100004002-1	HC-122608	1.0E-06 M	7.9	-27.2	-9.6
**MC_1 (agonist radioligand)**					
100004002-1	HC-122608	1.0E-06 M	1.0	-3.6	-1.3
**MC_3 *(h)* (agonist radioligand)**					
100004002-1	HC-122608	1.0E-06 M	14.4	15.3	14.8
**MC_4 *(h)* (agonist radioligand)**					
100004002-1	HC-122608	1.0E-06 M	2.9	-4.8	-1.0
**MT_1 (ML_{1A}) *(h)* (agonist radioligand)**					
100004002-1	HC-122608	1.0E-06 M	2.2	-0.9	0.7
**MT_3 (ML_2) (agonist radioligand)**					
100004002-1	HC-122608	1.0E-06 M	-0.7	1.2	0.2
**MAO-A (antagonist radioligand)**					
100004002-1	HC-122608	1.0E-06 M	-3.8	3.6	-0.1
**motilin *(h)* (agonist radioligand)**					
100004002-1	HC-122608	1.0E-06 M	-25.4	-16.2	-20.8
**M_1 *(h)* (antagonist radioligand)**					
100004002-1	HC-122608	1.0E-06 M	-1.6	5.5	2.0
**M_2 *(h)* (antagonist radioligand)**					
100004002-1	HC-122608	1.0E-06 M	-15.9	-14.2	-15.0
**M_3 *(h)* (antagonist radioligand)**					
100004002-1	HC-122608	1.0E-06 M	-9.0	-7.5	-8.2
**M_4 *(h)* (antagonist radioligand)**					
100004002-1	HC-122608	1.0E-06 M	-16.1	3.9	-6.1
**M_5 *(h)* (antagonist radioligand)**					
100004002-1	HC-122608	1.0E-06 M	-15.6	-12.8	-14.2
**NK_1 *(h)* (agonist radioligand)**					
100004002-1	HC-122608	1.0E-06 M	-2.6	-13.0	-7.8
**NK_2 *(h)* (agonist radioligand)**					
100004002-1	HC-122608	1.0E-06 M	-10.0	18.9	4.4
**Y_1 *(h)* (agonist radioligand)**					
100004002-1	HC-122608	1.0E-06 M	-12.8	-14.1	-13.4
**δ_2 (DOP) *(h)* (agonist radioligand)**					
100004002-1	HC-122608	1.0E-06 M	-10.8	3.1	-3.9
**κ (KOP) (agonist radioligand)**					
100004002-1	HC-122608	1.0E-06 M	-8.1	1.1	-3.5
**μ (MOP) *(h)* (agonist radioligand)**					
100004002-1	HC-122608	1.0E-06 M	0.1	-6.8	-3.3
**PPARγ *(h)* (agonist radioligand)**					
100004002-1	HC-122608	1.0E-06 M	6.7	16.2	11.5
**PCP (antagonist radioligand)**					
100004002-1	HC-122608	1.0E-06 M	-9.0	-0.1	-4.6
**rolipram (antagonist radioligand)**					
100004002-1	HC-122608	1.0E-06 M	-4.6	0.5	-2.1
**$5\text{-}HT_{1A}$ *(h)* (agonist radioligand)**					
100004002-1	HC-122608	1.0E-06 M	-0.2	14.8	7.3
**$5\text{-}HT_{1B}$ (antagonist radioligand)**					
100004002-1	HC-122608	1.0E-06 M	-11.0	-9.4	-10.2
**$5\text{-}HT_{1D}$ (agonist radioligand)**					
100004002-1	HC-122608	1.0E-06 M	-4.2	-8.8	-6.5
**$5\text{-}HT_{2A}$ *(h)* (antagonist radioligand)**					
100004002-1	HC-122608	1.0E-06 M	-4.3	6.9	1.3

Cerep Compound I.D.	Client Compound I.D.	Test Concentration	% Inhibition of Control Specific Binding		
			1st	2nd	Mean
**5-HT_{2A} *(h)* (agonist radioligand)**					
100004002-1	HC-122608	1.0E-06 M	1.8	-14.3	-6.2
**5-HT_{2B} *(h)* (agonist radioligand)**					
100004002-1	HC-122608	1.0E-06 M	15.9	15.8	15.9
**5-HT_{2C} *(h)* (antagonist radioligand)**					
100004002-1	HC-122608	1.0E-06 M	12.1	12.6	12.3
**5-HT_{2C} *(h)* (agonist radioligand)**					
100004002-1	HC-122608	1.0E-06 M	-8.5	-4.7	-6.6
**5-HT_3 *(h)* (antagonist radioligand)**					
100004002-1	HC-122608	1.0E-06 M	-4.1	9.5	2.7
**5-HT_{4e} *(h)* (antagonist radioligand)**					
100004002-1	HC-122608	1.0E-06 M	-3.7	10.6	3.4
**5-HT_6 *(h)* (agonist radioligand)**					
100004002-1	HC-122608	1.0E-06 M	-5.1	7.3	1.1
**5-HT_7 *(h)* (agonist radioligand)**					
100004002-1	HC-122608	1.0E-06 M	12.1	10.9	11.5
**sigma (non-selective) (h) (agonist radioligand)**					
100004002-1	HC-122608	1.0E-06 M	28.0	30.6	29.3
**sst_4 *(h)* (agonist radioligand)**					
100004002-1	HC-122608	1.0E-06 M	-27.0	9.5	-8.8
**GR *(h)* (agonist radioligand)**					
100004002-1	HC-122608	1.0E-06 M	-5.0	-3.8	-4.4
**AR *(h)* (agonist radioligand)**					
100004002-1	HC-122608	1.0E-06 M	-3.8	-9.0	-6.4
**TR (TH) (agonist radioligand)**					
100004002-1	HC-122608	1.0E-06 M	3.2	-1.6	0.8
**UT *(h)* (agonist radioligand)**					
100004002-1	HC-122608	1.0E-06 M	11.5	7.0	9.3
**$VPAC_1$ (VIP_1) *(h)* (agonist radioligand)**					
100004002-1	HC-122608	1.0E-06 M	-9.0	-8.4	-8.7
**V_{1a} *(h)* (agonist radioligand)**					
100004002-1	HC-122608	1.0E-06 M	-9.9	12.5	1.3
**V_2 *(h)* (agonist radioligand)**					
100004002-1	HC-122608	1.0E-06 M	8.6	5.7	7.1
**Ca^{2+} channel (L, diltiazem site) (benzothiazepines) (antagonist radioligand)**					
100004002-1	HC-122608	1.0E-06 M	-1.9	8.8	3.4
**Ca^{2+} channel (L, verapamil site) (phenylalkylamine) (antagonist radioligand)**					
100004002-1	HC-122608	1.0E-06 M	2.3	14.5	8.4
**Ca^{2+} channel (N) (antagonist radioligand)**					
100004002-1	HC-122608	1.0E-06 M	-5.9	-13.1	-9.5
**SK_{Ca} channel (antagonist radioligand)**					
100004002-1	HC-122608	1.0E-06 M	-2.5	-4.7	-3.6
**Na^+ channel (site 2) (antagonist radioligand)**					
100004002-1	HC-122608	1.0E-06 M	12.0	-7.3	2.3
**Cl^- channel (GABA-gated) (antagonist radioligand)**					
100004002-1	HC-122608	1.0E-06 M	24.7	23.2	23.9
**norepinephrine transporter *(h)* (antagonist radioligand)**					
100004002-1	HC-122608	1.0E-06 M	4.8	18.1	11.5
**dopamine transporter *(h)* (antagonist radioligand)**					
100004002-1	HC-122608	1.0E-06 M	22.8	37.1	29.9
**GABA transporter (antagonist radioligand)**					
100004002-1	HC-122608	1.0E-06 M	-13.1	-18.4	-15.7
**choline transporter (CHT1) *(h)* (antagonist radioligand)**					
100004002-1	HC-122608	1.0E-06 M	-5.8	-1.9	-3.9
**5-HT transporter *(h)* (antagonist radioligand)**					
100004002-1	HC-122608	1.0E-06 M	-6.2	4.6	-0.8

3.1.2. Reference Compound Results

Reference Compound	IC_{50} (M)	K_i (M)	nH
**A_1 *(h)* (antagonist radioligand)**			
DPCPX	7.8E-10 M	4.9E-10 M	1.2
**A_{2A} *(h)* (agonist radioligand)**			
NECA	1.7E-08 M	1.4E-08 M	0.6
**A_3 *(h)* (agonist radioligand)**			
IB-MECA	1.8E-10 M	1.1E-10 M	0.8
**α_1 (non-selective) (antagonist radioligand)**			
prazosin	2.1E-10 M	5.5E-11 M	1.1
**α_{2A} *(h)* (antagonist radioligand)**			
yohimbine	3.4E-09 M	1.5E-09 M	1.1
**α_{2B} *(h)* (antagonist radioligand)**			
yohimbine	5.9E-09 M	3.9E-09 M	1.0
**α_{2C} *(h)* (antagonist radioligand)**			
yohimbine	2.3E-09 M	7.4E-10 M	1.1
**β_1 *(h)* (agonist radioligand)**			
atenolol	1.5E-07 M	8.6E-08 M	1.0
**β_2 *(h)* (agonist radioligand)**			
ICI 118551	5.2E-10 M	1.7E-10 M	0.9
**β_3 *(h)* (antagonist radioligand)**			
cyanopindolol	5.9E-08 M	3.6E-08 M	0.5
**AT_1 *(h)* (antagonist radioligand)**			
saralasin	1.2E-09 M	6.2E-10 M	0.9
**BZD (central) (agonist radioligand)**			
diazepam	8.3E-09 M	6.9E-09 M	0.8
**CB_1 *(h)* (agonist radioligand)**			
CP 55940	5.0E-10 M	4.4E-10 M	0.7
**CB_2 *(h)* (agonist radioligand)**			
WIN 55212-2	3.2E-10 M	2.1E-10 M	0.5
**CCK_1 (CCK_A) *(h)* (agonist radioligand)**			
CCK-8s	8.2E-11 M	6.2E-11 M	1.0
**CCK_2 (CCK_B) *(h)* (agonist radioligand)**			
CCK-8s	1.1E-10 M	4.5E-11 M	0.8
**D_1 *(h)* (antagonist radioligand)**			
SCH 23390	2.4E-10 M	9.5E-11 M	0.7
**D_{2S} *(h)* (antagonist radioligand)**			
(+)butaclamol	2.5E-09 M	8.4E-10 M	1.1
**D_{2S} *(h)* (agonist radioligand)**			
7-OH-DPAT	1.7E-09 M	6.8E-10 M	0.9
**D_3 *(h)* (antagonist radioligand)**			
(+)butaclamol	1.3E-09 M	3.0E-10 M	0.8
**$D_{4.4}$ *(h)* (antagonist radioligand)**			
clozapine	4.6E-08 M	1.8E-08 M	0.9
**ET_A *(h)* (agonist radioligand)**			
endothelin-1	6.6E-11 M	3.3E-11 M	1.3
**ET_B *(h)* (agonist radioligand)**			
endothelin-3	1.1E-11 M	6.4E-12 M	1.0
**$GABA_{A1}$ *(h)* (α1,β2,γ2) (agonist radioligand)**			
muscimol	8.0E-08 M	5.3E-08 M	0.8
**$GABA_{B(1b)}$ *(h)* (antagonist radioligand)**			
CGP 54626	8.2E-10 M	4.1E-10 M	0.9
**AMPA (agonist radioligand)**			
L-glutamate	3.1E-07 M	2.8E-07 M	0.9
**kainate (agonist radioligand)**			
kainic acid	2.0E-08 M	1.6E-08 M	0.7
**NMDA (antagonist radioligand)**			
CGS 19755	2.0E-06 M	1.6E-06 M	1.4

Reference Compound	IC_{50} (M)	K_i (M)	nH
**glycine (strychnine-insensitive) (antagonist radioligand)**			
glycine	2.6E-07 M	2.4E-07 M	0.6
**TNF-α *(h)* (agonist radioligand)**			
TNF-alpha	1.3E-10 M	4.4E-11 M	0.8
**H_1 *(h)* (antagonist radioligand)**			
pyrilamine	2.3E-09 M	1.5E-09 M	1.2
**H_2 *(h)* (antagonist radioligand)**			
cimetidine	3.3E-07 M	3.2E-07 M	0.9
**H_3 *(h)* (agonist radioligand)**			
(R)α-Me-histamine	2.2E-09 M	5.3E-10 M	0.9
**H_4 *(h)* (agonist radioligand)**			
imetit	4.4E-09 M	1.9E-09 M	1.0
**BLT_1 (LTB_4) *(h)* (agonist radioligand)**			
LTB_4	2.2E-10 M	1.1E-10 M	0.8
**$CysLT_1$ (LTD_4) *(h)* (agonist radioligand)**			
LTD_4	2.7E-10 M	1.2E-10 M	1.1
**MCH_1 *(h)* (agonist radioligand)**			
human MCH	7.6E-11 M	6.9E-11 M	0.9
**MC_1 (agonist radioligand)**			
NDP-α-MSH	1.0E-10 M	5.1E-11 M	1.1
**MC_3 *(h)* (agonist radioligand)**			
NDP-α-MSH	7.3E-11 M	6.2E-11 M	0.6
**MC_4 *(h)* (agonist radioligand)**			
NDP-α-MSH	4.0E-10 M	3.7E-10 M	0.8
**MT_1 (ML_{1A}) *(h)* (agonist radioligand)**			
melatonin	1.8E-10 M	1.4E-10 M	1.0
**MT_3 (ML_2) (agonist radioligand)**			
melatonin	2.6E-07 M	2.5E-07 M	0.3
**MAO-A (antagonist radioligand)**			
clorgyline	1.0E-09 M	6.0E-10 M	1.5
**motilin *(h)* (agonist radioligand)**			
$[Nleu^{13}]$-motilin	2.8E-09 M	2.3E-09 M	0.7
**M_1 *(h)* (antagonist radioligand)**			
pirenzepine	1.7E-08 M	1.5E-08 M	0.8
**M_2 *(h)* (antagonist radioligand)**			
methoctramine	3.0E-08 M	2.1E-08 M	0.9
**M_3 *(h)* (antagonist radioligand)**			
4-DAMP	5.7E-10 M	4.1E-10 M	1.2
**M_4 *(h)* (antagonist radioligand)**			
4-DAMP	3.5E-10 M	2.1E-10 M	0.8
**M_5 *(h)* (antagonist radioligand)**			
4-DAMP	4.5E-10 M	2.2E-10 M	0.8
**NK_1 *(h)* (agonist radioligand)**			
$[Sar^9,Met(O_2)^{11}]$-SP	2.2E-10 M	9.8E-11 M	1.1
**NK_2 *(h)* (agonist radioligand)**			
$[Nleu^{10}]$-NKA (4-10)	5.8E-09 M	3.2E-09 M	0.8
**Y_1 *(h)* (agonist radioligand)**			
NPY	1.4E-10 M	9.6E-11 M	0.9
**$δ_2$ (DOP) *(h)* (agonist radioligand)**			
DPDPE	4.0E-09 M	2.4E-09 M	0.9
**κ (KOP) (agonist radioligand)**			
U 50488	8.1E-10 M	5.4E-10 M	1.1
**μ (MOP) *(h)* (agonist radioligand)**			
DAMGO	1.0E-09 M	4.1E-10 M	0.9
**PPARγ *(h)* (agonist radioligand)**			
rosiglitazone	1.1E-08 M	5.8E-09 M	0.9
**PCP (antagonist radioligand)**			
MK 801	6.2E-09 M	3.5E-09 M	1.2

Reference Compound	IC_{50} (M)	K_i (M)	nH
**rolipram (antagonist radioligand)**			
rolipram	7.1E-10 M	3.6E-10 M	0.7
**$5\text{-}HT_{1A}$ *(h)* (agonist radioligand)**			
8-OH-DPAT	4.3E-10 M	2.7E-10 M	1.1
**$5\text{-}HT_{1B}$ (antagonist radioligand)**			
serotonin	6.9E-09 M	4.2E-09 M	0.7
**$5\text{-}HT_{1D}$ (agonist radioligand)**			
serotonin	1.4E-09 M	4.7E-10 M	1.1
**$5\text{-}HT_{2A}$ *(h)* (antagonist radioligand)**			
ketanserin	1.2E-09 M	6.6E-10 M	1.4
**$5\text{-}HT_{2A}$ *(h)* (agonist radioligand)**			
(±)DOI	1.5E-10 M	1.2E-10 M	0.6
**$5\text{-}HT_{2B}$ *(h)* (agonist radioligand)**			
(±)DOI	5.5E-09 M	2.7E-09 M	1.1
**$5\text{-}HT_{2C}$ *(h)* (antagonist radioligand)**			
RS 102221	2.3E-09 M	7.8E-10 M	0.8
**$5\text{-}HT_{2C}$ *(h)* (agonist radioligand)**			
(±)DOI	6.8E-10 M	6.1E-10 M	1.0
**$5\text{-}HT_3$ *(h)* (antagonist radioligand)**			
MDL 72222	1.0E-08 M	7.1E-09 M	1.1
**$5\text{-}HT_{4e}$ *(h)* (antagonist radioligand)**			
serotonin	5.0E-07 M	1.7E-07 M	0.9
**$5\text{-}HT_6$ *(h)* (agonist radioligand)**			
serotonin	9.3E-08 M	4.4E-08 M	1.0
**$5\text{-}HT_7$ *(h)* (agonist radioligand)**			
serotonin	3.2E-10 M	1.2E-10 M	1.1
**sigma (non-selective) (h) (agonist radioligand)**			
haloperidol	5.4E-08 M	4.3E-08 M	0.8
**sst_4 *(h)* (agonist radioligand)**			
somatostatin-14	3.5E-09 M	3.5E-09 M	0.9
**GR *(h)* (agonist radioligand)**			
dexamethasone	4.1E-09 M	2.1E-09 M	1.1
**AR *(h)* (agonist radioligand)**			
mibolerone	2.6E-09 M	1.1E-09 M	1.0
**TR (TH) (agonist radioligand)**			
T_3	2.1E-10 M	1.5E-10 M	1.0
**UT *(h)* (agonist radioligand)**			
urotensin-II	1.2E-09 M	8.9E-10 M	1.0
**$VPAC_1$ (VIP_1) *(h)* (agonist radioligand)**			
VIP	2.3E-10 M	1.3E-10 M	0.9
**V_{1a} *(h)* (agonist radioligand)**			
$[d(CH_2)_5{}^1,Tyr(Me)_2]$-AVP	5.1E-10 M	3.2E-10 M	0.9
**V_2 *(h)* (agonist radioligand)**			
AVP	1.2E-09 M	8.8E-10 M	0.7
**Ca^{2+} channel (L, diltiazem site) (benzothiazepines) (antagonist radioligand)**			
diltiazem	1.6E-08 M	1.3E-08 M	1.2
**Ca^{2+} channel (L, verapamil site) (phenylalkylamine) (antagonist radioligand)**			
D 600	1.2E-08 M	5.9E-09 M	0.6
**Ca^{2+} channel (N) (antagonist radioligand)**			
ω-conotoxin GVIA	1.2E-12 M	5.1E-13 M	1.9
**SK_{Ca} channel (antagonist radioligand)**			
apamin	1.2E-11 M	5.9E-12 M	1.7
**Na^+ channel (site 2) (antagonist radioligand)**			
veratridine	3.9E-06 M	3.5E-06 M	1.0
**Cl^- channel (GABA-gated) (antagonist radioligand)**			
picrotoxinin	9.1E-08 M	7.5E-08 M	0.9
**norepinephrine transporter *(h)* (antagonist radioligand)**			
protriptyline	2.4E-09 M	1.8E-09 M	1.0

Reference Compound	IC_{50} (M)	K_i (M)	nH
**dopamine transporter *(h)* (antagonist radioligand)**			
BTCP	7.1E-09 M	3.8E-09 M	0.9
**GABA transporter (antagonist radioligand)**			
nipecotic acid	1.6E-06 M	1.6E-06 M	0.8
**choline transporter (CHT1) *(h)* (antagonist radioligand)**			
hemicholinium-3	6.5E-09 M	3.6E-09 M	0.7
**5-HT transporter *(h)* (antagonist radioligand)**			
imipramine	1.5E-09 M	6.9E-10 M	1.1

3.2. *In Vitro* Pharmacology: Enzyme and Uptake Assays

3.2.1. Test Compound Results

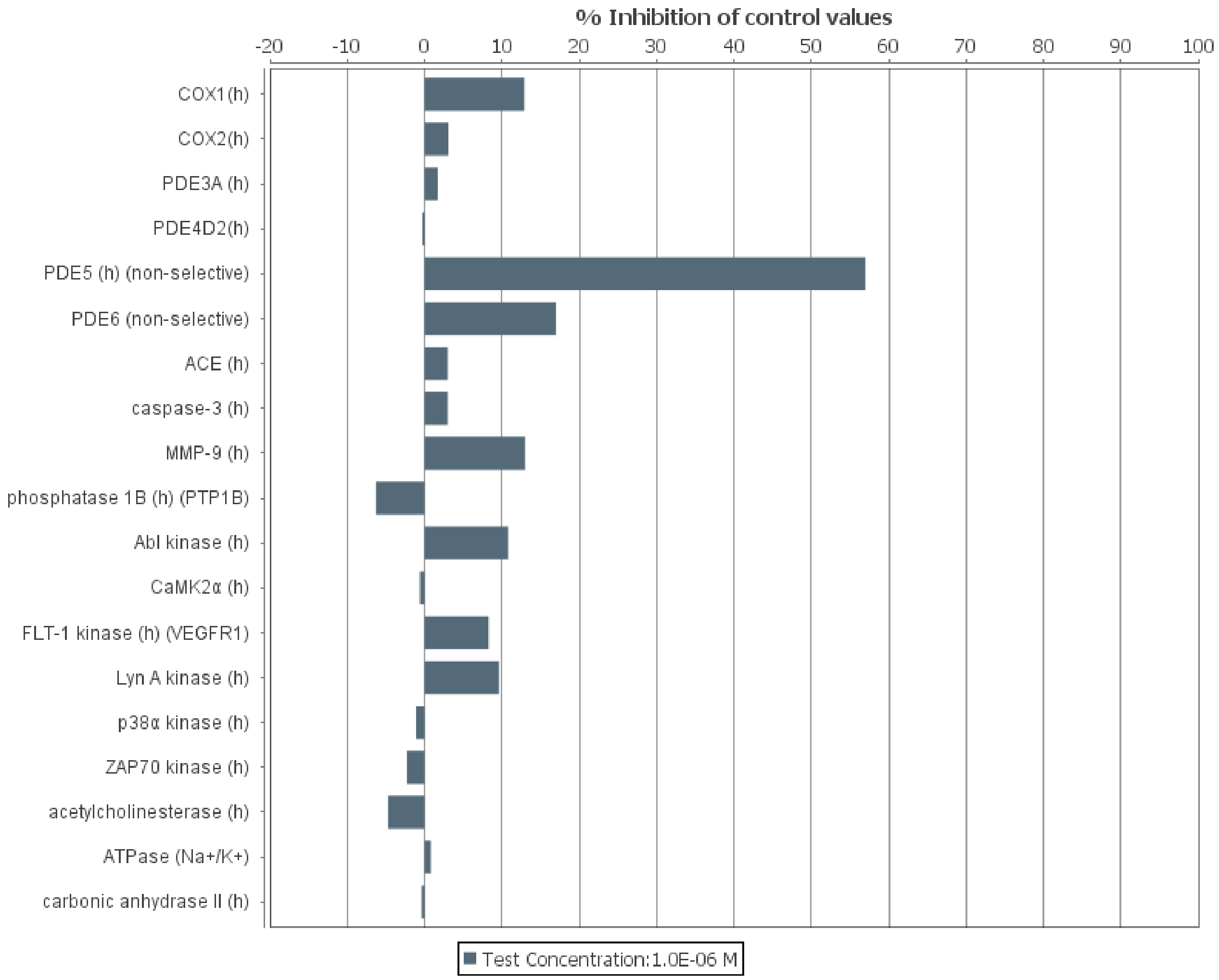

Figure 4. Histogram for HC-122608

Cerep Compound I.D.	Client Compound I.D.	Test Concentration	% Inhibition of control values		
			1st	2nd	Mean
**COX$_1$ *(h)***					
100004002-1	HC-122608	1.0E-06 M	-1.5	27.2	12.9
**COX$_2$ *(h)***					
100004002-1	HC-122608	1.0E-06 M	-5.6	11.8	3.1
**PDE3A *(h)***					
100004002-1	HC-122608	1.0E-06 M	3.1	0.2	1.7
**PDE4D$_2$ *(h)***					
100004002-1	HC-122608	1.0E-06 M	1.3	-2.0	-0.3
**PDE5 *(h)* (non-selective)**					
100004002-1	HC-122608	1.0E-06 M	54.6	59.4	**57.0**
**PDE6 (non-selective)**					
100004002-1	HC-122608	1.0E-06 M	25.3	8.8	17.0

Cerep Compound I.D.	Client Compound I.D.	Test Concentration	% Inhibition of control values		
			1st	2nd	Mean
**ACE *(h)***					
100004002-1	HC-122608	1.0E-06 M	0.0	6.0	3.0
**caspase-3 *(h)***					
100004002-1	HC-122608	1.0E-06 M	3.7	2.3	3.0
**MMP-9 *(h)***					
100004002-1	HC-122608	1.0E-06 M	8.7	17.2	13.0
**phosphatase 1B *(h)* (PTP1B)**					
100004002-1	HC-122608	1.0E-06 M	-6.7	-5.9	-6.3
**Abl kinase *(h)***					
100004002-1	HC-122608	1.0E-06 M	11.4	10.2	10.8
**CaMK2α *(h)***					
100004002-1	HC-122608	1.0E-06 M	-4.0	2.8	-0.6
**FLT-1 kinase *(h)* (VEGFR1)**					
100004002-1	HC-122608	1.0E-06 M	9.3	7.3	8.3
**Lyn A kinase *(h)***					
100004002-1	HC-122608	1.0E-06 M	13.7	5.5	9.6
**p38α kinase *(h)***					
100004002-1	HC-122608	1.0E-06 M	-0.9	-1.2	-1.1
**ZAP70 kinase *(h)***					
100004002-1	HC-122608	1.0E-06 M	-1.6	-3.0	-2.3
**acetylcholinesterase *(h)***					
100004002-1	HC-122608	1.0E-06 M	-5.9	-3.5	-4.7
**ATPase (Na^+/K^+)**					
100004002-1	HC-122608	1.0E-06 M	3.0	-1.5	0.8
**carbonic anhydrase II *(h)***					
100004002-1	HC-122608	1.0E-06 M	-0.9	0.2	-0.4

3.2.2. Reference Compound Results

Reference Compound	IC_{50} (M)	nH
**COX_1 *(h)***		
diclofenac	1.1E-08 M	1.8
**COX_2 *(h)***		
NS 398	1.6E-07 M	1.4
**PDE3A *(h)***		
milrinone	3.5E-07 M	1.0
**$PDE4D_2$ *(h)***		
rolipram	4.8E-08 M	0.7
**PDE5 *(h)* (non-selective)**		
dipyridamole	8.8E-07 M	1.0
**PDE6 (non-selective)**		
zaprinast	1.7E-07 M	0.7
**ACE *(h)***		
captopril	1.6E-09 M	1.2
**caspase-3 *(h)***		
Ac-DEVD-CHO	1.5E-09 M	1.2
**MMP-9 *(h)***		
GM6001	3.2E-10 M	1.6
**phosphatase 1B *(h)* (PTP1B)**		
ammonium molybdate	5.4E-07 M	2.9
**Abl kinase *(h)***		
staurosporine	8.3E-08 M	1.3
**CaMK2α *(h)***		
AIP	8.1E-08 M	1.0
**FLT-1 kinase *(h)* (VEGFR1)**		
staurosporine	1.5E-08 M	1.4

Reference Compound	IC_{50} (M)	nH
**Lyn A kinase *(h)***		
staurosporine	2.6E-08 M	2.1
**p38α kinase *(h)***		
SB202190	2.3E-08 M	1.4
**ZAP70 kinase *(h)***		
staurosporine	9.0E-08 M	1.2
**acetylcholinesterase *(h)***		
neostigmine	1.6E-08 M	1.3
**ATPase (Na^+/K^+)**		
ouabain	1.2E-06 M	1.3
**carbonic anhydrase II *(h)***		
acetazolamide	3.9E-08 M	1.8

4. RESULTS INTERPRETATION GUIDE

In Vitro Pharmacology

Results showing an inhibition (or stimulation for assays run in basal conditions) higher than 50% are considered to represent significant effects of the test compounds. 50% is the most common cut-off value for further investigation (determination of IC_{50} or EC_{50} values from concentration-response curves) that we would recommend.
Results showing an inhibition (or stimulation) between 25% and 50% are indicative of weak to moderate effects (in most assays, they should be confirmed by further testing as they are within a range where more inter-experimental variability can occur).
Results showing an inhibition (or stimulation) lower than 25% are not considered significant and mostly attributable to variability of the signal around the control level.
Low to moderate negative values have no real meaning and are attributable to variability of the signal around the control level. High negative values (≥ 50%) that are sometimes obtained with high concentrations of test compounds are generally attributable to non-specific effects of the test compounds in the assays. On rare occasion they could suggest an allosteric effect of the test compound.

5. STORAGE AND RETENTION OF RECORDS

All documents generated during the performance of the study (raw data, various records such as QA audit reports, an original copy of the study report, study plan…) will be archived by Cerep for a 10-year period after study completion. The access to the archives is restricted to authorized employees only.

The original final report provided to the sponsor will be kept by the sponsor under its sole responsibility.

6. MATERIALS AND METHODS

6.1. Experimental Conditions

6.1.1. *In Vitro* Pharmacology: Binding Assays

Assay	Source	Ligand	Conc.	Kd	Non Specific	Incubation	Detection Method	Bibl.
**Receptors**								
**A_1*(h)* (antagonist radioligand)**	human recombinant (CHO cells)	[^{3}H]DPCPX	1 nM	1.7 nM	DPCPX (1 µM)	60 min RT	Scintillation counting	245
**A_{2A}*(h)* (agonist radioligand)**	human recombinant (HEK-293 cells)	[^{3}H]CGS 21680	6 nM	27 nM	NECA (10 µM)	120 min RT	Scintillation counting	141
**A_3*(h)* (agonist radioligand)**	human recombinant (HEK-293 cells)	[^{125}I]AB-MECA	0.15 nM	0.22 nM	IB-MECA (1 µM)	120 min RT	Scintillation counting	206
**α_1 (non-selective) (antagonist radioligand)**	rat cerebral cortex	[^{3}H]prazosin	0.25 nM	0.09 nM	prazosin (0.5 µM)	60 min RT	Scintillation counting	88
**α_{2A} *(h)* (antagonist radioligand)**	human recombinant (CHO cells)	[^{3}H]RX 821002	1 nM	0.8 nM	(-)epinephrine (100 µM)	60 min RT	Scintillation counting	542
**α_{2B}*(h)* (antagonist radioligand)**	human recombinant (CHO cells)	[^{3}H]RX 821002	2.5 nM	5 nM	(-)epinephrine (100 µM)	60 min RT	Scintillation counting	56
**α_{2C} *(h)* (antagonist radioligand)**	human recombinant (CHO cells)	[^{3}H]RX 821002	2 nM	0.95 nM	(-)epinephrine (100 µM)	60 min RT	Scintillation counting	56
**β_1 *(h)* (agonist radioligand)**	human recombinant (HEK-293 cells)	[^{3}H](-)CGP 12177	0.3 nM	0.39 nM	alprenolol (50 µM)	60 min RT	Scintillation counting	548
**β_2 *(h)* (agonist radioligand)**	human recombinant (CHO cells)	[^{3}H](-)CGP 12177	0.3 nM	0.15 nM	alprenolol (50 µM)	120 min RT	Scintillation counting	794
**β_3*(h)* (antagonist radioligand)**	SK-N-MC cells (endogenous)	[^{125}I]CYP (+ 0.1 µM (-)propranolol)	0.6 nM	0.7 nM	(-)propranolol (1 mM)	90 min 37°C	Scintillation counting	52
**AT_1 *(h)* (antagonist radioligand)**	human recombinant (HEK-293 cells)	[^{125}I][Sar^1,Ile^8]-AT-II	0.05 nM	0.05 nM	angiotensin-II (10 µM)	120 min 37°C	Scintillation counting	776
**CB_1*(h)* (agonist radioligand)**	human recombinant (CHO cells)	[^{3}H]CP 55940	0.5 nM	3.5 nM	WIN 55212-2 (10 µM)	120 min 37°C	Scintillation counting	657
**CB_2*(h)* (agonist radioligand)**	human recombinant (CHO cells)	[^{3}H]WIN 55212-2	0.8 nM	1.5 nM	WIN 55212-2 (5 µM)	120 min 37°C	Scintillation counting	165
**CCK_1 (CCK_A) *(h)* (agonist radioligand)**	human recombinant (CHO cells)	[^{125}I]CCK-8s	0.08 nM	0.24 nM	CCK-8s (1 µM)	60 min RT	Scintillation counting	562
**CCK_2 (CCK_B) *(h)* (agonist radioligand)**	human recombinant (CHO cells)	[^{125}I]CCK-8s	0.08 nM	0.054 nM	CCK-8s (1 µM)	60 min RT	Scintillation counting	134
**D_1*(h)* (antagonist radioligand)**	human recombinant (CHO cells)	[^{3}H]SCH 23390	0.3 nM	0.2 nM	SCH 23390 (1 µM)	60 min RT	Scintillation counting	281
**D_{2S}*(h)* (antagonist radioligand)**	human recombinant (HEK-293 cells)	[^{3}H]methyl-spiperone	0.3 nM	0.15 nM	(+)butaclamol (10 µM)	60 min RT	Scintillation counting	87
**D_{2S}*(h)* (agonist radioligand)**	human recombinant (HEK-293 cells)	[^{3}H]7-OH-DPAT	1 nM	0.68 nM	butaclamol (10 µM)	60 min RT	Scintillation counting	87

Assay	Source	Ligand	Conc.	Kd	Non Specific	Incubation	Detection Method	Bibl.
**D_3*(h)*** **(antagonist radioligand)**	human recombinant (CHO cells)	[^{3}H]methyl-spiperone	0.3 nM	0.085 nM	(+)butaclamol (10 µM)	60 min RT	Scintillation counting	145
**$D_{4.4}$*(h)*** **(antagonist radioligand)**	human recombinant (CHO cells)	[^{3}H]methyl-spiperone	0.3 nM	0.19 nM	(+)butaclamol (10 µM)	60 min RT	Scintillation counting	252
**ET_A*(h)*** **(agonist radioligand)**	human recombinant (CHO cells)	[^{125}I]endothelin-1	0.03 nM	0.03 nM	endothelin-1 (100 nM)	120 min 37°C	Scintillation counting	30
**ET_B *(h)*** **(agonist radioligand)**	human recombinant (CHO cells)	[^{125}I]endothelin-1	0.03 nM	0.04 nM	endothelin-1 (0.1 µM)	120 min 37°C	Scintillation counting	541
**$GABA_{A1}$ *(h)* (α1,β2,γ2)** **(agonist radioligand)**	human recombinant (CHO cells)	[^{3}H]muscimol	15 nM	30 nM	muscimol (10 µM)	120 min RT	Scintillation counting	1096
**$GABA_{B(1b)}$*(h)*** **(antagonist radioligand)**	human recombinant (CHO cells)	[^{3}H]CGP 54626	1 nM	1 nM	CGP 52432 (100 µM)	120 min RT	Scintillation counting	508
**TNF-α *(h)*** **(agonist radioligand)**	U-937 cells	[^{125}I]TNF-α	0.1 nM	0.05 nM	TNF-α (10 nM)	120 min 4°C	Scintillation counting	26
**H_1*(h)*** **(antagonist radioligand)**	human recombinant (HEK-293 cells)	[^{3}H]pyrilamine	1 nM	1.7 nM	pyrilamine (1 µM)	60 min RT	Scintillation counting	492
**H_2*(h)*** **(antagonist radioligand)**	human recombinant (CHO cells)	[^{125}I]APT	0.075 nM	2.9 nM	tiotidine (100 µM)	120 min RT	Scintillation counting	540
**H_3*(h)*** **(agonist radioligand)**	human recombinant (CHO cells)	[^{3}H]N^{α}-Me-histamine	1 nM	0.32 nM	(R)α-Me-histamine (1 µM)	60 min RT	Scintillation counting	563
**H_4*(h)*** **(agonist radioligand)**	human recombinant (HEK-293 cells)	[^{3}H]histamine	10 nM	7.6 nM	imetit (1 µM)	60 min RT	Scintillation counting	631
**BLT_1 (LTB_4) *(h)*** **(agonist radioligand)**	human recombinant (CHO cells)	[^{3}H]LTB_4	0.2 nM	0.2 nM	LTB_4 (0.2 µM)	60 min RT	Scintillation counting	616
**$CysLT_1$ (LTD_4) *(h)*** **(agonist radioligand)**	human recombinant (CHO cells)	[^{3}H]LTD_4	0.3 nM	0.24 nM	LTD_4 (1 µM)	60 min RT	Scintillation counting	618
**MCH_1*(h)*** **(agonist radioligand)**	human recombinant (CHO cells)	[^{125}I][Phe^{13},Tyr^{19}]-MCH	0.1 nM	1 nM	human MCH (0.1 µM)	60 min RT	Scintillation counting	526
**MC_1** **(agonist radioligand)**	B16-F1 cells (endogenous)	[^{125}I]NDP-α-MSH	0.05 nM	0.05 nM	NDP-α-MSH (1 µM)	90 min RT	Scintillation counting	390
**MC_3*(h)*** **(agonist radioligand)**	human recombinant (CHO cells)	[^{125}I]NDP-α-MSH	0.075 nM	0.4 nM	NDP-α-MSH (1 µM)	60 min 37°C	Scintillation counting	211
**MC_4*(h)*** **(agonist radioligand)**	human recombinant (CHO cells)	[^{125}I]NDP-α-MSH	0.05 nM	0.54 nM	NDP-α-MSH (1 µM)	120 min 37°C	Scintillation counting	211
**MT_1 (ML_{1A}) *(h)*** **(agonist radioligand)**	human recombinant (CHO cells)	[^{125}I]2-iodomelatonin	0.01 nM	0.04 nM	melatonin (1 µM)	60 min RT	Scintillation counting	639
**MT_3 (ML_2)** **(agonist radioligand)**	hamster brain	[^{125}I]2-iodomelatonin	0.1 nM	4.8 nM	melatonin (30 µM)	60 min 4°C	Scintillation counting	186
**motilin *(h)*** **(agonist radioligand)**	human recombinant (CHO cells)	[^{125}I]motilin	0.05 nM	0.26 nM	[$Nleu^{13}$]-motilin (1 µM)	120 min RT	Scintillation counting	285
**M_1*(h)*** **(antagonist radioligand)**	human recombinant (CHO cells)	[^{3}H]pirenzepine	2 nM	13 nM	atropine (1 µM)	60 min RT	Scintillation counting	59
**M_2 *(h)*** **(antagonist radioligand)**	human recombinant (CHO cells)	[^{3}H]AF-DX 384	2 nM	4.6 nM	atropine (1 µM)	60 min RT	Scintillation counting	59
**M_3 *(h)*** **(antagonist radioligand)**	human recombinant (CHO cells)	[^{3}H]4-DAMP	0.2 nM	0.5 nM	atropine (1 µM)	60 min RT	Scintillation counting	546

Assay	Source	Ligand	Conc.	Kd	Non Specific	Incubation	Detection Method	Bibl.
**M_4*(h)*** **(antagonist radioligand)**	human recombinant (CHO cells)	$[^3H]$4-DAMP	0.2 nM	0.32 nM	atropine (1 µM)	60 min RT	Scintillation counting	59
**M_5*(h)*** **(antagonist radioligand)**	human recombinant (CHO cells)	$[^3H]$4-DAMP	0.3 nM	0.3 nM	atropine (1 µM)	60 min RT	Scintillation counting	59
**NK_1*(h)*** **(agonist radioligand)**	U-373MG cells (endogenous)	$[^{125}I]$BH-SP	0.15 nM	0.12 nM	$[Sar^9,Met(O_2)^{11}]$-SP (1 µM)	60 min RT	Scintillation counting	104
**NK_2*(h)*** **(agonist radioligand)**	human recombinant (CHO cells)	$[^{125}I]$NKA	0.1 nM	0.12 nM	$[Nleu^{10}]$-NKA (4-10) (300 nM)	60 min RT	Scintillation counting	3
**Y_1*(h)*** **(agonist radioligand)**	SK-N-MC cells (endogenous)	$[^{125}I]$peptide YY	0.025 nM	0.06 nM	NPY (1 µM)	120 min 37°C	Scintillation counting	391
**δ_2 (DOP) *(h)*** **(agonist radioligand)**	human recombinant (CHO cells)	$[^3H]$DADLE	0.5 nM	0.73 nM	naltrexone (10 µM)	120 min RT	Scintillation counting	501
**κ (KOP)** **(agonist radioligand)**	rat recombinant (CHO cells)	$[^3H]$U 69593	1 nM	2 nM	naloxone (10 µM)	60 min RT	Scintillation counting	771
**µ (MOP) *(h)*** **(agonist radioligand)**	human recombinant (HEK-293 cells)	$[^3H]$DAMGO	0.5 nM	0.35 nM	naloxone (10 µM)	120 min RT	Scintillation counting	260
**PPARγ *(h)*** **(agonist radioligand)**	human recombinant (*E. coli*)	$[^3H]$rosiglitazone	5 nM	5.7 nM	rosiglitazone (10 µM)	120 min 4°C	Scintillation counting	567
**5-HT_{1A}*(h)*** **(agonist radioligand)**	human recombinant (HEK-293 cells)	$[^3H]$8-OH-DPAT	0.3 nM	0.5 nM	8-OH-DPAT (10 µM)	60 min RT	Scintillation counting	164
**5-HT_{1B}** **(antagonist radioligand)**	rat cerebral cortex	$[^{125}I]$CYP (+ 30 µM isoproterenol)	0.1 nM	0.16 nM	serotonin (10 µM)	120 min 37°C	Scintillation counting	111
**5-HT_{1D}** **(agonist radioligand)**	rat recombinant (CHO cells)	$[^3H]$serotonin	1 nM	0.5 nM	serotonin (10 µM)	60 min RT	Scintillation counting	777
**5-HT_{2A}*(h)*** **(antagonist radioligand)**	human recombinant (HEK-293 cells)	$[^3H]$ketanserin	0.5 nM	0.6 nM	ketanserin (1 µM)	60 min RT	Scintillation counting	20
**5-HT_{2A}*(h)*** **(agonist radioligand)**	human recombinant (HEK-293 cells)	$[^{125}I]$(±)DOI	0.1 nM	0.3 nM	(±)DOI (1 µM)	60 min RT	Scintillation counting	288
**5-HT_{2B}*(h)*** **(agonist radioligand)**	human recombinant (CHO cells)	$[^{125}I]$(±)DOI	0.2 nM	0.2 nM	(±)DOI (1 µM)	60 min RT	Scintillation counting	571
**5-HT_{2C}*(h)*** **(antagonist radioligand)**	human recombinant (HEK-293 cells)	$[^3H]$mesulergine	1 nM	0.5 nM	RS 102221 (10 µM)	120 min 37°C	Scintillation counting	543
**5-HT_{2C}*(h)*** **(agonist radioligand)**	human recombinant (HEK-293 cells)	$[^{125}I]$(±)DOI	0.1 nM	0.9 nM	(±)DOI (10 µM)	60 min 37°C	Scintillation counting	288
**5-HT_{4e}*(h)*** **(antagonist radioligand)**	human recombinant (CHO cells)	$[^3H]$GR 113808	0.3 nM	0.15 nM	serotonin (100 µM)	60 min 37°C	Scintillation counting	309
**5-HT_6 *(h)*** **(agonist radioligand)**	human recombinant (CHO cells)	$[^3H]$LSD	2 nM	1.8 nM	serotonin (100 µM)	120 min 37°C	Scintillation counting	161
**5-HT_7 *(h)*** **(agonist radioligand)**	human recombinant (CHO cells)	$[^3H]$LSD	4 nM	2.3 nM	serotonin (10 µM)	120 min RT	Scintillation counting	217
**sigma (non-selective) (h)** **(agonist radioligand)**	cellules Jurkat (endogène)	[3H]DTG	10 nM	41 nM	Haloperidol (10 µM)	120 min RT	Scintillation counting	1136
**sst_4*(h)*** **(agonist radioligand)**	human recombinant (CHO cells)	$[^{125}I]Tyr^{11}$-somatostatin-14	0.1 nM	5.9 nM	somatostatin-14 (1 µM)	120 min RT	Scintillation counting	296

Assay	Source	Ligand	Conc.	Kd	Non Specific	Incubation	Detection Method	Bibl.
**GR *(h)* (agonist radioligand)**	IM-9 cells (cytosol)	[^{3}H]dexamethasone	1.5 nM	1.5 nM	triamcinolone (10 µM)	6 hr 4°C	Scintillation counting	283
**AR *(h)* (agonist radioligand)**	LNCaP cells (cytosol)	[^{3}H]methyltrienolone	1 nM	0.8 nM	mibolerone (1 µM)	24 hr 4°C	Scintillation counting	498
**TR (TH) (agonist radioligand)**	rat liver	[^{125}I]T_3	0.1 nM	0.24 nM	T_3 (1 µM)	20 hr 4°C	Scintillation counting	114
**UT *(h)* (agonist radioligand)**	human recombinant (CHO cells)	[^{125}I]urotensin-II	0.1 nM	0.29 nM	urotensin-II (3 µM)	120 min RT	Scintillation counting	622
**$VPAC_1$ (VIP_1) *(h)* (agonist radioligand)**	human recombinant (CHO cells)	[^{125}I]VIP	0.04 nM	0.05 nM	VIP (1 µM)	60 min RT	Scintillation counting	50
**V_{1a}*(h)* (agonist radioligand)**	human recombinant (CHO cells)	[^{3}H]AVP	0.3 nM	0.5 nM	AVP (1 µM)	60 min RT	Scintillation counting	343
**V_2*(h)* (agonist radioligand)**	human recombinant (CHO cells)	[^{3}H]AVP	0.3 nM	0.76 nM	AVP (1 µM)	120 min RT	Scintillation counting	343
**Ion channels**								
**BZD (central) (agonist radioligand)**	rat cerebral cortex	[^{3}H]flunitrazepam	0.4 nM	2.1 nM	diazepam (3 µM)	60 min 4°C	Scintillation counting	227
**AMPA (agonist radioligand)**	rat cerebral cortex	[^{3}H]AMPA	8 nM	82 nM	L-glutamate (1 mM)	60 min 4°C	Scintillation counting	166
**kainate (agonist radioligand)**	rat cerebral cortex	[^{3}H]kainic acid	5 nM	19 nM	L-glutamate (1 mM)	60 min 4°C	Scintillation counting	160
**NMDA (antagonist radioligand)**	rat cerebral cortex	[^{3}H]CGP 39653	5 nM	23 nM	L-glutamate (100 µM)	60 min 4°C	Scintillation counting	221
**glycine (strychnine-insensitive) (antagonist radioligand)**	rat cerebral cortex	[^{3}H]MDL 105,519	0.5 nM	5 nM	glycine (1 mM)	45 min 0°C	Scintillation counting	219
**PCP (antagonist radioligand)**	rat cerebral cortex	[^{3}H]TCP	10 nM	13 nM	MK 801 (10 µM)	120 min 37°C	Scintillation counting	257
**5-HT_3*(h)* (antagonist radioligand)**	human recombinant (CHO cells)	[^{3}H]BRL 43694	0.5 nM	1.15 nM	MDL 72222 (10 µM)	120 min RT	Scintillation counting	109
**Ca^{2+} channel (L, diltiazem site) (benzothiazepines) (antagonist radioligand)**	rat cerebral cortex	[^{3}H]diltiazem	15 nM	52 nM	diltiazem (10 µM)	120 min RT	Scintillation counting	212
**Ca^{2+} channel (L, verapamil site) (phenylalkylamine) (antagonist radioligand)**	rat cerebral cortex	[^{3}H]D888	3 nM	3 nM	D 600 (10 µM)	120 min RT	Scintillation counting	194
**Ca^{2+} channel (N) (antagonist radioligand)**	rat cerebral cortex	[^{125}I]ω-conotoxin GVIA	0.001 nM	0.0007 nM	ω-conotoxin GVIA (10 nM)	30 min RT	Scintillation counting	259
**SK_{Ca} channel (antagonist radioligand)**	rat cerebral cortex	[^{125}I]apamin	0.007 nM	0.007 nM	apamin (100 nM)	60 min 4°C	Scintillation counting	112
**Na^{+} channel (site 2) (antagonist radioligand)**	rat cerebral cortex	[^{3}H]batrachotoxinin	10 nM	91 nM	veratridine (300 µM)	60 min 37°C	Scintillation counting	28
**Cl^{-} channel (GABA-gated) (antagonist radioligand)**	rat cerebral cortex	[^{35}S]TBPS	3 nM	14.6 nM	picrotoxinin (20 µM)	120 min RT	Scintillation counting	136
**Transporters**								
**norepinephrine transporter *(h)* (antagonist radioligand)**	human recombinant (CHO cells)	[^{3}H]nisoxetine	1 nM	2.9 nM	desipramine (1 µM)	120 min 4°C	Scintillation counting	180
**dopamine transporter *(h)* (antagonist radioligand)**	human recombinant (CHO cells)	[^{3}H]BTCP	4 nM	4.5 nM	BTCP (10 µM)	120 min 4°C	Scintillation counting	190
**GABA transporter (antagonist radioligand)**	rat cerebral cortex	[^{3}H]GABA (+ 10 µM isoguvacine) (+ 10 µM baclofen)	10 nM	4600 nM	GABA (1 mM)	30 min RT	Scintillation counting	214
**choline transporter (CHT1) *(h)* (antagonist radioligand)**	human recombinant (CHO cells)	[^{3}H]hemicholinium-3	3 nM	3.9 nM	hemicholinium-3 (10 µM)	60 min RT	Scintillation counting	648

Assay	Source	Ligand	Conc.	Kd	Non Specific	Incubation	Detection Method	Bibl.
**5-HT transporter *(h)*** **(antagonist radioligand)**	human recombinant (CHO cells)	[^{3}H]imipramine	2 nM	1.7 nM	imipramine (10 µM)	60 min RT	Scintillation counting	566
**Other enzymes**								
**MAO-A** **(antagonist radioligand)**	rat cerebral cortex	[^{3}H]Ro 41-1049	10 nM	14 nM	clorgyline (1 µM)	60 min 37°C	Scintillation counting	36
**rolipram** **(antagonist radioligand)**	mouse brain	[^{3}H]rolipram	1 nM	1 nM	rolipram (10 µM)	60 min 4°C	Scintillation counting	60

6.1.2. *In Vitro* Pharmacology: Enzyme and Uptake Assays

Assay	Source	Substrate/Stimulus/Tracer	Incubation	Measured Component	Detection Method	Bibl.
**Kinases**						
**Abl kinase *(h)***	human recombinant (insect cells)	ATP + Ulight-TK peptide (100 nM)	60 min RT	phospho-Ulight-TK peptide	LANCE	556
**CaMK2α *(h)***	human recombinant	ATP + Ulight-CGSGSGRPRTSSFAEG (50 nM)	30 min RT	phospho-Ulight-CGSGSGRPRTSSFAEG	LANCE	647
**FLT-1 kinase *(h)* (VEGFR1)**	human recombinant (Sf9 cells)	ATP + Ulight-TK peptide (100 nM)	15 min RT	phospho-Ulight-TK peptide	LANCE	650
**Lyn A kinase *(h)***	human recombinant (insect cells)	ATP + biotinyl-βAβAβAKVEKIGEGTYGVVYK (400 nM)	90 min RT	phospho-biotinyl-βAβAβAKVEKIGEGTYGVVYK	HTRF	41
**p38α kinase *(h)***	human recombinant (*E. coli*)	ATP + Ulight-CFFKNIVTPRTPPPSQGK-amide (100 nM)	60 min RT	phospho-Ulight-CFFKNIVTPRTPPPSQGK-amide	LANCE	620
**ZAP70 kinase *(h)***	human recombinant (insect cells)	ATP + biotinyl-βAβAβADEEEYFIPP (2 µM)	15 min RT	phospho-biotinyl-βAβAβADEEEYFIPP	HTRF	556
**Other enzymes**						
**COX_1*(h)***	human recombinant (Sf9 cells)	arachidonic acid (4 µM)	5 min RT	PGE_2	HTRF	486
**COX_2*(h)***	human recombinant (Sf9 cells)	arachidonic acid (2 µM)	5 min RT	PGE_2	HTRF	486
**PDE3A *(h)***	human recombinant (Sf9 cells)	cAMP (40 nM)	30 min RT	residual cAMP	HTRF	902
**$PDE4D_2$*(h)***	human recombinant (Sf9 cells)	cAMP (40 nM)	15 min 37°C	residual cAMP	HTRF	906
**PDE5 *(h)* (non-selective)**	human platelets	[^{3}H]cGMP + cGMP (1 µM)	60 min RT	[^{3}H]5'GMP	Scintillation counting	263
**PDE6 (non-selective)**	bovine retina	[^{3}H]cGMP + cGMP (2 µM)	60 min RT	[^{3}H]5'GMP	Scintillation counting	306
**caspase-3 *(h)***	human recombinant (*E.coli*)	benzyloxycarbonyl-Asp-Glu-Val-Asp-AFC (3.6 µM)	60 min RT	AFC	Fluorimetry	476
**MMP-9 *(h)***	human recombinant	NFF-2 (10 µM)	90 min 37°C	Mca-Arg-Pro-Lys-Pro-Tyr-Ala	Fluorimetry	297
**phosphatase 1B *(h)* (PTP1B)**	human recombinant (*E. coli*)	DIFMUP (100 µM)	30 min RT	DIFMU	Fluorimetry	928
**acetylcholinesterase *(h)***	human recombinant (HEK-293 cells)	AMTCh (400 µM)	30 min RT	5 thio 2 nitrobenzoic acid	Photometry	63
**ATPase (Na^+/K^+)**	porcine cerebral cortex	ATP (2 mM)	60 min 37°C	Pi	Photometry	71
**carbonic anhydrase II *(h)***	human erythrocytes	4-nitrophenyl acetate (450 µM)	20 min RT	4-nitrophenol	Photometry	923

6.2. Analysis and expression of results

6.2.1. *In Vitro* Pharmacology: Binding Assays

The results are expressed as a percent of control specific binding

$$\frac{\text{measured specific binding}}{\text{control specific binding}} * 100$$

and as a percent inhibition of control specific binding

$$100 - \left(\frac{\text{measured specific binding}}{\text{control specific binding}} * 100\right)$$

obtained in the presence of HC-122608.

REDACTED

6.2.2. *In Vitro* Pharmacology: Enzyme and Uptake Assays

The results are expressed as a percent of control specific activity

$$\frac{\text{measured specific activity}}{\text{control specific activity}} * 100$$

and as a percent inhibition of control specific activity

$$100 - \left(\frac{\text{measured specific activity}}{\text{control specific activity}} * 100\right)$$

obtained in the presence of HC-122608.

REDACTED

7. BIBLIOGRAPHY

3. Aharony, D. et al. (1993), *Mol. Pharmacol.,* 44 *: 356-363.*
20. Bonhaus, D.W. et al. (1995), *Brit. J. Pharmacol.,* 115 *: 622-628.*
26. Brockhaus, M. et al. (1990), *Proc. Natl. Acad. Sci. U.S.A.,* 87 *: 3127-3131.*
28. Brown, G.B. (1986), *J. Neurosci.,* 6 *: 2064-2070.*
30. Buchan, K.W. et al. (1994), *Brit. J. Pharmacol.,* 112 *: 1251-1257.*
36. Cesura, A.M. et al. (1990), *Mol. Pharmacol.,* 37 *: 358-366.*
41. Cheng , H.C. et al. (1992), *J. Biol. Chem.,* 267 *: 9248-9256.*
50. Couvineau, A. et al. (1985), *Biochem. J.,* 231 *: 139-143.*
52. Curran, P.K. and Fishman, P.H. (1996), *Cell Signal.,* 8 *: 355-364.*
56. Devedjian, J.C. et al. (1994), *Eur. J. Pharmacol.,* 252 *: 43-49.*
59. Dorje, F. et al. (1991), *J. Pharmacol. Exp. Ther.,* 256 *: 727-733.*
60. Duplantier, A.J. et al. (1996), *J. Med. Chem.,* 39 *: 120-125.*
63. Ellman, G.L. et al. (1961), *Biochem. Pharmacol.,* 7 *: 88-95.*
71. Fiske, C.M. and Subbarow, Y. (1925), *J. Biol. Chem.,* 66 *: 375-400.*
87. Grandy, D.K. et al. (1989), *Proc. Natl. Acad. Sci. U.S.A.,* 86 *: 9762-9766.*
88. Greengrass, P. and Bremner, R. (1979), *Eur. J. Pharmacol.,* 55 *: 323-326.*
104. Heuillet, E. et al. (1993), *J. Neurochem.,* 60 *: 868-876.*
109. Hope, A.G. et al. (1996), *Brit. J. Pharmacol.,* 118 *: 1237-1245.*
111. Hoyer, D. et al. (1985), *Eur. J. Pharmacol.,* 118 *: 1-12.*
112. Hugues, M. et al. (1982), *J. Biol. Chem.,* 257 *: 2762-2769.*
114. Inoue, A. et al. (1983), *Anal. Biochem.,* 134 *: 176-183.*
134. Lee, Y.M. et al. (1993), *J. Biol. Chem.,* 268 *: 8164-8169.*
136. Lewin, A.H. et al. (1989), *Mol. Pharmacol.,* 35 *: 189-194.*
141. Luthin, D.R. et al. (1995), *Mol. Pharmacol.,* 47 *: 307-313.*
145. Mackenzie, R.G. et al. (1994), *Eur. J. Pharmacol.,* 266 *: 79-85.*
160. Monaghan, D.T. and Cotman, C.W. (1982), *Brain Res.,* 252 *: 91-100.*
161. Monsma, F.J. et al. (1993), *Mol. Pharmacol.,* 43 *: 320-327.*
164. Mulheron, J.G. et al. (1994), *J. Biol. Chem.,* 269 *: 12954-12962.*
165. Munro, S. et al. (1993), *Nature,* 365 *: 61-65.*
166. Murphy, D.E. et al. (1987), *Neurochem. Res.,* 12 *: 775-781.*
180. Pacholczyk, T. et al. (1991), *Nature,* 350 *: 350-354.*
186. Pickering, D.S. and Niles, L.P. (1990), *Eur. J. Pharmacol.,* 175 *: 71-77.*
190. Pristupa, Z.B. et al. (1994), *Mol. Pharmacol.,* 45 *: 125-135.*
194. Reynolds, I.J. et al. (1986), *J. Pharmacol. Exp. Ther.,* 237 *: 731-738.*
206. Salvatore, C.A. et al. (1993), *Proc. Natl. Acad. Sci. U.S.A.,* 90 *: 10365-10369.*
211. Schioth, H.B. et al. (1997), *Neuropeptides,* 31 *: 565-571.*
212. Schoemaker, H. and Langer, S.Z. (1985), *Eur. J. Pharmacol.,* 111 *: 273-277.*
214. Shank, R.P. et al. (1990), *J. Neurochem.,* 54 *: 2007-2015.*
217. Shen, Y. et al. (1993), *J. Biol. Chem.,* 268 *: 18200-18204.*
219. Baron, B.M. et al. (1996), *J. Pharmacol. Exp. Ther.,* 279 *: 62-68.*
221. Sills, M.A. et al. (1991), *Eur. J. Pharmacol.,* 192 *: 19-24.*
227. Speth, R.C. et al. (1979), *Life Sci.,* 24 *: 351-358.*
245. Townsend-Nicholson, A. and Schofield, P.R. (1994), *J. Biol. Chem.,* 269 *: 2373-2376.*
252. Van Tol, H.H.M. et al. (1992), *Nature,* 358 *: 149-152.*
257. Vignon, J. et al. (1986), *Brain Res.,* 378 *: 133-141.*
259. Wagner, J.A. et al. (1988), *J. Neurosci.,* 8 *: 3354-3359.*
260. Wang, J.B. et al. (1994), *FEBS Lett.,* 338 *: 217-222.*
263. Weishaar, R.E. et al. (1986), *Biochem. Pharmacol.,* 35 *: 787-800.*
281. Zhou, Q.Y. et al. (1990), *Nature,* 347 *: 76-80.*
283. Clark, A.F. et al. (1996), *Invest. Ophtalmol. Vis. Sci.,* 37 *: 805-813.*
285. Feighner, S.D. et al. (1999), *Science,* 284 *: 2184-2188.*
288. Bryant, H.U. et al. (1996), *Life Sci.,* 15 *: 1259-1268.*
296. Rohrer, L. et al. (1993), *Proc. Natl. Acad. Sci. U.S.A .,* 90 *: 4196-4200.*
297. Nagase, N. et al. (1994), *J. Biol. Chem.,* 269 *: 20952-20957.*
306. Ballard, A.S. et al. (1998), *J. Urol.,* 159 *: 2164-2171.*
309. Mialet, J. et al. (2000), *Brit. J. Pharmacol.,* 129 *: 771-781.*
343. Tahara, A. et al. (1998), *Brit. J. Pharmacol.,* 125 *: 1463-1470.*
390. Siegrist, W. et al. (1988), *J. Recep. Res.,* 8 *: 323-343.*
391. Wieland, H. A. et al. (1995), *J. Pharmacol. Exp. Ther.,* 275 *: 143-149.*
476. Mittl, P.R.E. et al. (1997), *J. Biol. Chem.,* 272 *: 6539-6547.*
486. Glaser, K. et al. (1995), *Eur. J. Pharmacol.,* 281 *: 107-111.*
492. Smit, M.J. et al. (1996), *Brit. J. Pharmacol.,* 117 *: 1071-1080.*
498. Zava, D.T. et al. (1979), *Endocrinology,* 104 *: 1007-1012.*
501. Simonin, F. et al. (1994), *Mol. Pharmacol.,* 46 *: 1015-1021.*
508. Green, A. et al. (2000), *Brit. J. Pharmacol.,* 131 *: 1766-1774.*
526. Mac Donald, D. et al. (2000), *Mol. Pharmacol.,* 58 *: 217-225.*
540. Leurs, R. et al. (1994), *Brit. J. Pharmacol.,* 112 *: 847-854.*
541. Fuchs, S. et al. (2001), *Mol. Med.,* 7 *: 115-124.*
542. Langin, D. et al. (1989), *Eur. J. Pharmacol.,* 167 *: 95-104.*
543. Stam, N.J. et al. (1994), *Eur. J. Pharmacol.,* 269 *: 339-348.*
546. Peralta, E. G. et al. (1987), *Embo. J.,* 6 *: 3923-3929.*
548. Levin, M.C. et al. (2002), *J. Biol.Chem.,* 277 *: 30429-30435.*
556. Park, Y.M. et al. (1999), *Anal. Biochem.,* 269 *: 94-104.*
562. Bignon, E. et al. (1999), *J. Pharmacol. Exp. Ther.* 289 *: 742-751.*
563. Lovenberg, T.W. et al. (1999), *Mol. Pharmacol.,* 55 *: 1101-1107.*
566. Tatsumi, M. et al. (1999), *Eur. J. Pharmacol.,* 368 *: 277-283.*
567. Ferry, G. et al. (2001), *Eur. J. Pharmacol.,* 417 *: 77-89.*
571. Choi, D.S. et al. (1994), *FEBS Lett.,* 352 *: 393-399.*
616. Yokomizo, T. et al. (2001), *J. Biol. Chem.,* 276 *: 12454-12459.*
618. Martin, V. et al. (2001), *Biochem. Pharmacol.,* 62 *: 1193-1200.*
620. Frantz, B. et al. (1998), *Biochemistry,* 37 *: 13846-13853.*
622. Maguire, J.J. et al. (2000), *Brit. J. Pharmacol.,* 131 *: 441-446.*
631. Liu, C. et al. (2001), *J. Pharmacol. Exp. Ther.,* 299 *: 121-130.*
639. Witt-Enderby, P.A. and Dubocovich, M.L. (1996), *Mol. Pharmacol.,* 50 *: 166-174.*
647. Ichida, A. and Fujisawa, H. (1995), *J. Biol. Chem.,* 270 *: 2163-2170.*
648. Apparsundaram, S. et al. (2000), *Biochem. Biophys. Res. Commun.,* 276 *: 862-867.*
650. Itokawa, T. et al. (2002), *Mol. Cancer Ther.,* 1 *: 295-302.*
657. Rinaldi-Carmona, M. et al. (1996), *J. Pharmacol. Exp. Ther.,* 278 *: 871-878.*
771. Meng, F. et al. (1993), *Proc. Natl. Acad. Sci. U.S.A..,* 90 *: 9954-9958.*
776. Le, M.T. et al. (2005), *Eur. J. Pharmacol.,* 513 *: 35-45.*
777. Wurch, T. et al. (1997), *J. Neurochem.,* 68 *: 410-418.*
794. Joseph, S.S. et al. (2004), *Naun.-Sch. Arch. Pharm.,* 369 *: 525-532.*
902. Bender, A.T. and Beavo, J.A. (2006), *Pharmacol. Rev.,* 58 *: 488-520.*
906. Saldou, N. et al. (1998), *Cell Signal.,* 10 *: 427-440.*
923. Iyer, R. et al. (2006), *J. Biomol. Screen.,* 11 *: 782-791.*
928. Welte, S. et al. (2005), *Anal. Biochem.,* 338 *: 32-38.*
1096. Wang, X.K. (2001), *Acta. Pharmacol. Sin.,* 22 *: 521-523.*
1128. Fernandes, T. et al. (2010), *Braz J Med Biol Res.,* 43 *: 837-842*
1136. GANAPATHY ME. et al. (1999), *JPET,* 289 *: 251-260*
